# Ru(ii)arene(N^N bpy/phen)-based RAPTA complexes for *in vitro* anti-tumour activity in human glioblastoma cancer cell lines and *in vivo* toxicity studies in a zebrafish model†

**DOI:** 10.1039/d2ra02677e

**Published:** 2022-06-29

**Authors:** Anuja P. K., Binoy Kar, Nilmadhab Roy, Priyankar Paira

**Affiliations:** Department of Chemistry, School of Advanced Sciences, Vellore Institute of Technology Vellore-632014 Tamilnadu India priyankar.paira@vit.ac.in

## Abstract

Herein, we have introduced a series of half-sandwich Ru(ii)arene(N^N bpy/phen)-based RAPTA complexes for brain cancer therapy. Among all the synthesized complexes, [(η^6^-*p*-cymene)Ru^II^(κ^2^-*N*,*N*-4,7dimethyl phenanthroline)(PTA)]·2PF_6_ (4c) and [(η^6^-*p*-cymene)Ru^II^(κ^2^-*N*,*N*-4,7diphenyl phenanthroline)(PTA)]·2PF_6_ (4d) showed outstanding potency against the T98G, LN229 and U87MG cancer cells. The antiproliferative activity of these complexes was reinforced by neurosphere, DNA intercalation, agarose gel electrophoresis, cell cycle analysis and time-dependent ROS detection assays. The real-time reverse transcription (RT)-polymerase chain reaction (PCR) study showed that complex 4c inhibited the TNF-α-induced NF-κB phosphorylation in glioma cells. Moreover, the *in vivo* biodistribution of complex 4c in different organs and the morphological patterns of widely used zebrafish embryos due to toxic effects have been evaluated.

## Abstract 2

A series of half-sandwich Ru(ii)arene(N^N bpy/phen)-based RAPTA complexes have been developed for brain cancer therapy.

## Graphical abstract

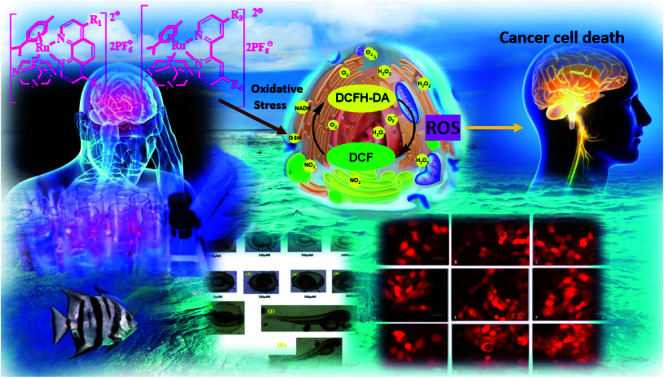

## Introduction

In the quest to find pertinent anticancer chemotherapeutics to restrain the proliferation of malignant tumours, inorganic metal complexes have ignited the light of hope to reveal remarkable results as pharmaceuticals for healing the most pernicious disease, cancer. The anticancer competencies of metallo-therapeutics are due to their ability to hinder cancer cell division and thereby accelerate the apoptosis of cancer cells, triggering DNA damage as well as interrupting the repair mechanism of DNA.^1–3^ Glioblastoma (GBM) is the most common and aggressive class of primary brain tumours, having a dismal prognosis. Despite standard-of-care treatment, GBM is among the most resistant cancers to radiation and cytotoxic chemotherapy, thus remaining an extremely heterogeneous and incurable disease, with an overall median survival of 15 months.^4,5^ Although many targeted pharmacological agents have been developed to improve current therapies, a great majority of these drugs have not achieved long-term remissions when tested in animals or even in clinical trials, causing treatment options to still be limited.^6^ With the challenge of developing more effective therapeutic strategies, we are mostly interested in metal complexes in our current models of study. Furthermore, metal complexes serve as an important class of molecules in oncology, being well-suited for theranostic drugs as well as diagnostic and therapeutic applications.^7^ The magnificence of metal complexes has captivated the minds of researchers after the fortuitous discovery of cisplatin, *cis*-[Pt(NH_3_)_2_Cl_2_], the currently most renowned platinum-based anticancer metallodrug, which has enlightened the field of research and development based on anticancer metallodrugs, bringing about an immediate revolution in cancer therapy.^8,9^ These systems have emerged as genotoxic for irreversibly binding to DNA, and thus cisplatin has been very prominent in creating platinated intrastrand-like lesions after being coordinated to the contiguous G-sites, facilitating DNA repair mechanisms that lead to mitochondrial-mediated apoptosis in the due course of time.^10^ However, several harmful dose-limiting side effects as well as tumour resistance are reducing the popularity of cisplatin at the present time. In this regard, ruthenium complexes have come into focus, being adorned with prosperous anticancer activity, offering lower toxicity and being effective against Pt-resistant tumours. The early success of NAMI-A ((ImH)[*trans*-RuCl_4_(dmso-S)(Im)], Im = imidazole) and KP1019/1339 (KP1019 = (IndH)[*trans*-RuCl_4_(Ind)_2_], Ind = indazole; KP1339 = Na[*trans*-RuCl_4_(Ind)_2_]), therefore, led to extensive exploration of ruthenium compounds, confirming their acceptance in the arena of cancer chemotherapy.^11,12^ Ruthenium complexes are considered a delightful choice for cancer therapy due to several reasons: (a) they constitute thermodynamically stable coordination compounds with a low rate of ligand exchange, enabling them to reach specific biological targets without being altered; (b) they display variable oxidation states that are stable under physiological conditions; and (c) they are capable of imitating iron for transportation after binding with various biomolecules, affording low toxicities.^13^ In 2004, a new class of RAPTA (ruthenium(ii)arene PTA) complexes associated with phosphaadamantane as well as arene ligands were developed, where PTA stands for 1,3,5-triaza-7-phosphaadamantane.^14^ It can be shown that RAPTA-C can bring on anticancer activities against Ehrlich ascites carcinoma (EAC) through the action of p53-JNK creating mitochondrial dysfunction.

**Fig. 1:**
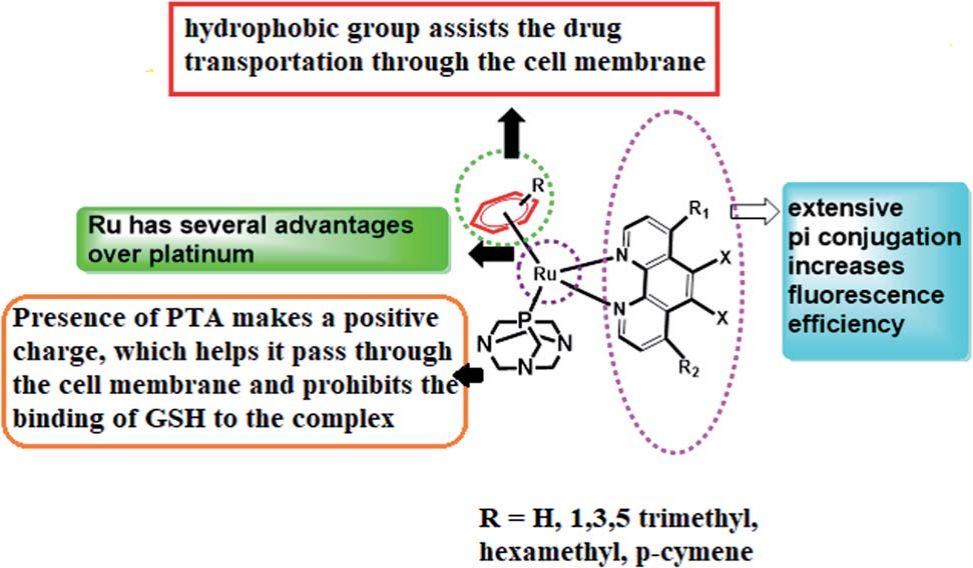
Design of the [Ru^II^(η^6^-*p*-cymene)N^N bpy/phen(pta)] complex.

RAPTA-T was developed to uncover the anti-invasive and anti-metastatic activities toward breast cancer cells. In contrast to classic phosphine ligands, RAPTA complexes are stable in air, possessing remarkable thermodynamic stability and GSH solubility along with significant aqueous solubility and stability.^15^ Therefore, the introduction of the PTA ligand in ruthenium complexes can enhance the reactivity towards DNA over the platinum complexes and therefore they can easily induce DNA damage. However, at physiological pH or higher, they remain almost nonreactive to the DNA of healthy cells but they expose their activity at reduced pH (<7) and thereby accomplish the damage of DNA.^16^ In spite of these remarkable effects of ruthenium complexes, the thirst for new arene ruthenium complexes with σ-bonded aromatic ligands capable of binding to DNA either by metal coordination or by the intercalation of an appended aromatic ligand triggered the development of ample Ru–arene complexes.^17,18^ In line with this, it has also been seen in our earlier work that the presence of pyridine and phenanthroline moieties can emphasize the cancer-inhibiting efficiency of these complexes.^19^ Therefore, we have aspired to design RAPTA complexes of arene bipyridine and phenanthroline by coordinating with Ru(ii) metal in order to evaluate their bioimaging and *in vivo* cytotoxic efficiency (Fig. 1).

## Results and discussion

### Synthesis and characterization

**Scheme 1:**
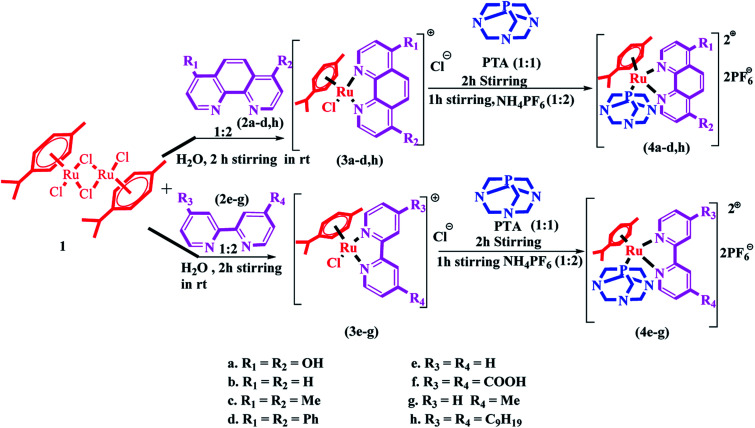
Synthesis of the [Ru^II^(η^6^-*p*-cymene)(N^N)(pta)] complexes (4a–h).

The synthesis of Ru(ii)–arene PTA complexes (4a–h) is summarized in Scheme 1. At the outset, bipyridine and phenanthroline (2a–h) and [(η^6^-*p*-cymene)RuCl(μ-Cl)]_2_ were treated for 2 h in water, followed by the addition of 1,3,5-triaza-7-phosphaadamantane (PTA) and stirring was continued for another 2 h (Scheme 1). The counter chloride ion was exchanged with ammonium hexafluorophosphate (NH_4_PF_6_). The crude RAPTA complexes (4a–h) were recrystallized with methanol-diethyl ether mixture *via* the vapor diffusion method and crystalline products were obtained in high yields. These complexes were analyzed by traditional spectroscopic techniques. The ^1^H NMR spectrum of complex 4a revealed the peaks of 12 protons present in PTA ligands at *δ* 3.6–4.4 ppm. ^13^C NMR spectra displayed all the peaks for the aliphatic carbons of *p*-cymene as well as PTA in the range of *δ* 25–78 ppm. In the range of *δ* −31 to −34 ppm, the usual peaks of phosphorus present in the PTA ligand were observed. Again, ESI-MS confirmed the formation of complex 4a by the peak for [M-2PF_6_]^2+^ at *m*/*z*: 302.7.

### Photophysical study

The photophysical properties of RAPTA derivatives (4a–h) were investigated in a DMSO–water (1 : 9, v/v) mixture. All these complexes revealed the absorption band at 250–300 nm indicating the π–π* transitions that occurred in bipyridine and phenanthroline ligands. Moreover, a weak transition band in the 300–400 nm region was observed in these complexes due to metal-to-ligand charge transfer (MLCT). The emission spectra were observed in the region of 325–500 nm, exhibiting very negligible variation in intensity with the substitution of different ligands. Complex 4a having the –OH substituted phenanthroline ligand showed the highest emission intensity along with the highest *λ*_ems_ in the range of 375–500 nm. Complexes 4d and 4h exhibited the highest quantum yield (0.018) among the complexes (Fig. S1 and Table S1†).

### Investigation of solubility, conductivity and lipophilicity

The tumour-preventing potential of metal complexes is predominantly maintained through the equilibrium between hydrophilicity and lipophilicity. It was seen that the prepared RAPTA complexes (4a–h) showed excellent solubility in DMF, DMSO, and MeOH. The solubility range of 5–10 mg ml^−1^ was observed for all these complexes in 10% DMEM–DMSO medium. The lipophilicity of all these complexes was explored by the shake-flask method, measuring the *n*-octanol/water partition coefficient (log *P*_o/w_) to establish drug-like behaviour and the values were obtained in the range of 0.3–1.4 (Table S1†). The highest partition coefficient value was identified for complex 4e compared to the other complexes. The conductive character of these complexes was measured in fresh DMSO as well as in 10% aqueous DMSO. These RAPTA complexes exhibited a similar range (71–78 S cm^2^ mol^−1^) of molar conductance in fresh DMSO as well as in 10% aqueous DMSO confirming their 1 : 2 electrolytic nature. Hence, the observed conductivity was ascribed to the insignificant detachment of the M-PTA or M-*p*-cymene bond, which endorses the stability of these complexes in aqueous medium (Table S1†).

### DNA binding study

The DNA binding proficiency of RAPTA complexes was explored by applying the concept of electronic absorption titration. The binding propensity of the RAPTA complex, 4c with Ct-DNA, was investigated using UV-visible studies. An absorption band in the range of 260–290 nm was observed for well-ordered Ct-DNA having the absorption maxima at 258 nm, indicating the π–π* transitions in purine and pyrimidine base pairs. With the complex 4c–DNA interaction, it exhibited a hyperchromic effect in both Ru(dπ)-dphen (π*) (260–300 nm) and MLCT (350–500 nm) due to the denaturation of the DNA duplex and the complex remained intact, with DNA allowing the absorption of more light by the complex (Fig. S2†). From the plot of [DNA]/(*ε*_a_ − *ε*_f_) *vs.* [DNA] the intrinsic binding constant (*K*_b_) was recognized for the specified 4c–DNA conjugate. The *K*_b_ value for complex 4c with DNA was found to be 1.38 ± 0.28 × 10^5^ M^−1^ (Table S2†), which was somewhat lower than the typical DNA intercalator, EtBr (*K*_EtBr_ = 7 × 10^5^ M^−1^).

### EtBr displacement assay

The fluorescence intensity of EtBr-bound DNA was found to be substantially decreased with the gradual addition of complex 4c, which marked the intercalative nature of the complex (Fig. S2†). RAPTA complex 4c showed emission in the range of 590–700 nm upon excitation at 485 nm. Therefore, significantly, *K*_app_ and *K*_sv_ of the selected complex were found to be 1.38 × 10^6^ M^−1^ and 0.018 × 10^4^ M^−1^, respectively, whereas the *K*_EtBr_ value was found to be 1 × 10^7^ M^−1^ from literature (Table S2†).^20^

### Viscosity measurement

The hydrodynamic study of the viscosity was conducted to screen the binding nature of the designated complexes 4c and 4d with Ct-DNA. The obtained results exposed a steady escalation in the relative viscosity upon increasing the concentration of 4d with respect to EtBr (Fig. S3†). Interestingly, complex 4c exhibited an abrupt increase in viscosity up to *r*_i_ = 2 and a moderate increase was observed beyond this. The results revealed that complex 4d exhibited a higher intercalation propensity as compared to 4c (Fig. S3†).

### DNA-cleaving study

**Fig. 2:**
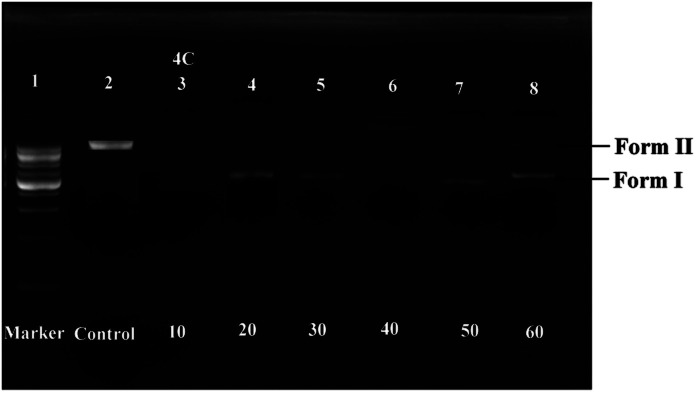
DNA degradation study of complex 4c [lane 1 − 1 kb plasmid DNA marker, lane 2 – plasmid DNA, lane 3–8 – plasmid DNA with complex 4c at different concentrations (10–60 μM)].

Agarose gel electrophoresis^21^ was introduced to test the ability of the RAPTA complex, 4c, to destroy DNA. The results revealed that the total degradation of plasmid DNA (≈10 kb) was observed within 1.5 h (Fig. 2) on employing the selected compound 4c. Hence, this complex was found to have DNA degrading activity through the intercalation mode.

### BSA binding study

The binding interaction of the complexes 4c and 4d with bovine serum albumin (BSA) was investigated *via* the quenching of the emission intensity of the tryptophan unit in BSA upon the gradual treatment of these complexes and therefore the emission intensity of BSA was found to decrease progressively at 350 nm (Fig. S4†). The *K*_BSA_ of the complexes was obtained from Stern–Volmer plots by applying the well-known Stern–Volmer equation (Fig. S4†). On the other hand, the quenching constants (*k*_q_) of 6.3 × 10^14^ M^−1^ s^−1^ and 1.8 × 10^14^ M^−1^ s^−1^ arising from biomolecular interactions were evaluated from *K*_BSA_ and *τ*_o_ (1 × 10^−8^ s) for the complexes 4c and 4d, correspondingly (Table S3†). The analysis of the Scatchard plot gave the binding affinity (*K*) of these complexes where we observed the higher binding affinity (6.2 × 10^4^ M^−1^) of complex 4c as compared to 4d (5.25 × 10^4^) (Table S3†).

### Cytotoxic activity

**Fig. 3:**
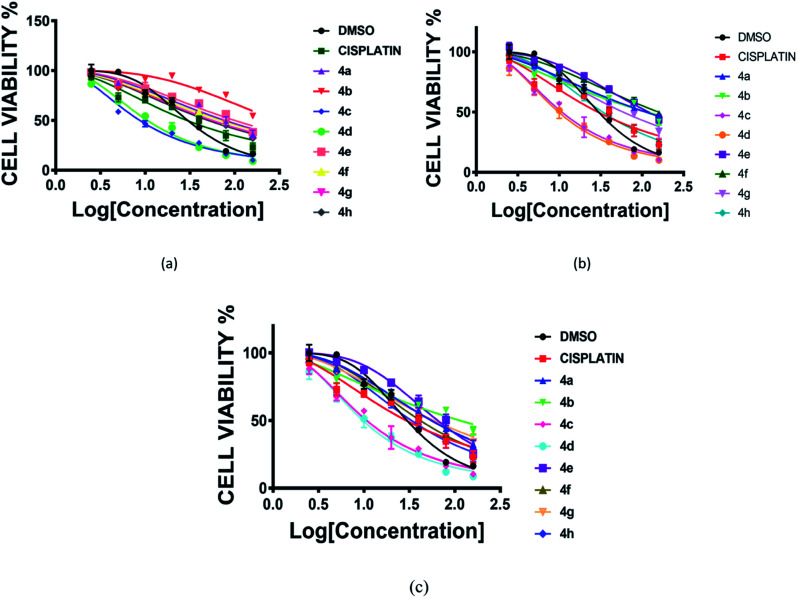
Cell viability (%) plot of complexes (4a–4h) and cisplatin against three human glioblastoma cancer cell lines [(a) LN229, (b) T98, (c) U87MG]. Incubation time 72 h.

The cytotoxic activity of prepared RAPTA complexes (4a–4h) was evaluated by following the standard procedure of the MTT experiment against a series of glioblastoma cells (LN 229, T98, U87MG) in triplicate. In order to accomplish this study, cells were maintained for 72 h with complexes of various concentrations ranging from 2–160 μM, taking cisplatin as a typical positive control.^22^ All these complexes exhibited significant potency against the aforementioned cell lines representing the IC_50_ value comparable to cisplatin. Complexes 4c and 4d displayed the best potency against all the brain cancer cells (Fig. 3 and Table 1).

**Table 1:** MTT cytotoxicity screening of 4a–4h against human cancer cell lines

Complex^a^	Cell line^b^ (IC_50_ μM)		
	LN229^c^	T98^c^	U87MG^c^
4a	1.80 ± 0.04	1.95 ± 0.04	1.69 ± 0.04
4b	2.19 ± 0.16	1.91 ± 0.08	1.91 ± 0.08
4c	0.88 ± 0.02	0.97 ± 0.02	0.97 ± 0.02
4d	0.96 ± 0.02	0.94 ± 0.02	0.93 ± 0.02
4e	1.89 ± 0.06	1.99 ± 0.04	1.75 ± 0.03
4f	1.79 ± 0.02	2.12 ± 0.03	1.57 ± 0.02
4g	1.69 ± 0.02	1.70 ± 0.03	1.70 ± 0.03
4h	1.64 ± 0.03	1.47 ± 0.03	1.46 ± 0.03
Cisplatin	1.4 ± 0.05	1.36 ± 0.05	1.37 ± 0.04

a 72 h incubation time.

b IC_50_ 50% of cells that endure cell death at that concentration.

c Human glioblastoma cancer cell lines.

### Cell cycle analysis

**Fig. 4:**
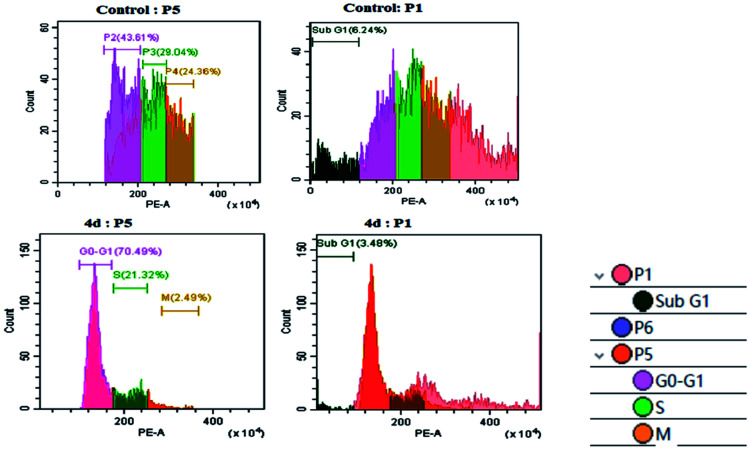
Cell cycle analysis with the control U87MG cells (above) and the treatment of 0.93 μM 4d (below).

As per the results of the MTT assay, there was an inhibition of cell proliferation that was further validated by determining the cellular damage, DNA damage and oxidative stress. Therefore, we went forward to investigate the DNA content by flow cytometry. As shown in Fig. 4, the U87MG cells exhibited a moderate P2 phase (∼43.61%), P3 phase (29.04%), P4 phase (24.36%) and low G0/G1, sub G1 (6.24%) in control conditions. The treatment of complex 4d with U87MG cells resulted in an increase in the G0/G1 phase to ∼70.49%, which indicated G0/G1 cell cycle arrest. We also observed a low S phase and M phase after the complex treatment (Fig. 4). Thus, we could successfully elucidate the role of the 4d complex in mediating a G0/G1 phase cell cycle arrest at the IC_50_ concentration of 0.93 μM.

### Neurosphere assay

**Fig. 5:**
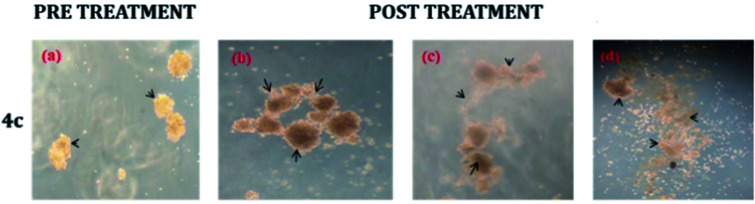
Neurosphere assay using different concentrations: (b) 20 μM, (c) 80 μM and (d) 160 μM of complex 4c against the T98G cell line; (a) pre-treatment (concentration of 4c = 0 μM).

The neurosphere assay is one of the best methods for analysing the stem cell capacity of isolated brain cells.^23^ The stem cell media was used to grow T98G cells through single-cell division within 3 days and subsequently the development of small-diameter “neurosphere-like” tumour spheres within 7 days. After 12–14 days of culture, a phase-contrast microscope was used to take the image of the tumour spheres. To promote differentiation, DMEM (containing 10% FBS) medium was added to the tumour spheres. Complete developmental neurotoxicity was observed with the complex 4c treatment (Fig. 5).

### Time-dependent reactive oxygen species (ROS) generation study

**Fig. 6:**
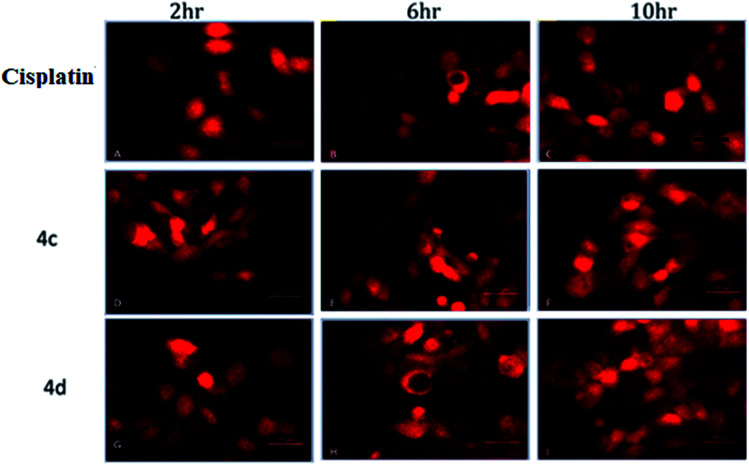
Assessment of the intracellular ROS study by DCFH-DA staining. (A–C) Cisplatin treated; (D–F) 4c treated; (G–I) 4d treated. Concentration 20 μM and incubation time 2–10 h.

**Fig. 7:**
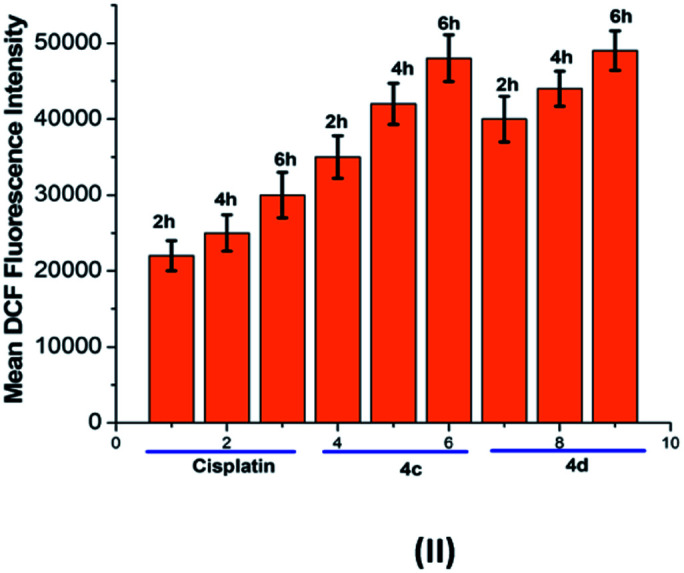
Graphical representation of the average DCF fluorescence intensity in the T98G cell line upon treatment with cisplatin and complexes 4c and 4d. Incubation time: 2 h, 6 h and 10 h.

The oxidative stress was measured by the level of intracellular ROS *via* 2′,7′-dichlorodihydrofluorescein diacetate (DCFH-DA) staining.^24^ It was observed that the vigorous diffusion of DCFH-DA into the cells and its deacetylation to a nonfluorescent compound by cellular esterases resulted in the formation of the fluorescent 2′,7′-dichlorofluorescein by intracellular ROS molecules. Herein, cells were treated with complexes 4c and 4d with a concentration of 20 μM for 2, 6 and 10 h. We observed a higher level of red fluorescence with the increase in the incubation time because of ROS generation (Fig. 6). The average DCF fluorescence intensity by complex 4c and 4d treatment indicated a gradual increase with respect to the incubation time (Fig. 7).

### Real-time reverse transcription (RT)-polymerase chain reaction (PCR)

It is known that the NF-κB pathway plays an important role in the modulation of inflammatory and immune responses.^25^ Under normal conditions, NF-κB is found in the cytoplasm as the dimer complex p65/p50 with the kappa B inhibitor forming the NF-κB-IκB complex which involves TNF-α. As expected, the treatment with TNF-α induces IκB protein phosphorylation and the NF-κB dimer p65/p50 is translocated into the nucleus. Inside the nucleus, the NF-κB dimer binds to its own DNA and the κB site, initiating the transcription of many genes associated with proliferation as well as the invasion of cells, anti-apoptosis along with drug resistivity (*e.g.*, Bcl-2 family, Bcl-XL, cyclin D1, c-myc, interleukin-1 and 6 (IL-6), cyclooxygenase-2 (COX-2), inducible NO-synthase (iNOS)). Complex 4c reduced TNF-α-induced NF-κB phosphorylation at 48 h. In summary, complex 4c inhibited the TNF-α-induced NF-κB phosphorylation in glioma cells and NF-κB could be validated as a targeted therapeutic molecule for tumour diseases (Fig. S5†).

### Immunoassay: VEGF Elisa test

**Fig. 8:**
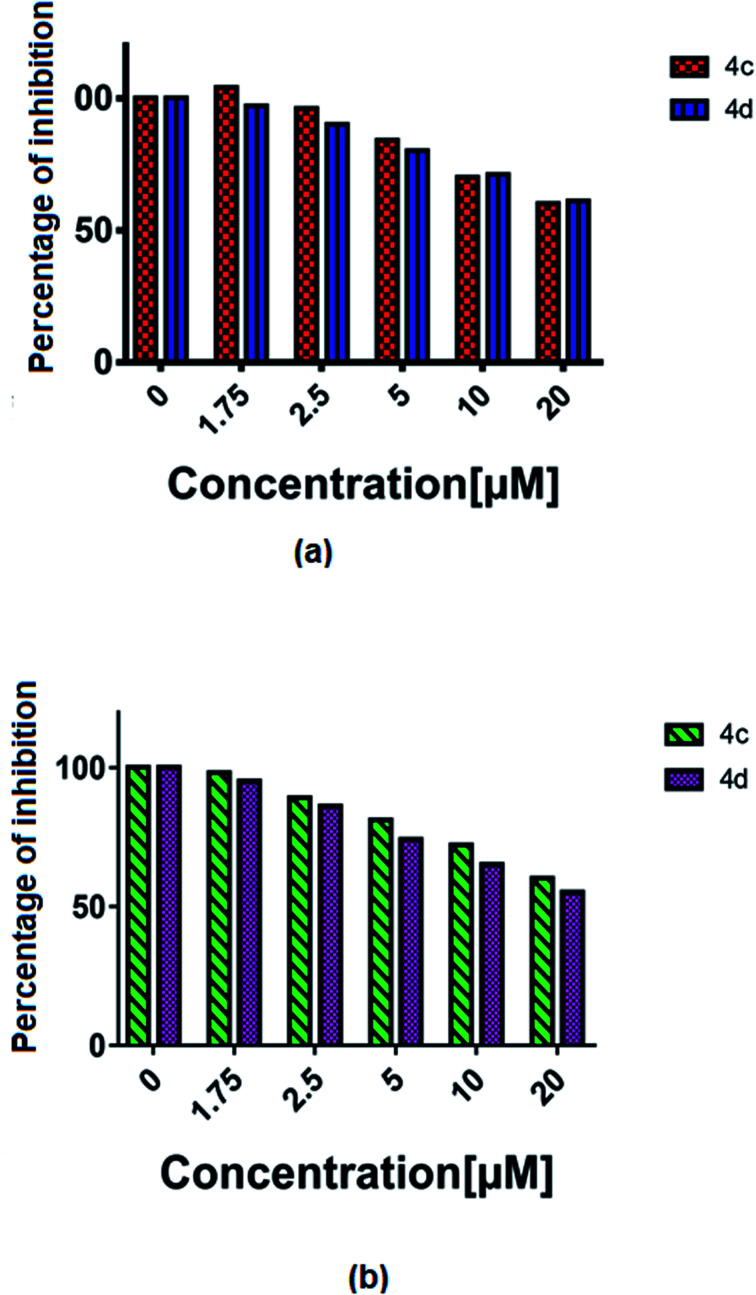
The % of VEGF inhibition with the treatment of 4c and 4d in the (a) T98G and (b) U87MG cell lines.

The vascular endothelial growth factor (VEGF), formerly known as the vascular permeability factor (VPF) is a signal protein produced by many cells, which stimulates blood vessel formation. In hypoxic conditions when blood circulation is insufficient in the cells, VEGF restores the oxygen supply to tissues.^26^ Proper blood supply is required for solid tumour proliferation beyond a limited size; therefore, cancers that can express VEGF are able to grow and metastasize. Two cancer cells, *i.e.* U87MG and T98G were incubated with increased concentrations (1 to 20 μM) of 4c and 4d and the % of inhibition of VEGF was estimated by an ELISA reader. A constant decrease in the VEGF concentration with the increase in complex concentration was observed (all *P* < 0.0001) (Fig. 8).

### *In vivo* study

**Fig. 9:**
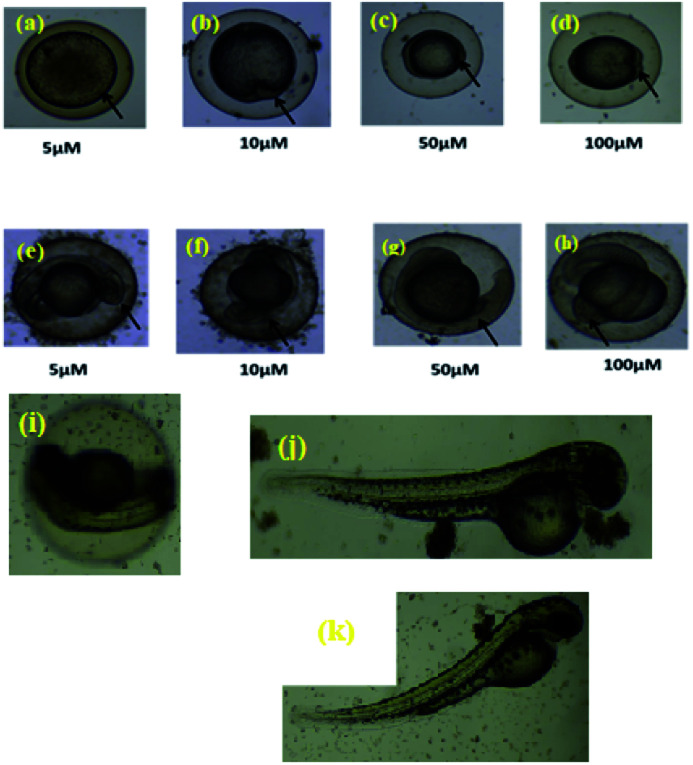
*In vivo* treatment of complex 4c in the zebrafish model ((a–d) 5 h, (e–h) 10 h, complex concentration = 5–100 μM); ((i–j) 48 h, complex concentration = 50 and 100 μM, (k) 72 h, complex concentration = 100 μM).

The zebrafish has become a widely used model organism in modern drug discovery because of its fecundity, its morphological and physiological similarity to mammals, the existence of many genomic tools and the ease with which large, phenotype-based screens can be performed.^27^ This study investigated the effects of exposure of vertebrate systems to the most potent complex 4c using the unique advantages of the embryonic zebrafish model. Screening level toxicological testing was accomplished to determine *in vivo* responses to, and biological consequences of RAPTA exposure. Microinjections of 4c dispersions were administered to embryonic zebrafish to test the effects of exposure *via* an injection route. To this end, complex 4c with various concentrations was intraperitoneally injected into the adult zebrafish and their mortality was followed (Fig. 9). Morphological deformities provoked by aquatic exposure to RAPTA complexes were mimicked by injection exposures for complex 4c. No significant morbidity or mortality was observed when the embryos were exposed for 5 h to 100 μM of complex 4c. It was also observed that complex 4c with 50 μM concentration did not show any toxicity for up to 72 h, indicating that RAPTA complexes are biocompatible for *in vivo* studies (Fig. 9).^28^

### *In vivo* biodistribution study^29,30^

**Fig. 10:**
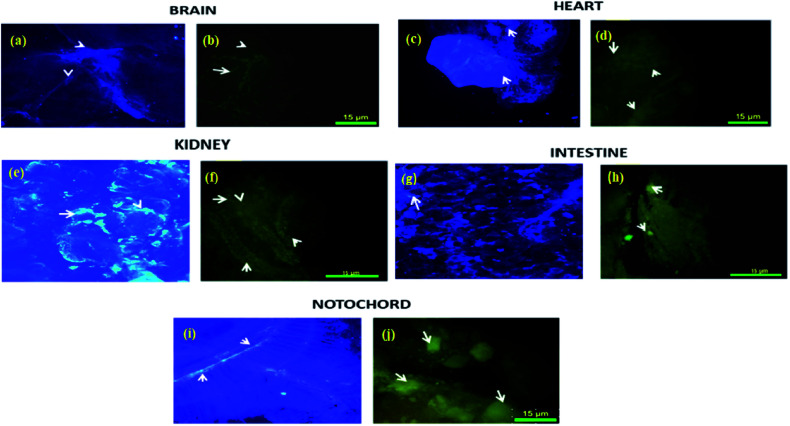
The *in vivo* biodistribution of complex 4c in the zebrafish model using Evans blue dye as a control. Evans blue treatment of the (a) brain, (c) heart, (e) kidney, (g) intestine and (i) notochord. Complex 4c treatment of the (b) brain, (d) heart, (f) kidney, (h) intestine and (i) notochord; incubation time: 15 min.

Biodistribution and sequestration studies of complex 4c were conducted *via* the caudal vein injection into the zebrafish. After 15 minutes (25 μg g^−1^) of exposure, the fish were sacrificed and different organs (brain, heart, kidney, intestine, notochord and digestive system) were dissected and the biodistribution of this complex into these organs was measured by fluorescence microscopy. A comparative study was performed with complex 4c and Evans blue dye and was analyzed by the confocal imaging of the tissue sections. From this experiment, it was observed that the sensitivity of zebrafish towards 4c was sequestered in the brain, heart, kidney, intestine, notochord and digestive system. As demonstrated in Fig. 10, the digestive system, kidney and notochord exhibited maximum sequestration after 15 minutes. The same trends were exhibited in the heart, with a broad distribution in the zebrafish model. In the brain, accumulation was significant after 15 minutes of incubation, thereby further confirming the therapeutic value of 4c. Furthermore, this fluorescence-based interaction could provide us with an idea of the uptake mechanism *via* crossing the blood–brain barrier.

## Experimental section

### Materials and methods

E. Merck supplied all of the organic solvents (India). Sigma Aldrich provided bipyridine derivatives, phenanthroline derivatives, 1,3,5-triaza-7-phosphaadamantane (PTA), and ruthenium^II^(dichloro)(*p*-cymene) dimer. Methanol was employed as the solvent system for thin-layer chromatography on pre-coated silica gel 60 F254 aluminium sheets (E. Merck, Germany). Sigma Aldrich Chemical Limited provided bovine serum albumin (BSA) and cell lines. On a 400 MHz Advanced Bruker DPX spectrometer, ^1^H NMR, ^13^C NMR, ^19^F NMR, and ^31^P NMR spectra were obtained using tetramethylsilane (TMS) as an internal standard. The chemical shifts (*δ*) were measured in parts per million (ppm). s, singlet; brs, broad singlet; d, doublet; dd, double doublet; t, triplet; m, multiplet are examples of abbreviations. On an Elchem Microprocessor-based DT equipment, the melting points of the complexes were determined. A TDS conductometer-307 was used to measure the conductivity of these complexes. On a Shimadzu Affinity FT-IR spectrometer, infrared spectra (IR) in the range of 4000–400 cm^−1^ were acquired. The mass spectra of the synthesised complexes were acquired using methanol as the solvent on a Shimadzu ESI-MS-4000 Mass Spectroscopic equipment with a 4000 triple quadrupole MS. A JASCO V-730 spectrophotometer with a 1 cm quartz cell was used to obtain UV-visible spectra. A Hitachi F7000 fluorescence spectrophotometer with a xenon lamp was used to record the fluorescence spectra. The MTT assay was performed using an Elisa reader with a 96-well plate. UPLC was used to assess the purity of such complexes. NCCS (Pune) provided the U87MG and LN229 human glioma cell lines, while Christian Medical College, Vellore provided the T98G cell line. These cell lines were grown in 5 percent CO_2_ in Dulbecco's Modified Eagle's Medium (DMEM, Gibco) with heat-inactivated foetal bovine serum (FBS, Himedia). When the cells reached 80% confluence, they were sent to the next step. Gibco provided the low glucose medium, DMEM/F12 medium, basic fibroblast growth factor (bFGF), epidermal growth factor (EGF), B27, DMSO, and trypsin. Sigma provided the thiazole salt (MTT). An Olympus fluorescence microscope (BX41) was utilised for the fluorescence imaging study.

#### Synthesis and characterisation of RAPTA complexes (4a–h)

Briefly, bipyrdine/phenanthroline ligands (2a–h) and [(η^6^-*p*-cymene)Ru^II^Cl(μ-Cl)]_2_ (1 : 0.5) were stirred for 2 h, followed by the addition of 1 equivalent of 1,3,5-triaza-7-phosphaadamantane (PTA) and the stirring was continued for another 2 h. The counter chloride ion was exchanged *via* treatment with ammonium hexafluorophosphate (NH_4_PF_6_) for another 1 h. The progress of these reactions was monitored by thin-layer chromatography (TLC) in methanol for the complexes 4a–h. A colour change was observed from deep yellow to dark orange. The crude RAPTA complexes (4a–h) were recrystallized with 10% methanol in a diethyl ether mixture following the vapour diffusion technique, and crystalline products were formed with great yields (93–95%).

##### [(η^6^-*p*-Cymene)Ru^II^(κ^2^-*N*,*N*-4,7dihydroxyphenanthroline)(PTA)]·2PF_6_ [4a]

Yield: 93%; yellow solid, Mp: 207–209 °C; *R*_f_ (20% EtOAc/methanol): 0.50; UPLC purity (99.9%, eluent: 50% H_2_O/ACN, *R*_t_: 0.435 min); IR (cm^−1^): *ν* 825, 875, 958, 1026, 1116, 1226, 1305, 1327, 1404, 1421, 1583, 2968, 3325, 3622; ^1^H NMR (DMSO-*d*_6_, 400 MHz): *δ* 0.88 (d, *J* = 6.68 Hz, 3H, cymene isopropyl-CH_3_), 2.36 (s, 3H, cymene isopropyl-CH_3_), 2.63–2.68 (m, 1H, cymene CH), 3.61 (m, 12H, PTA), 6.16 (d, *J* = 6.0 Hz, 2H, cymene ArH), 6.34 (d, *J* = 5.2 Hz, 2H, cymene ArH), 6.54 (brs, 2H, OH), 7.08 (s, 1H, ArH), 8.02 (s, 1H, ArH), 8.66 (d, *J* = 5.2 Hz, 2H, ArH); ^13^C NMR (DMSO-*d*_6_, 100 MHz): *δ* 18.7, 21.8, 30.8, 39.8, 53.2, 53.7, 70.6, 70.7, 83.8, 103.7, 104.1, 126.8, 127.9, 130.4, 139.2, 145.4, 148.9, 167.0; ^31^P NMR (DMSO-*d*_6_, 162 MHz): *δ* −32.0 (PTA), −131.0 to −157.4 (PF_6_); ^19^F NMR (DMSO-*d*_6_, 376 MHz): *δ* −69.0, −70.9; ESI-MS (MeOH): *m*/*z* for C_28_H_34_N_5_O_2_P_3_F_12_Ru: 302.7 [M-2PF_6_]^2+^.

##### [(η^6^-*p*-Cymene)Ru^II^(κ^2^-*N*,*N*-phenanthroline)(PTA)]·2PF_6_ [4b]

Yield: 95%; yellow solid, Mp: 207–209 °C; *R*_f_ (20% EtOAc/methanol): 0.50; UPLC purity (97.2%, eluent: 50% H_2_O/ACN, *R*_t_: 0.417 min); IR (cm^−1^): *ν* 823, 950, 1026, 1095, 1309, 1429, 1516; ^1^H NMR (DMSO-*d*_6_, 400 MHz): *δ* 0.82 (d, *J* = 6.84 Hz, 3H, cymene isopropyl-CH_3_), 2.13 (s, 3H, cymene isopropyl-CH_3_), 2.53–2.59 (m, 1H, cymene CH), 3.84–4.65 (m, 12H, PTA), 6.16 (d, *J* = 6.0 Hz, 2H, cymene ArH), 5.99 (d, *J* = 6.12 Hz, 2H, cymene ArH), 6.23 (d, *J* = 6.12 Hz, 2H, cymene ArH), 8.11 (m, 4H, ArH), 8.20 (s, 1H, ArH), 8.84 (d, *J* = 8.2 Hz, 2H, ArH), 9.81 (d, *J* = 5.2 Hz, 2H); ^13^C NMR (DMSO-*d*_6_, 100 MHz): *δ* 18.7, 21.8, 30.8, 38.5, 53.2, 53.7, 70.6, 70.7, 83.3, 86.4, 103.7, 104.1, 126.8, 127.9, 130.4, 139.2, 145,4, 156.0; ^31^P NMR (DMSO-*d*_6_, 162 MHz): *δ* −31.0 (PTA), −131.1 to −155.4 (PF_6_); ^19^F NMR (DMSO-*d*_6_, 376 MHz): *δ* −69.0, −70.9; ESI-MS (MeOH): *m*/*z* for C_28_H_34_N_5_P_3_F_12_Ru: 286.6 [M-2PF_6_]^2+^.

##### [(η^6^-*p*-Cymene)Ru^II^(κ^2^-*N*,*N*-4,7dimethyl phenanthroline)(PTA)]·2PF_6_ [4c]

Yield: 95%; yellow solid, Mp: 202–204 °C; *R*_f_ (20% EtOAc/methanol): 0.48; UPLC purity (95.4%, eluent: 50% H_2_O/ACN, *R*_t_: 0.422 min); IR (cm^−1^): *ν* 831, 950, 979, 1026, 1095, 1197, 1246, 1307, 1409, 1519, 1658, 2970; ^1^H NMR (DMSO-*d*_6_, 400 MHz): *δ* 0.86 (d, *J* = 7.2 Hz, 3H, cymene isopropyl-CH_3_), 2.16 (s, 3H, cymene isopropyl-CH_3_), 2.56–2.60 (m, 1H, cymene CH), 2.95 (s, 6H, CH_3_), 3.95–3.97 (m, 6H, PTA), 4.33 (d, *J* = 12.4 Hz, 3H, PTA), 4.43 (d, *J* = 12.4 Hz, 3H, PTA), 6.03 (d, *J* = 6.4 Hz, 2H, cymene ArH), 6.27 (d, *J* = 6.0 Hz, 2H, cymene ArH), 7.99 (d, *J* = 5.2 Hz, 2H, ArH), 8.34 (s, 2H, ArH), 9.73 (d, *J* = 5.6 Hz, 2H); ^13^C NMR (DMSO-*d*_6_, 100 MHz): *δ* 18.7, 22.1, 30.8, 46.3, 46.6, 54.2, 71.0, 83.9, 86.3, 103.2, 124.5, 127.2, 129.8, 132.3, 145.3, 149.6, 155.7; ^31^P NMR (DMSO-*d*_6_, 162 MHz): *δ* −32.0 (PTA), −131.1 to −155.4 (PF_6_); ^19^F NMR (DMSO-*d*_6_, 376 MHz): *δ* −69.0, −71.0; ESI-MS (MeOH): *m*/*z* for C_30_H_38_N_5_P_3_F_12_Ru: 300.1 [M-2PF_6_]^2+^.

##### [(η^6^-*p*-Cymene)Ru^II^(κ^2^-*N*,*N*-4,7diphenyl phenanthroline)(PTA)]·2PF_6_ [4d]

Yield: 93%; orange solid, Mp: 200–202 °C; *R*_f_ (20% EtOAc/methanol): 0.49; UPLC purity (96.2%, eluent: 50% H_2_O/ACN, *R*_t_: 0.418 min); IR (cm^−1^): *ν* 831, 950, 979, 1026, 1095, 1197, 1246, 1307, 1409, 1519, 1658, 2970; ^1^H NMR (DMSO-*d*_6_, 400 MHz): *δ* 0.93 (d, *J* = 6.8 Hz, 6H, cymene isopropyl-CH_3_), 2.10 (s, 3H, cymene isopropyl-CH_3_), 2.59–2.66 (m, 1H, cymene CH), 3.66–4.84 (m, 12H, PTA), 4.43 (d, *J* = 12.4 Hz, 3H, PTA), 6.08 (d, *J* = 6.4 Hz, 2H, cymene ArH), 6.30 (d, *J* = 6.4 Hz, 2H, cymene ArH), 7.56–7.66 (m, 10H, ArH), 8.04 (s, 4H, ArH), 9.91 (d, *J* = 5.6 Hz, 2H); ^13^C NMR (DMSO-*d*_6_, 100 MHz): *δ* 18.7, 22.1, 30.8, 46.3, 46.6, 54.2, 71.0, 84.0, 86.3, 103.2, 104.1, 120.1, 124.5, 127.2, 129.8, 132.3, 140.4, 145.3, 149.6, 155.8; ^31^P NMR (DMSO-*d*_6_, 162 MHz): *δ* −32.4 (PTA), −131.1 to −157.4 (PF_6_); ^19^F NMR (DMSO-*d*_6_, 376 MHz): *δ* −69.0, −71.0; ESI-MS (MeOH): *m*/*z* for C_40_H_44_N_5_P_3_F_12_Ru: 363.4 [M-2PF_6_]^2+^.

##### [(η^6^-*p*-Cymene)Ru^II^(κ^2^-*N*,*N*-bipyridine)(PTA)]·2PF_6_ [4e]

Yield: 94%; yellow solid, Mp: 205–207 °C; *R*_f_ (20% EtOAc/methanol): 0.46; UPLC purity (97.2%, eluent: 50% H_2_O/ACN, *R*_t_: 0.430 min); IR (cm^−1^): *ν* 829, 877, 977, 1026, 1168, 1230, 1307, 1446, 1519, 1577, 2966; ^1^H NMR (DMSO-*d*_6_, 400 MHz): *δ* 0.78 (d, *J* = 6.8 Hz, 3H, cymene isopropyl-CH_3_), 0.86 (d, *J* = 6.8 Hz, 3H, cymene isopropyl-CH_3_), 2.12 (s, 3H, cymene isopropyl-CH_3_), 3.14 (m, 1H, cymene CH), 3.89–3.97 (m, 12H, PTA), 5.80 (d, *J* = 6.0 Hz, 2H, cymene ArH), 5.89 (d, *J* = 6.4 Hz, 2H, cymene ArH), 6.06 (d, *J* = 6.4 Hz, 2H, cymene ArH), 6.13 (d, *J* = 6.0 Hz, 2H, cymene ArH), 7.70 (d, *J* = 6.0 Hz, 2H, ArH), 7.89 (d, *J* = 5.6 Hz, 1H, ArH), 8.17 (t, *J* = 8.0 Hz, 1H, ArH), 8.27 (s, 1H, ArH), 8.42 (d, *J* = 8.4 Hz, 1H, ArH), 9.37 (d, *J* = 5.6 Hz, 1H, ArH); ^13^C NMR (DMSO-*d*_6_, 100 MHz): *δ* 18.6, 21.8, 30.8, 70.5, 71.2, 83.9, 87.2, 124.0, 128.0, 130.0, 140.5, 155.8; ^31^P NMR (DMSO-*d*_6_, 162 MHz): *δ* −32.0 (PTA), −131.1 to −157.4 (PF_6_); ^19^F NMR (DMSO-*d*_6_, 376 MHz): *δ* −69.0, −71.0; ESI-MS (MeOH): *m*/*z* for C_26_H_34_N_5_P_3_F_12_Ru: 274.6 [M-2PF_6_]^2+^.

##### [(η^6^-*p*-Cymene)Ru^II^(κ^2^-*N*,*N*-4,4′ dicarboxylic acid bipyridine)(PTA)]·2PF_6_ [4f]

Yield: 95%; yellow solid, Mp: 204–206 °C; *R*_f_ (20% EtOAc/methanol): 0.44; UPLC purity (96.5%, eluent: 50% H_2_O/ACN, *R*_t_: 0.436 min); IR (cm^−1^): *ν* 831, 914, 1012, 1068, 1141, 1238, 1265, 1288, 1365, 1406, 1458, 1552, 1602, 1710, 2411, 2974; ^1^H NMR (DMSO-*d*_6_, 400 MHz): *δ* 1.14 (d, *J* = 6.8 Hz, 3H, cymene isopropyl-CH_3_), 2.22 (s, 3H, cymene isopropyl-CH_3_), 2.55–2.58 (m, 1H, cymene CH), 4.15–4.30 (m, 12H, PTA), 4.80 (d, *J* = 6.0 Hz, 2H, cymene ArH), 5.97 (d, *J* = 6.4 Hz, 1H, cymene ArH), 6.23 (d, *J* = 6.4 Hz, 1H, cymene ArH), 7.88–8.21 (m, 4H, ArH), 8.81 (s, 1H), 9.12 (d, *J* = 6.0 Hz, 1H, ArH), 9.54 (s, 1H, ArH), 9.61 (s, 1H); ^13^C NMR (DMSO-*d*_6_, 100 MHz): *δ* 18.7, 21.9, 30.7, 38.7, 40.0, 82.6, 85.5, 110.9, 120.0, 122.7, 126.5, 147.2, 155.9, 164.8; ^31^P NMR (DMSO-*d*_6_, 162 MHz): *δ* −59.0 (PTA), −131.1 to −157.4 (PF_6_); ^19^F NMR (DMSO-*d*_6_, 376 MHz): *δ* −69.0, −71.0; ESI-MS (MeOH): *m*/*z* for C_28_H_34_N_5_O_4_P_3_F_12_Ru: 318.2 [M-2PF_6_]^2+^.

##### [(η^6^-*p*-Cymene)Ru^II^(κ^2^-*N*,*N*-4,4′ dimethylbipyridine)(PTA)]·2PF_6_ [4g]

Yield: 94%; yellow solid, Mp: 206–207 °C; *R*_f_ (20% EtOAc/methanol): 0.45; UPLC purity (98.2%, eluent: 50% H_2_O/ACN, *R*_t_: 0.432 min); IR (cm^−1^): *ν* 819, 950, 1024, 1166, 1294, 1423, 1633, 3321; ^1^H NMR (DMSO-*d*_6_, 400 MHz): *δ* 0.86 (d, *J* = 8.8 Hz, 6H, cymene isopropyl-CH_3_), 2.12 (s, 3H, cymene isopropyl-CH_3_), 2.54–2.58 (m, 1H, cymene CH), 2.94 (s, 3H, Me), 3.77–4.45 (m, 12H, PTA), 6.06 (t, *J* = 6.0 Hz, 2H, cymene ArH), 6.27 (brs, 2H, cymene ArH), 8.0 (d, *J* = 5.6 Hz, 1H, ArH), 8.11–8.13 (m, 1H, ArH), 8.28 (d, *J* = 9.2 Hz, 1H, ArH), 8.33 (d, *J* = 8.4 Hz, 1H, ArH), 8.90 (d, *J* = 8.4 Hz, 1H, ArH), 9.74 (d, *J* = 5.6 Hz, 1H, ArH), 9.88 (d, *J* = 5.2 Hz, 1H, ArH); ^13^C NMR (DMSO-*d*_6_, 100 MHz): *δ* 18.8, 20.7, 21.9, 30.5, 46.7, 52.9, 53.6, 70.3, 71.4, 83.8, 86.5, 127.3, 128.2, 140.3, 154.8, 157.7; ^31^P NMR (DMSO-*d*_6_, 162 MHz): *δ* −31.7 (PTA), −131.1 to −157.4 (PF_6_);^19^F NMR (DMSO-*d*_6_, 376 MHz): *δ* −69.0, −70.9; ESI-MS (MeOH): *m*/*z* for C_27_H_36_N_5_P_3_F_12_Ru: 281.3 [M-2PF_6_]^2+^.

##### [(η^6^-*p*-Cymene)Ru^II^(κ^2^-*N*,*N*-4,4′ dinonylbipyridine)(PTA)]·2PF_6_ [4h]

Yield: 94%; yellow solid, Mp: 206–207 °C; *R*_f_ (20% EtOAc/methanol): 0.45; UPLC purity (98.2%, eluent: 50% H_2_O/ACN, *R*_t_: 0.432 min); IR (cm^−1^): *ν* 819, 950, 1024, 1166, 1294, 1423, 1633, 3321; ^1^H NMR (DMSO-*d*_6_, 400 MHz): *δ* 0.81–0.88 (m, 6H, cymene isopropyl-CH_3_), 0.90 (m, 6H, alkyl group), 1.21–1.29 (m, 24H, alkyl group), 1.66 (brs, 4H, alkyl group), 2.14 (s, 3H, cymene isopropyl-CH_3_), 2.51–2.55 (m, 1H, cymene CH), 2.77–2.81 (m, 4H, alkyl group), 3.68–4.63 (m, 12H, PTA), 5.92 (d, *J* = 6.0 Hz, 2H, cymene ArH), 6.16 (d, *J* = 6.0 Hz, 2H, cymene ArH), 7.60 (d, *J* = 5.6 Hz, 2H, ArH), 8.51 (s, 2H, ArH), 9.34 (d, *J* = 6.0 Hz, 1H, ArH); ^13^C NMR (DMSO-*d*_6_, 100 MHz): *δ* 15.2, 18.7, 21.8, 22.9, 28.0, 29.7, 30.8, 32.2, 33.4, 39.8, 53.2, 53.7, 70.6, 70.7, 83.8, 86.4, 123.6, 125.8, 130.4, 145.4, 147.4, 150.3, 151.1; ^31^P NMR (DMSO-*d*_6_, 162 MHz): *δ* −31.8 (PTA), −131.1 to −157.4 (PF_6_);^19^F NMR (DMSO-*d*_6_, 376 MHz): *δ* −69.0, −71.0; ESI-MS (MeOH): *m*/*z* for C_44_H_70_N_5_P_3_F_12_Ru: 400.5 [M-2PF_6_]^2+^.

#### Biological study

##### *In vitro* cytotoxic activities (MTT assay)^22^

RAPTA complexes were dissolved in 1% DMSO, and 0.9% NaCl aqueous solution was used to dissolve cisplatin. A cell suspension was prepared and the number of cells taken was 2.5 × 10^4^ mL^−1^. The cells were seeded in 96-well plates (200 μL per well) and allowed 80% confluence prior to treatment. The cells were formerly treated with various concentrations (2.5–160 μM) of complex 4a–h while the control group was treated with PBS for 24 h. After the 72 hours of incubation, 0.5% MTT solution was added to each well (20 μL) and further incubated for 4 hours at room temperature. The formazan crystals that were formed were dissolved in 150 μL DMSO and then the OD was measured by a microplate reader at 540 nm. The % growth inhibition of the control and treated cells were calculated and represented as the growth inhibition percentage as explained below.% growth inhibition = 100 − [(AD × 100)/AB]where AD is the absorbance measured in wells containing the treated and control samples and AB is the measured absorbance for the wells with media and a vehicle that served as the blank. IC_50_ was calculated using Graphpad Prism (version 6.0) where % cell viability was plotted against drug concentration.

##### Neurosphere assay^19,23^

Serum-free DMEM/F-12 (1 : 1), B27 (1 : 50), EGF (20 μg L^−1^), bFGF (20 μg L^−1^), antibiotic (Gibco) were added to stem cell media. The cells were grown in a tissue culture incubator with 5% CO_2_ at room temperature. Serum-free media were changed twice a week.

##### DCFDA staining for the assessment of reactive oxygen species (ROS)^24^

Brain cancer cells were stained with 2′,7′-dichlorofluorescein diacetate (DCFDA) to measure the reactive oxygen species (ROS) and subsequent oxidative stress generated from RAPTA complexes. They were restained by the red fluorescence intensity generated from oxidized DCFDA. The cancer cells were cultured in a 24-well plate till 70% confluency and treatment was carried out with the aforesaid complex for 24 hours. The ROS generation mediated by the complex was evaluated using DCFDA-2′,7′-dichlorofluorescein diacetate (25 μM) staining for 30 min. The cells were washed with PBS, followed by fresh media replenishment. The images were captured under the EVOS M5000 fluorescent live cell imager (Thermo Fisher Scientific, USA).

##### Real-time reverse transcription (RT)-polymerase chain reaction (PCR)^25^

Cultured cells U87MG and T98G were treated with various concentrations of 4c (20–160 μM) for 48 h. Total RNA was extracted and complementary DNA (cDNA) was transcribed using Qiazol reagent and Thermo Scientific Revert Aid First Strand cDNA Synthesis Kit. Real-time PCR was performed using a Rotor gene Q Real-Time PCR System and Master SYBR Green was provided with the kit. Primers for both sense and antisense for 9 mouse gene and GAPDH mRNA were purchased from Eurofins and from Scientific Revert Aid First Strand, respectively, and these were used for this study. The amplified products were analyzed by a melting curve analysis.

##### Immunoassay: VEGF Elisa test^26^

The capture antibody was diluted to the working concentration in PBS without the carrier protein for coating the 96-well plate. The plate was sealed and incubated overnight at room temperature. It was then aspirated and washed three times with wash buffer and blocked by adding 300 μL of reagent diluent to each well and then incubating at room temperature for a minimum of 1 h. This aspiration was repeated again and then the plate was ready for sample addition.

##### Assay procedure

The cells (U87MG and T98G) were treated for 48 h and the supernatants were collected; 100 μL of these samples were added per well and incubated for 2 h at room temperature. Then, 100 μL of the antibody was added to each well and incubated for 2 h at room temperature, followed by washing. Next, 100 μL of streptavidin–HRP was added to each well. The whole system was incubated for another 20 min at room temperature in dark conditions and the aspiration was repeated. Then, 100 μL of substrate solution was added to each well and incubated for 20 min at room temperature in dark conditions. Stop solution (50 μL) was added to each well and mixed thoroughly. The optical density of each well was measured immediately by an Elisa reader (*λ*_exc_ = 450 nm).

##### Zebrafish model^27,28^

Zebrafish were purchased from Zebrafish CRO, Pentagrit Discovery, Chennai 600100. All the procedures were conducted with reference to the guidelines of OECD 203 (OECD 203, 1992).^29^ The local wild-type zebrafish strain weighing approximately 500–600 mg (2–3 months old) were maintained under standard laboratory conditions at 28 °C under a 14 : 10 h light/dark cycle with the conductivity of the water being 350 μS, maintained at pH 7.2–7.4. All the animal experiments including surgical procedures were conducted according to the procedures of the Committee for the Purpose of Control and Supervision of Experiments on Animals (CPCSEA), Govt. of India, as instructed by Institutional Animal ethical guidelines (VIT/IAEC/2019/16/Aug10/15), VIT University.^29^ All animal procedures were performed in accordance with the Guidelines for Care and Use of Laboratory Animals of “VIT” University and approved by the Animal Ethics Committee of “VIT”.

##### *In vivo* toxicity study

Freshly laid eggs [<1 h post-fertilisation (hpf)] were transferred to 50 ml crystallising dishes filled with the respective test solutions. After the successful control of fertilisation, the eggs were separately transferred to 24-well plates with 2 ml of test solution per well. All test vessels were pre-incubated (saturated) with the test solutions for at least 24 h. The well plates were then sealed with self-adhesive foil (SealPlate™ by EXCEL Scientific, Dunn, Asbach, Germany) and placed in an incubator at 26.0 ± 1.0 °C under a 10/14 h dark/light regime. The test medium was rehabilitated each day and all developmental alterations of the embryos were documented at 5–72 hpf. The embryos were analysed under an Olympus CKX41 inverted microscope (Olympus, Hamburg, Germany).

##### *In vivo* biodistribution study^30,31^

Zebrafish were anesthetized with 2-phenoxyethanol and injected with complex 4c*via* intraperitoneal injection. After injection at different times, the fish were sacrificed and the organs were collected and fixed with 4% paraformaldehyde and 4% glutaraldehyde, followed by dehydration with the gradient increase of ethanol (75%, 95%, 100%) for 20 min each, further with xylene for 1 h and then fixed in Paraplast. Blocks were stored at −10 °C for 12 h before proceeding to sectioning. Here, 10 μm thicknesses of sections were cut and the sections were collected on PLL-coated glass plates. The sections were washed with xylene to remove the excess Paraplast. After drying, the sections were fixed with mounting media. Sequestration of complex 4c in different organs was investigated by confocal fluorescence microscopy using a CLSM (Zeiss LSM 710) microscope. The excitation wavelength was fixed at 450 nm and the detection wavelength was 510 nm and 25× objective systems were used to image the digestive system sections.

## Conclusion

In summary, we have been able to assess the anticancer propensity of the series of synthesized Ru(ii)–arene PTA complexes (4a–h). In light of experimental evidence, it is comprehensible that complex 4c is superior to all other complexes with respect to displaying remarkable fluorescence properties with the highest quantum yield, good DNA binding ability, BSA binding affinity, along with significant DNA cleavage activity. Interestingly, a closer look at the cytotoxic profile of all these complexes revealed that complex 4d is the most potent towards T98 and U87MG and complex 4c is the most potent against the LN229 cancer cell line among all the complexes. The antiproliferative activity of these complexes was demonstrated by DNA cleavage, oxidative stress, neurotoxic behaviour and the VEGF downregulation in hypoxic conditions. We successfully elucidated the role of complex 4d in mediating a G0/G1 phase cell cycle arrest at the IC_50_ concentration. Real-time reverse transcription (RT)-polymerase chain reaction (PCR) studies revealed that complex 4c inhibited the TNF-α-induced NF-κB phosphorylation in glioma cells. It was also observed that complex 4c with 50 μM concentration does not show any toxicity for up to 72 h, which indicates that RAPTA complexes are biocompatible for *in vivo* studies. *In vivo* biodistribution studies showed the selective accumulation of ruthenium complexes *via* crossing the blood–brain barrier in glioblastoma cells. It can be concluded that all the complexes will be very efficient as anticancer complexes in the impending days.

## Conflicts of interest

“There are no conflicts to declare”.

## Supplementary Material

RA-012-D2RA02677E-s001
