# Supplementary material for: Ru(ii)arene(N^N bpy/phen)-based RAPTA complexes for in vitro anti-tumour activity in human glioblastoma cancer cell lines and in vivo toxicity studies in a zebrafish model
Source: RSC Adv. 2022 Jun 29;12(29):18911–22. doi: 10.1039/d2ra02677e (PMC9241057; doi:10.1039/d2ra02677e)
Supplement: RA-012-D2RA02677E-s001 [file RA-012-D2RA02677E-s001.pdf]

Electronic Supplementary Material (ESI) for RSC Advances.
This journal is © The Royal Society of Chemistry 2022

Supporting Information

Ru(II)arene(N^N bpy/phen) based RAPTA complexes for *in vitro* anti-tumor activity in human glioblastoma cancer cell lines and *in vivo* toxicity study in zebrafish model†

Anuja P K, Binoy Kar, Nilmadhab Roy, Priyankar Paira*

Department of Chemistry, School of advanced sciences, Vellore Institute of Technology Vellore-632014, Tamilnadu, India, E-mail: priyankar.paira@vit.ac.in

Fig.S1-S5,
Table S1-S3 ………………………………………………… 2 – 6
^{1}H, ^{13}C, ^{19}F, ^{31}P NMR Spectra …………………………… 7 - 20
FT-IR Spectra ………………………………………………… 21 - 23
HLPC ……………………………………………………………… 24 - 27
ESI-MS …………………………………………………………… 28 - 31
Experimental Section …………………………………… 32 - 35
References ………………………………………………………… 35 - 36

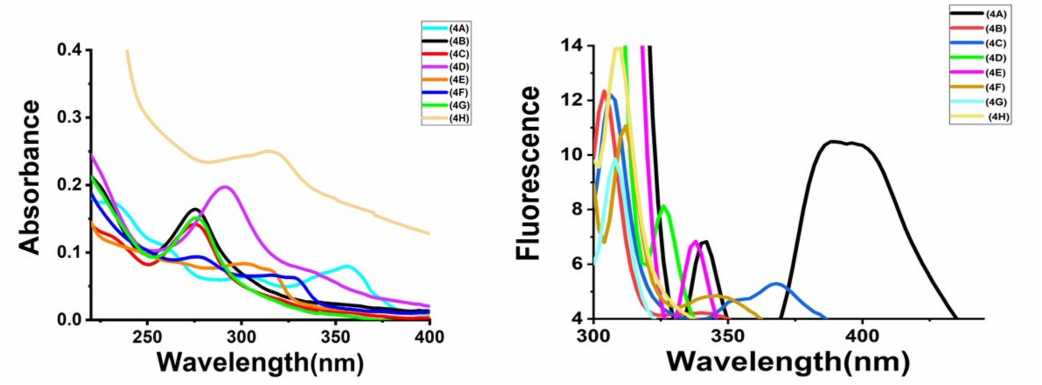

(a) (b)

Fig. S1 (a) Absorption and (b) Emission Spectral Responses of Ru(II)-arene PTA Complexes (**4a-h**) in DMSO-water (1:9, v/v) media

Table S1 Physicochemical characterization of RAPTA complexes (**4a-4h**)

Samples	stokes shift	OD[a]	ε (M^{-1} cm^{-1})[b]	(φ_f)[c]	solubility (M)[d]	log P[e]	Conductivity (Λ_M) (μS/cm)[f]	
							DMSO	10% aq DMSO
**4a**	44	0.03	3000	0.014	0.021	0.49±0.03	72	74
**4b**	42	0.04	4000	0.002	0.007	1.2±0.07	74	78
**4c**	41	0.07	7000	0.10	0.018	0.30±0.04	76	78
**4d**	74	0.08	8000	0.010	0.018	0.55±0.06	74	77
**4e**	65	0.04	4000	0.033	0.006	1.4±0.08	75	76
**4f**	63	0.04	4000	0.081	0.017	0.33±0.03	75	77
**4g**	40	0.1	10000	0.003	0.024	0.48±0.05	72	74
**4h**	38	0.02	2000	0.008	0.008	1.0±0.06	71	77
**Qninine Sulphate**	102	0.064	6400	0.546	-	-	-	-

[a]optical density, [b]extinction coefficient, [c]quantum yield, [d]DMSO-10% DMEM medium (1:99 v/v, comparable to cell media), [e]Partition Coefficients in n-Octanol/Water , [f]conductance in DMSO and 10% DMSO (3 x 10^{-5} M)

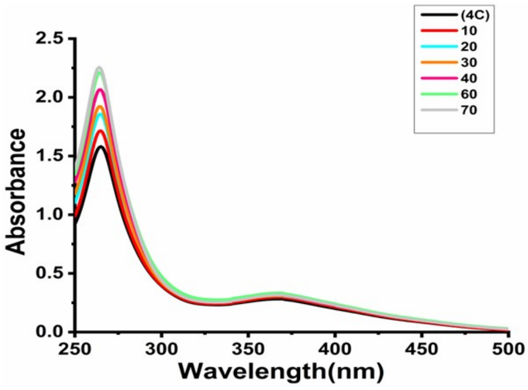

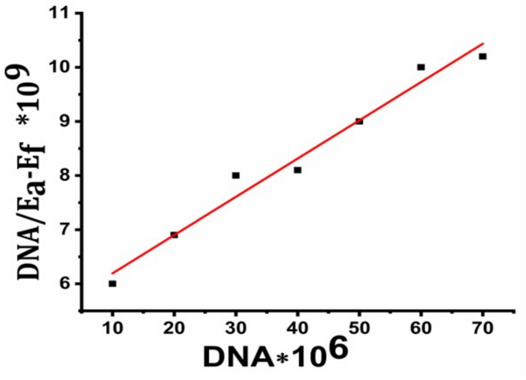

(a) (b)

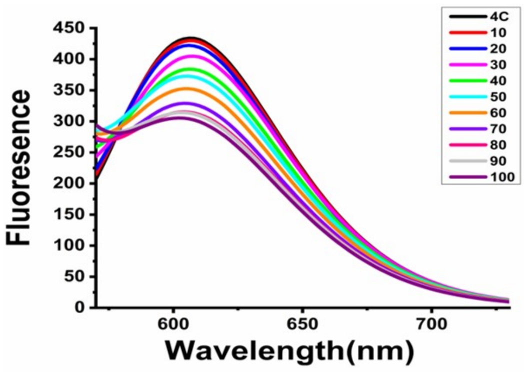

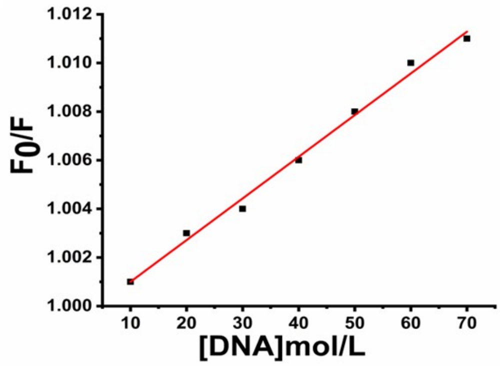

(c) (d)

Fig. S2 (a) and (b) UV-Visible spectral responses of RAPTA complex, **4c** ($1x10^{-5}$ M) in 5 mM Tris-HCl-NaCl (pH, 7.2) with incremental accumulation of Ct-DNA ($5x10^{-5}$ M); **(c)** and (d) Responses of fluorescence from EtBr bound DNA in the occurrence of complex **4c** (pH 7.2)

Table S2 Binding parameters for interaction of complex **4c** with Ct-DNA

Complex	Change in absorbance	Δε (%)[a]	K_b ($\times 10^5$ M^{-1})[b]	K_{sv} ($\times 10^4$ M^{-1})[c]	K_{app} ($\times 10^6$ M^{-1})[d]
**4c**	Hyperchromism	50	1.38±0.28	0.018	1.38

[a]% of hyperchromism/hypochromism, [b]K_b, intrinsic DNA binding constant from UV–visible absorption titration, [c]K_{sv}, Stern-Volmer quenching constant, [d]K_{app}, apparent DNA binding constant from competitive displacement from fluorescence spectroscopy.

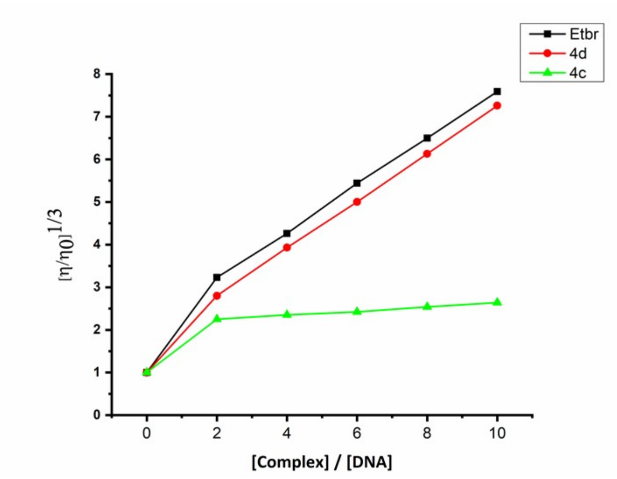

Fig. S3 Effect of Increasing Amounts of complex **4c** and **4d** on the Viscosity of Ct-DNA at 298 K ([EtBr] = 1×10^{-6} M, [DNA] = 1×10^{-6} M, [complex] = 1×10^{-3} M)

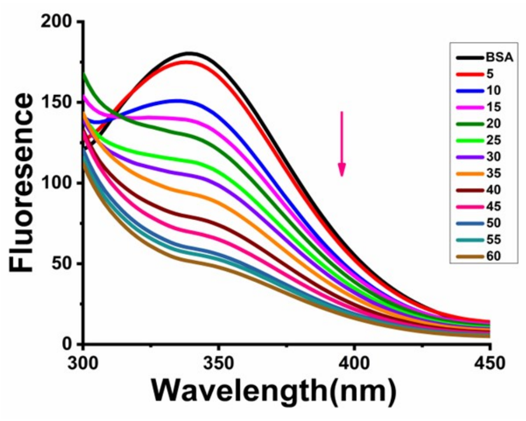

(a)

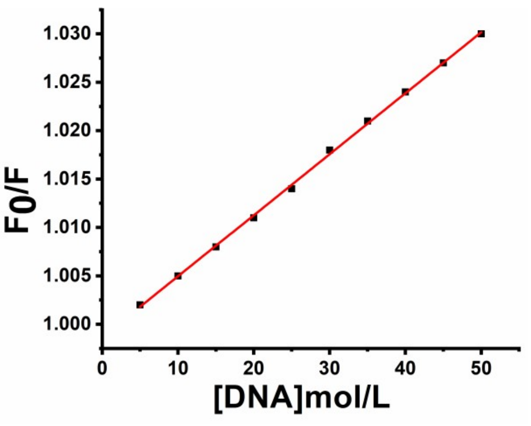

(b)

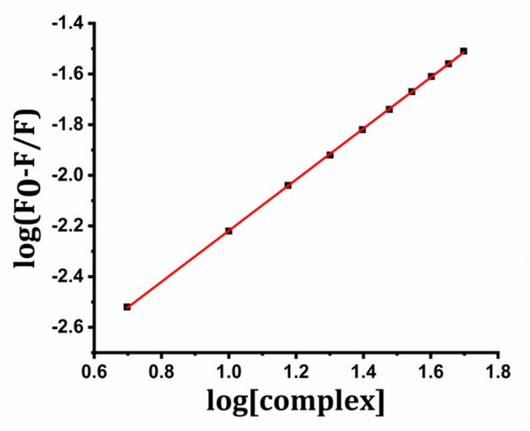

(c)

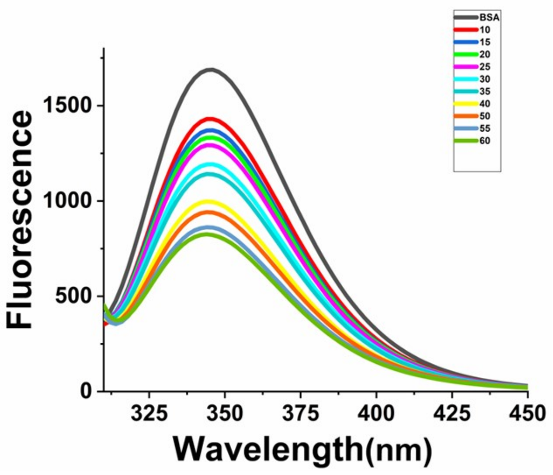

(d)

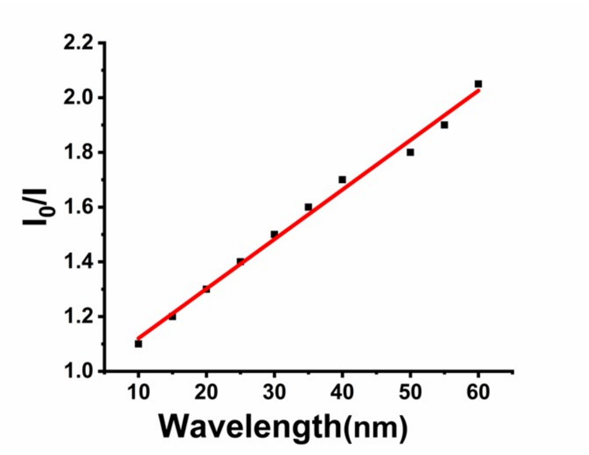

(e)

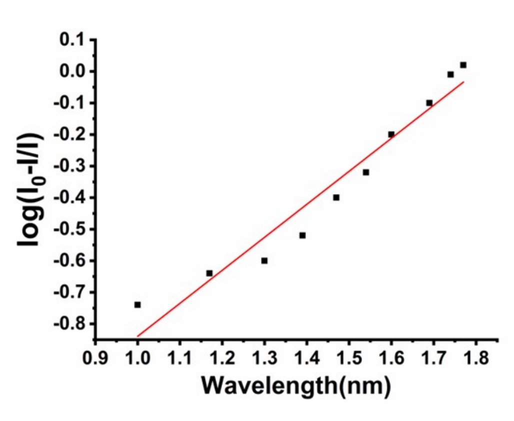

(f)

Fig. S4 Fluorescence Quenching of BSA on Addition of complex **4c** and **4d** in 5 mM TrisHCl/NaCl Buffer (pH 7.2)

Table S3. Binding Parameters of Ligand and Ru(II) Complexes with BSA

**Complex**	K_{BSA} (M^{-1})^{a}	k_q (M^{-1} s^{-1})^{b}	K (M^{-1})^{c}	n^{d}
**4c**	6.3x10^{6}	6.3x10^{14}	6.02x10^{4}	1
**4d**	1.8x10^{6}	1.8x10^{14}	5.25x10^{4}	1

$^{a}K_{BSA}$, Stern Volmer quenching constant; $^{b}K_q$, quenching rate constant (BSA); ^{c}K, binding constant with BSA; ^{d}n, number of binding sites (BSA). Concentrations of complexes = 0–60 µM in distilled water, BSA concentration was fixed at 5µM.

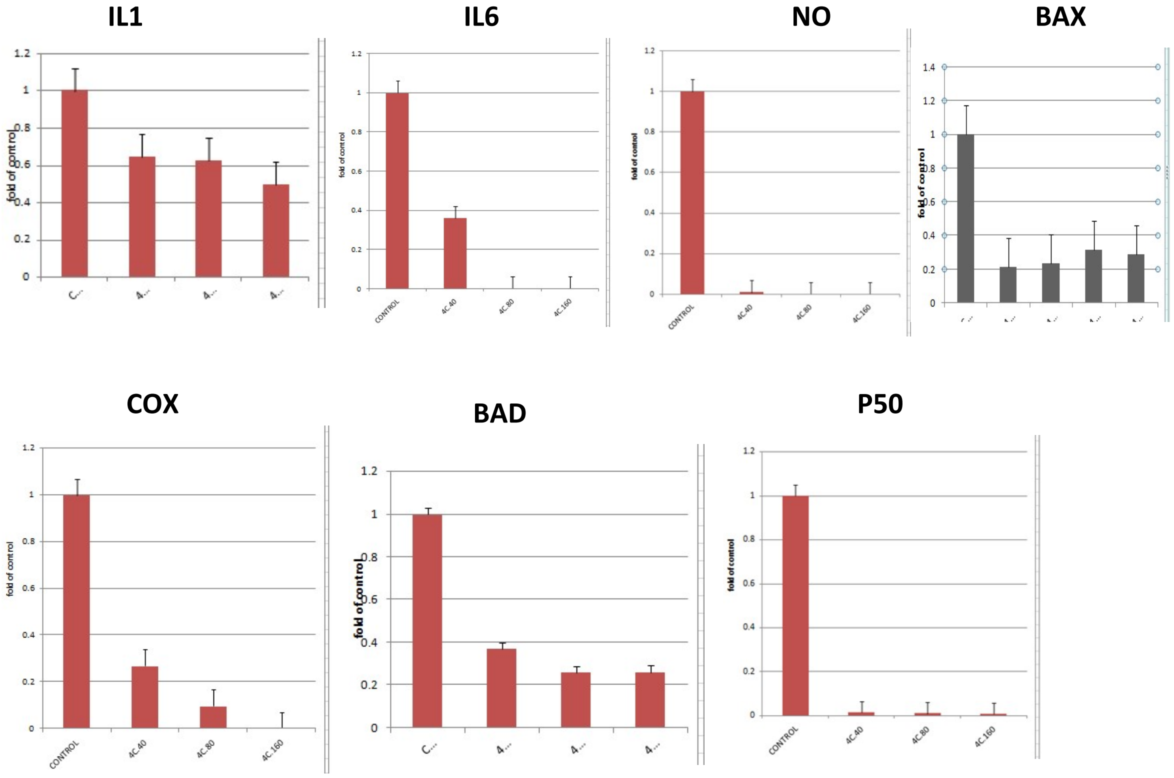

Fig. S5 RT-PCR study of complex **4c** against U87MG

Characterization of the RAPTA complexes

^{1}H NMR of 4a

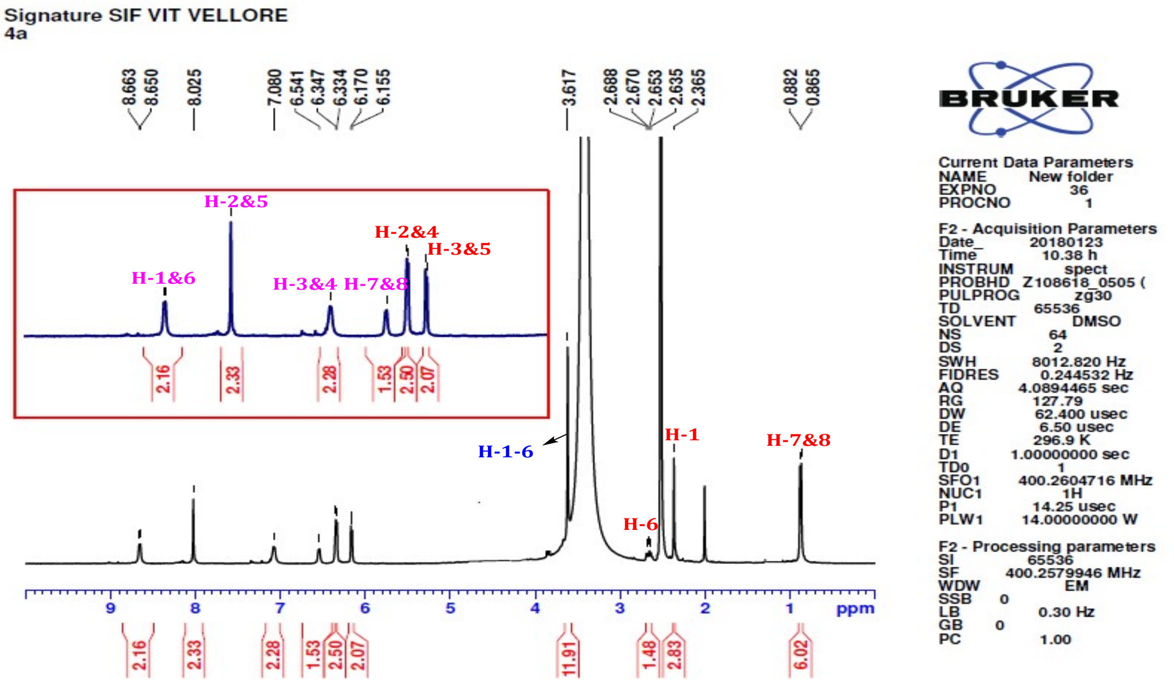

^{13}C NMR of 4a

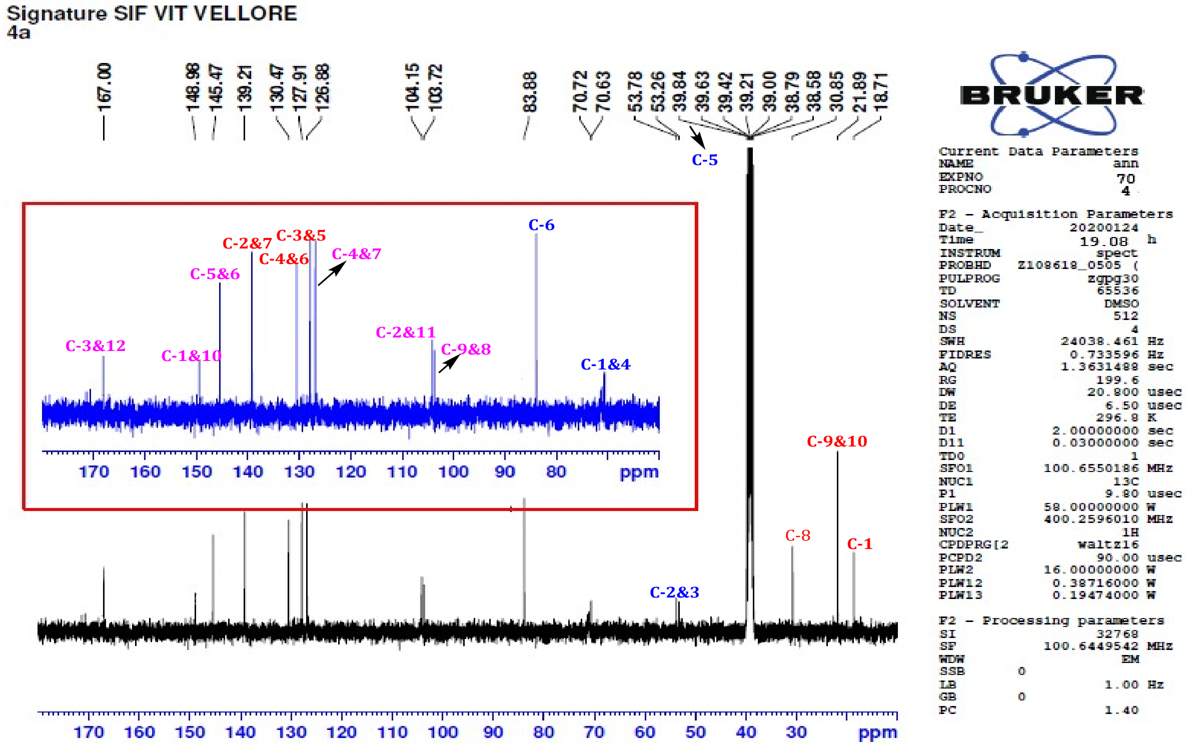

^{19}F NMR of 4a

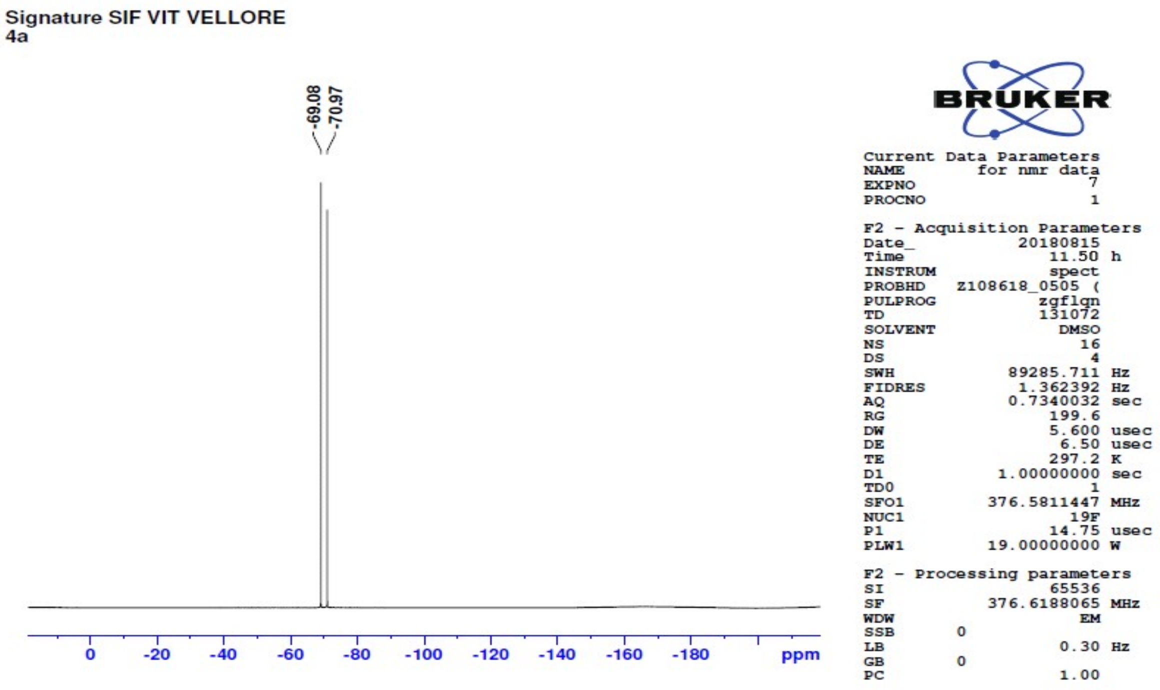

^{31}P NMR of 4a

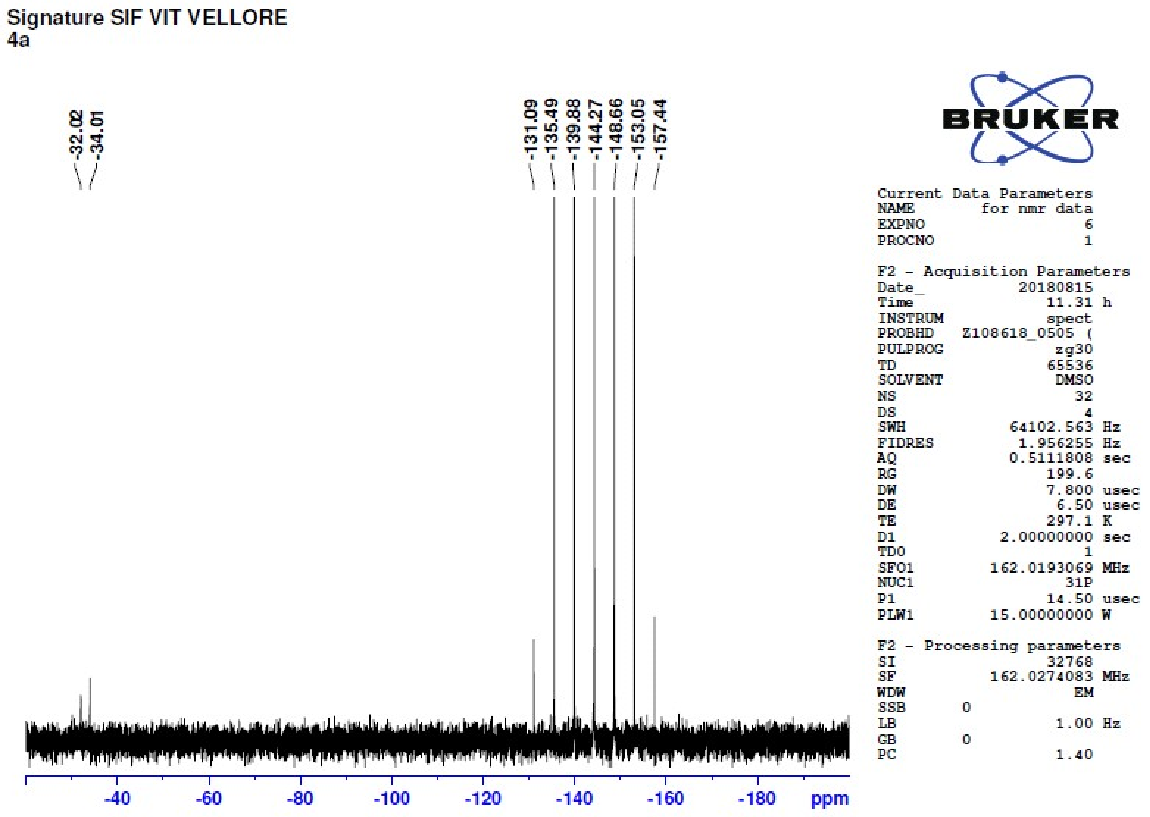

^{1}H NMR of 4b

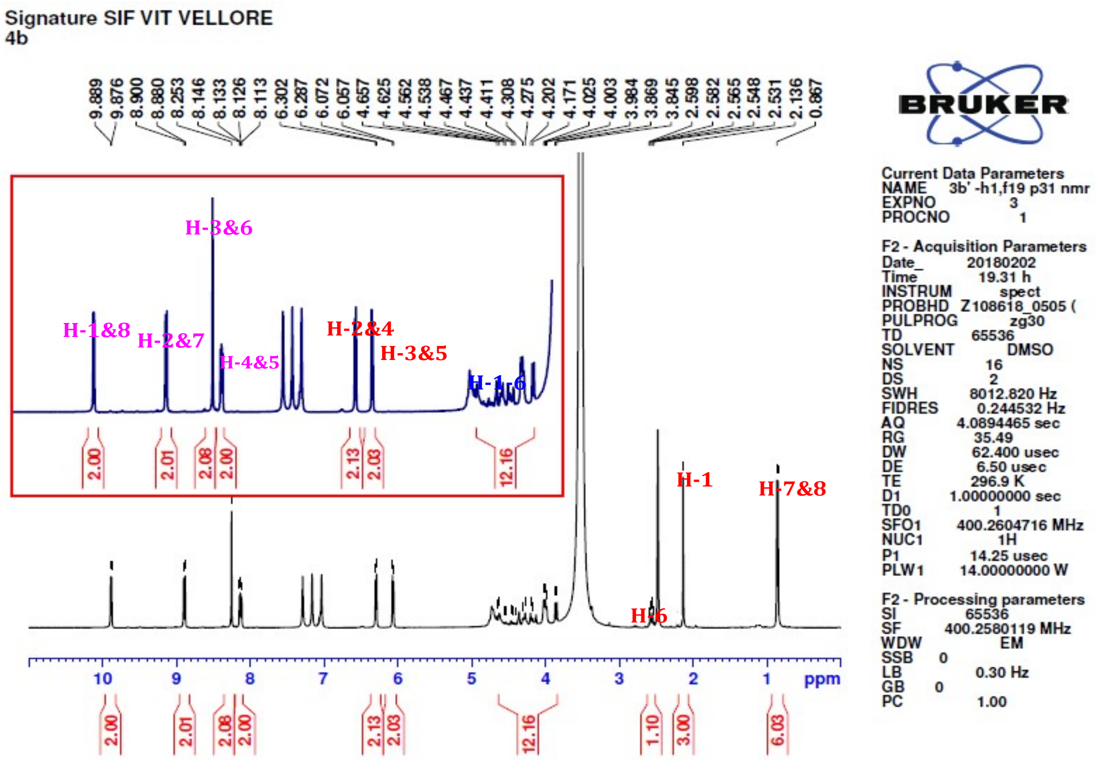

^{13}C NMR of 4b

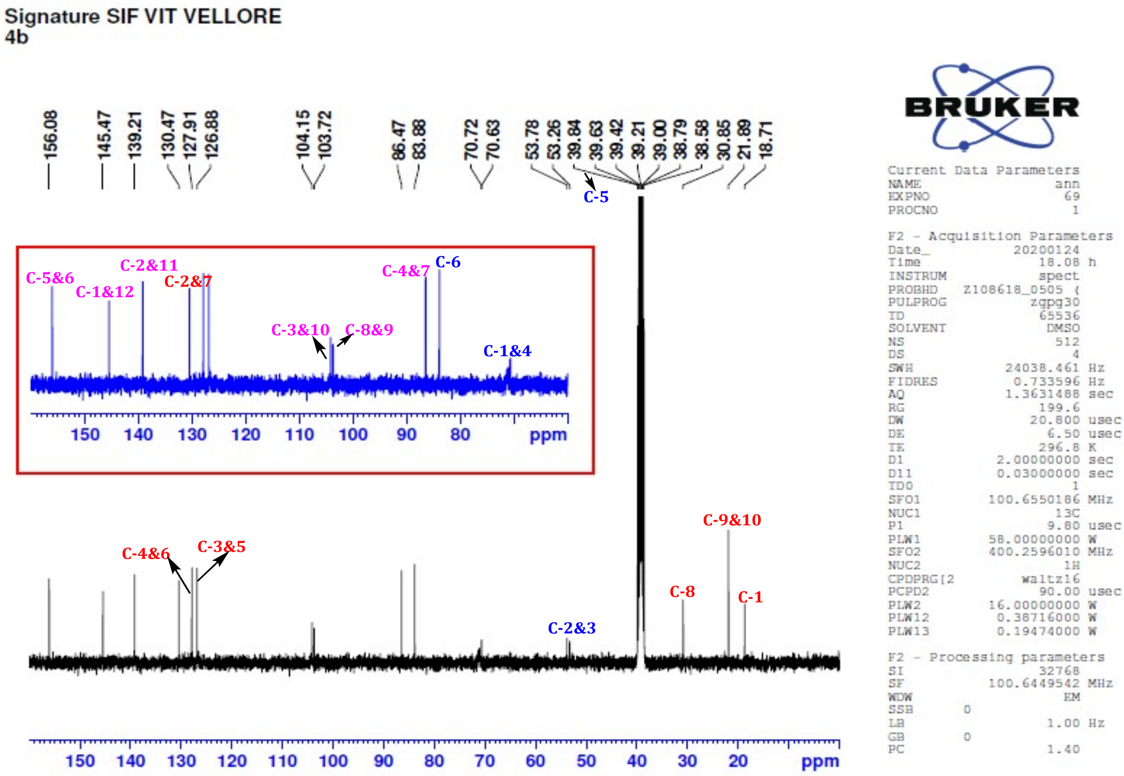

^{19}F NMR of 4b

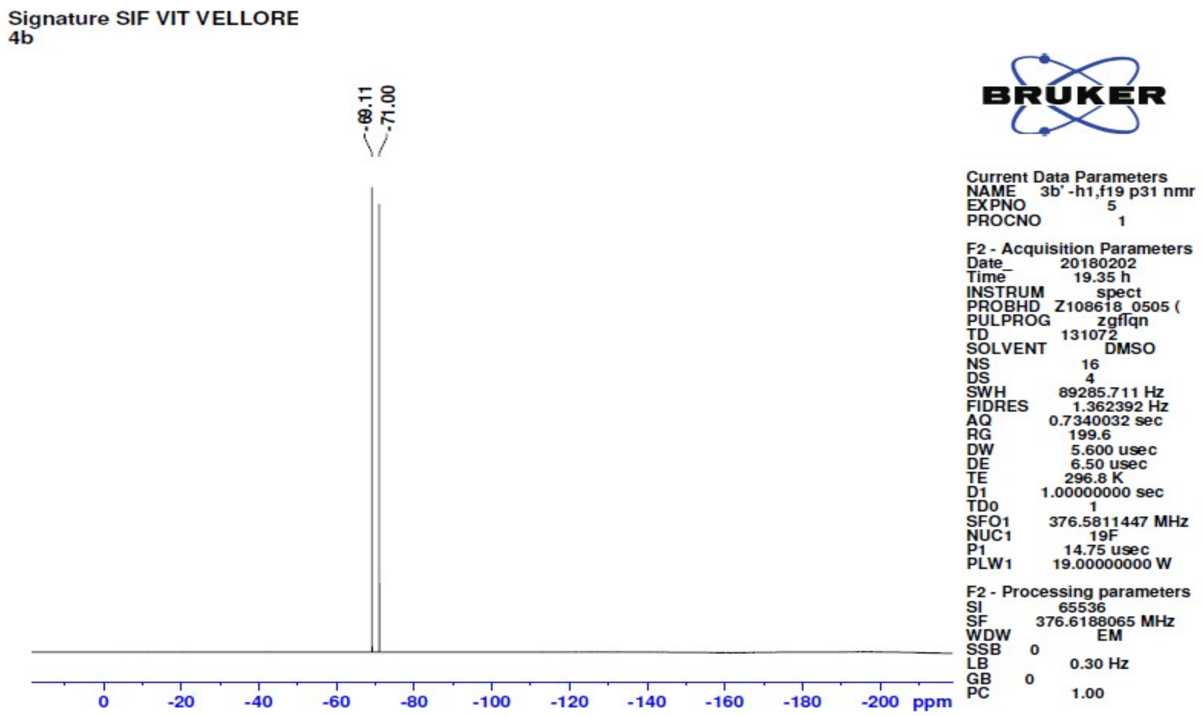

^{31}P NMR of 4b

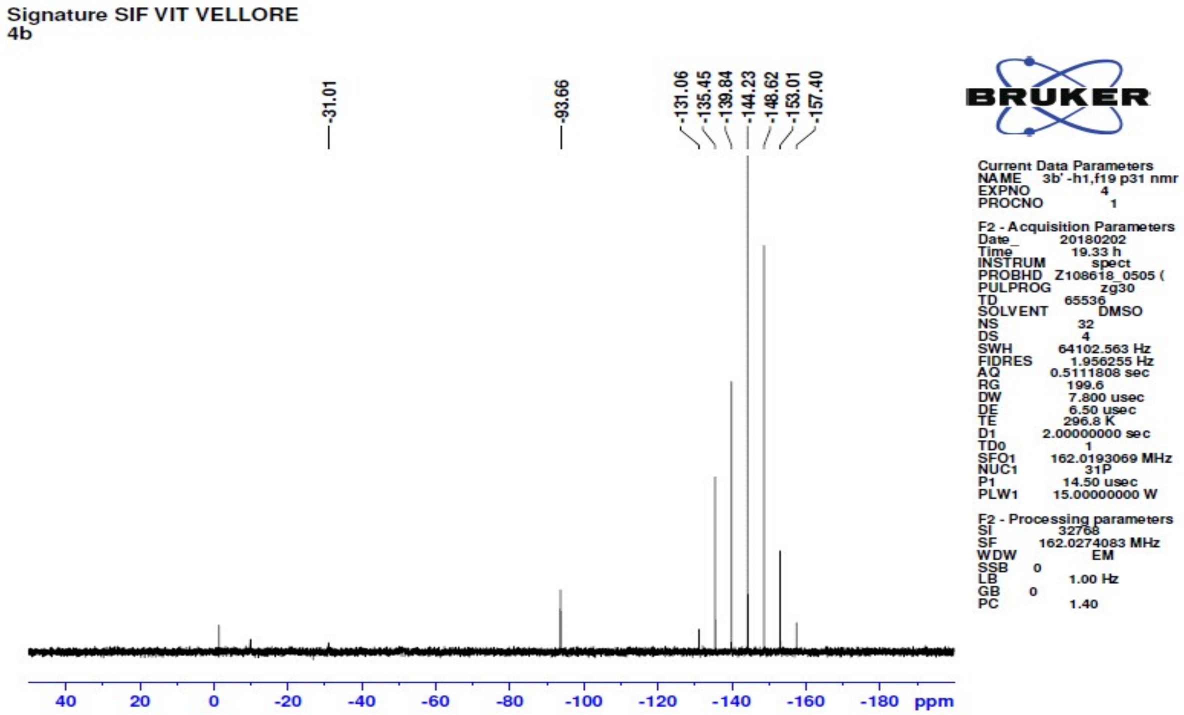

^{1}H NMR of 4c

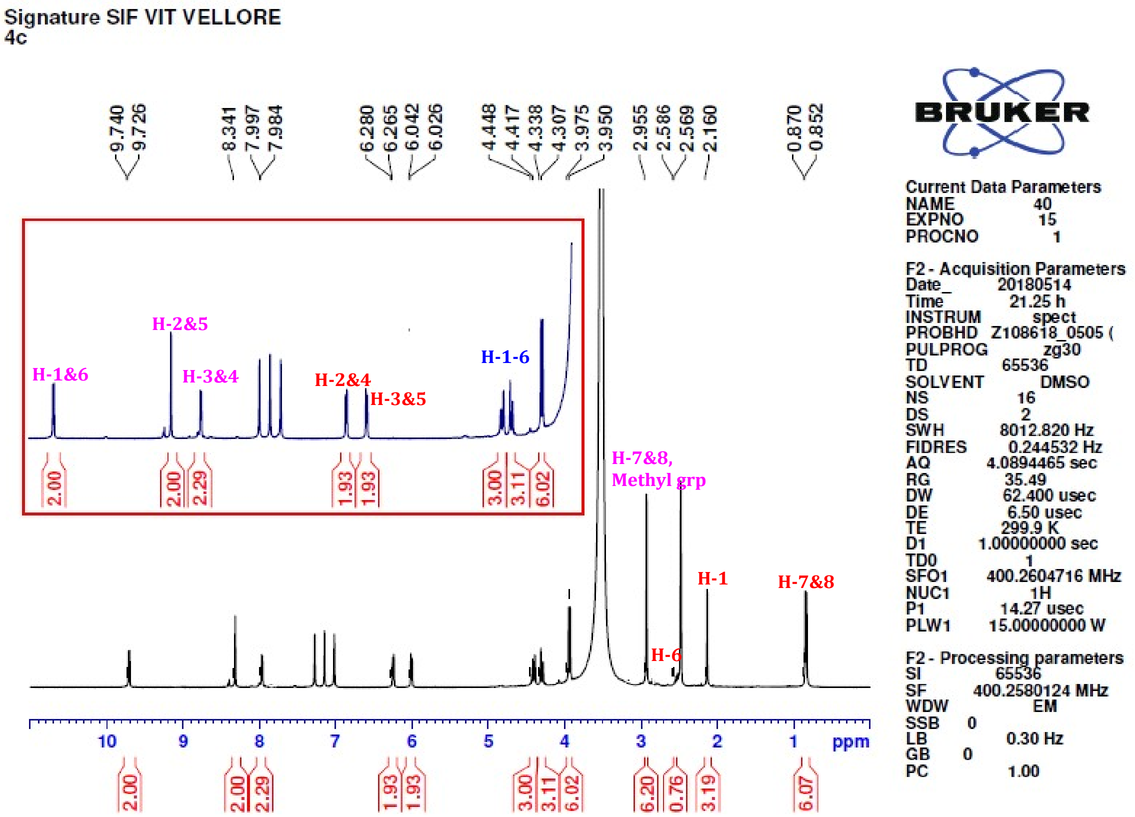

^{13}C NMR of 4c

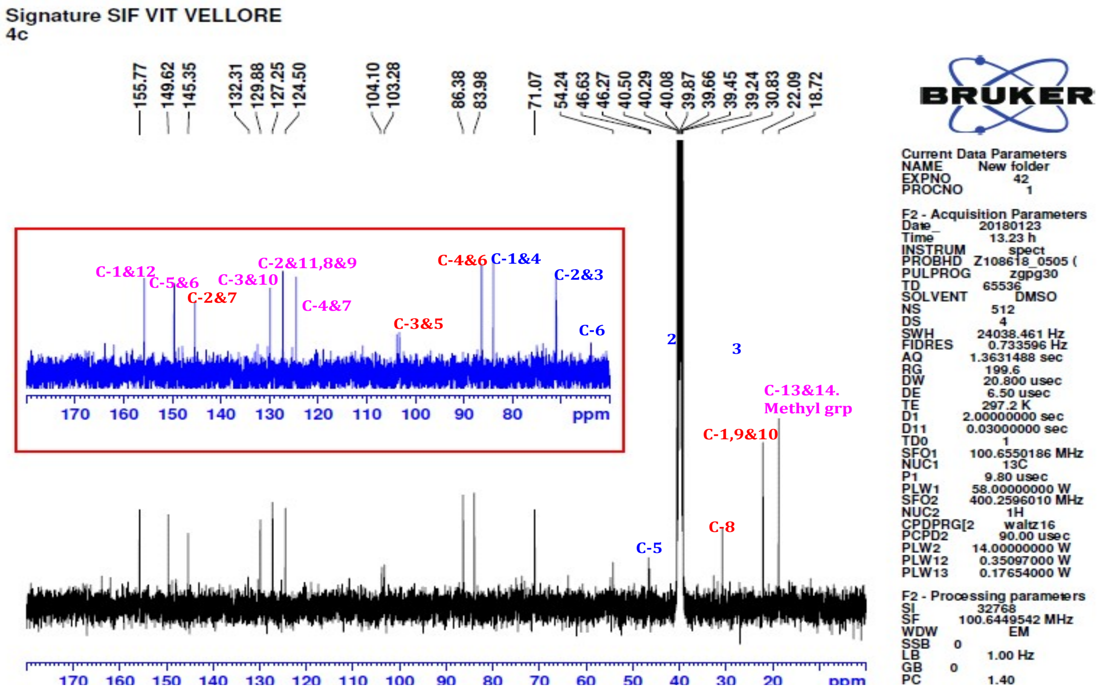

^{19}F NMR of 4c

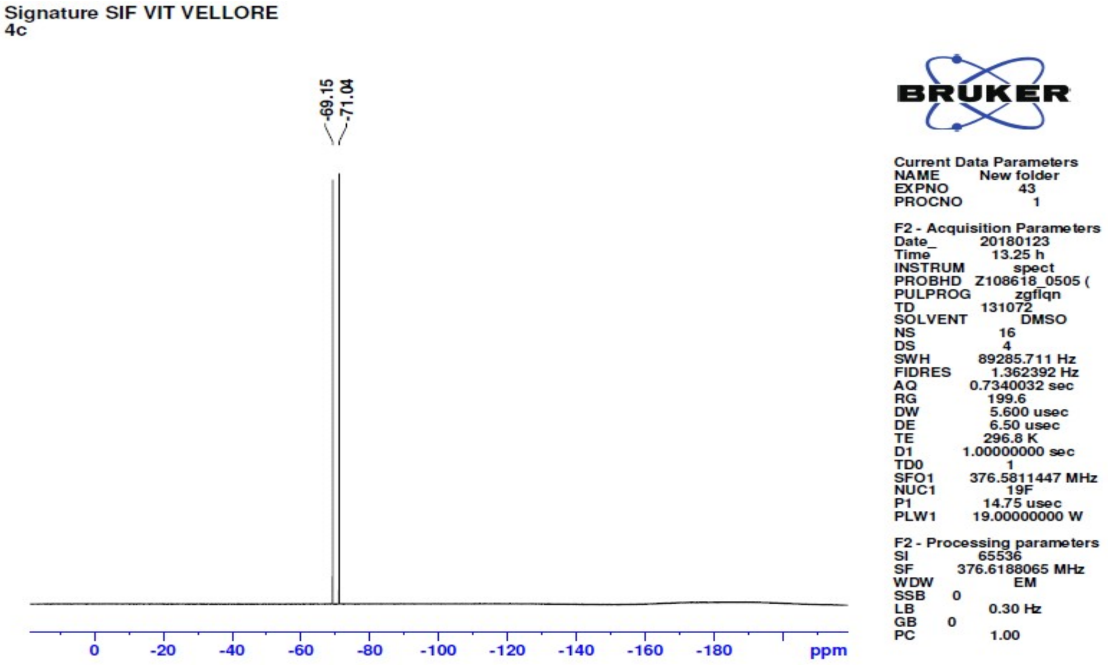

^{31}P NMR of 4c

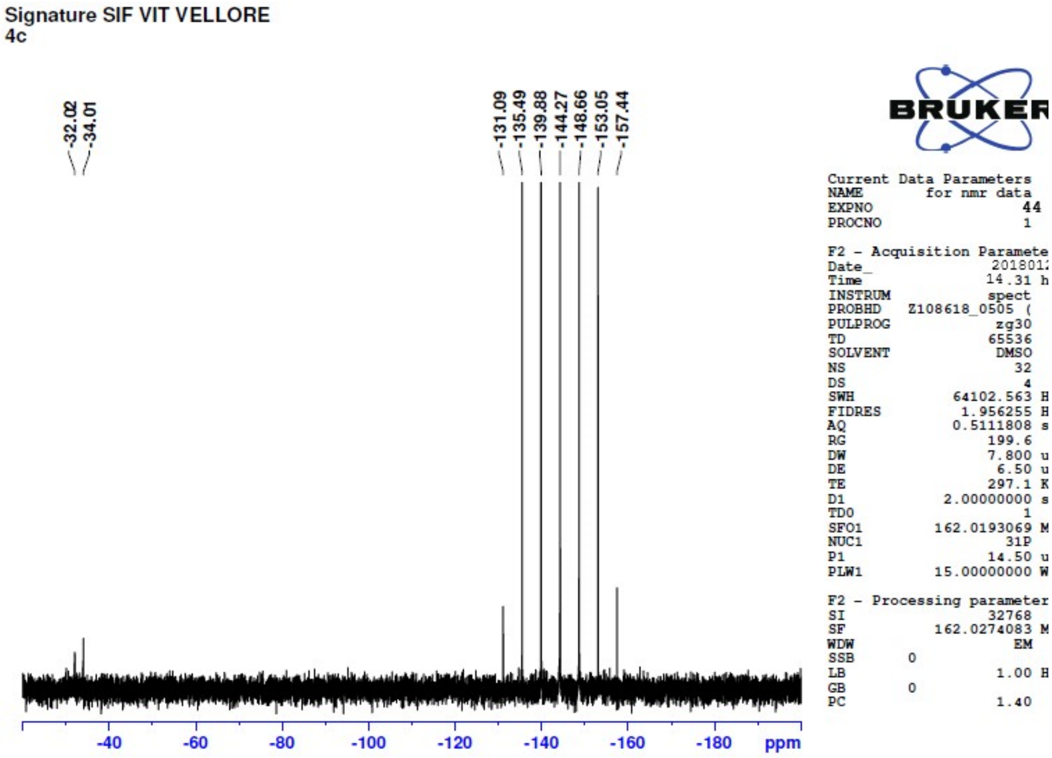

^{1}H NMR of 4d

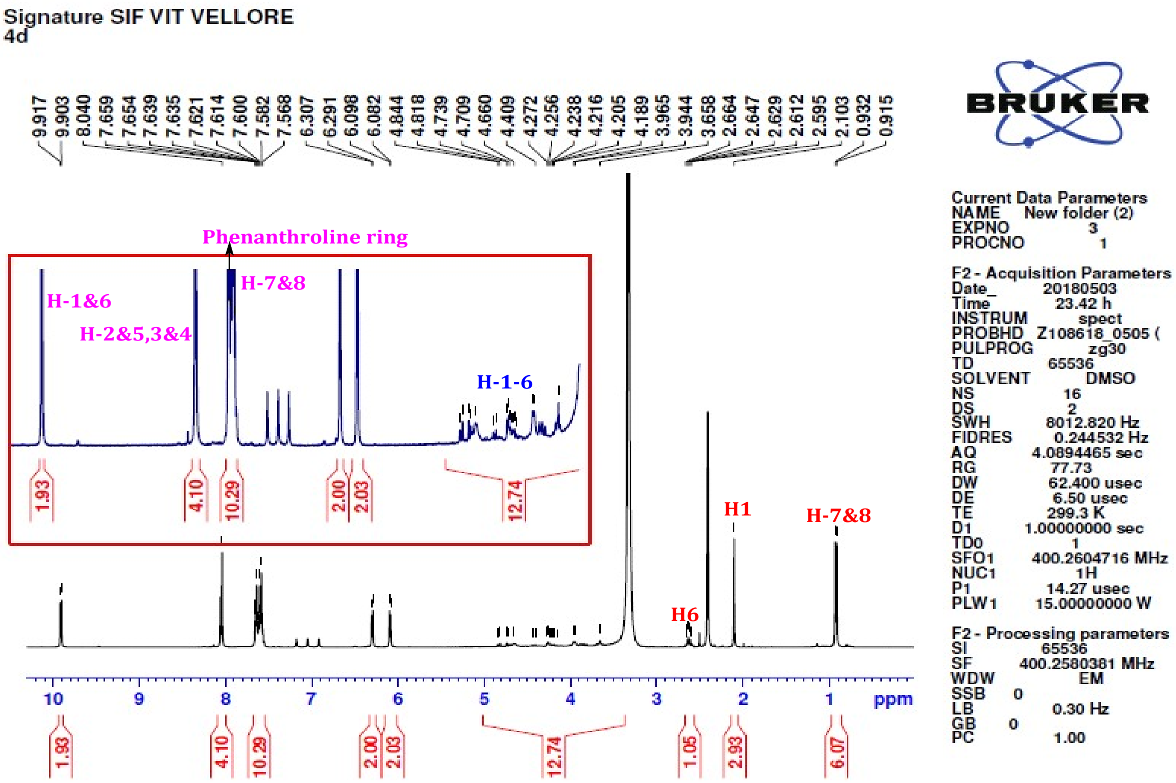

^{13}C NMR of 4d

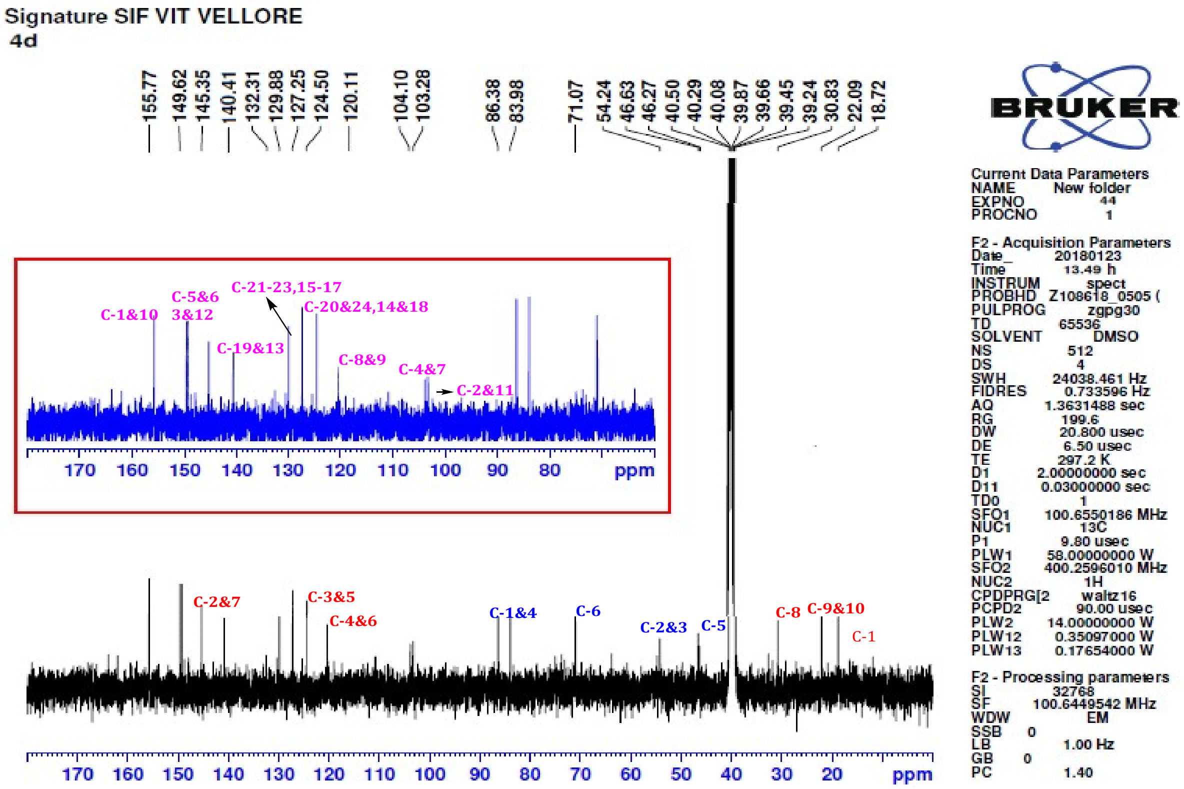

^{19}F NMR of 4d

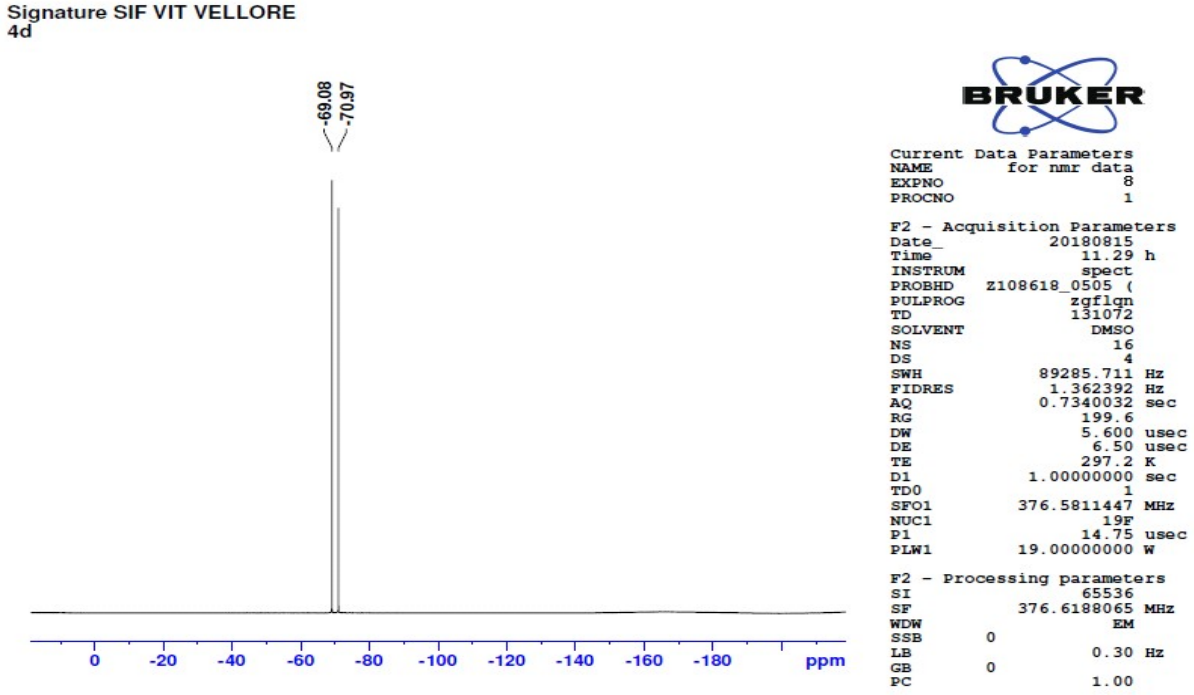

^{31}P NMR of 4d

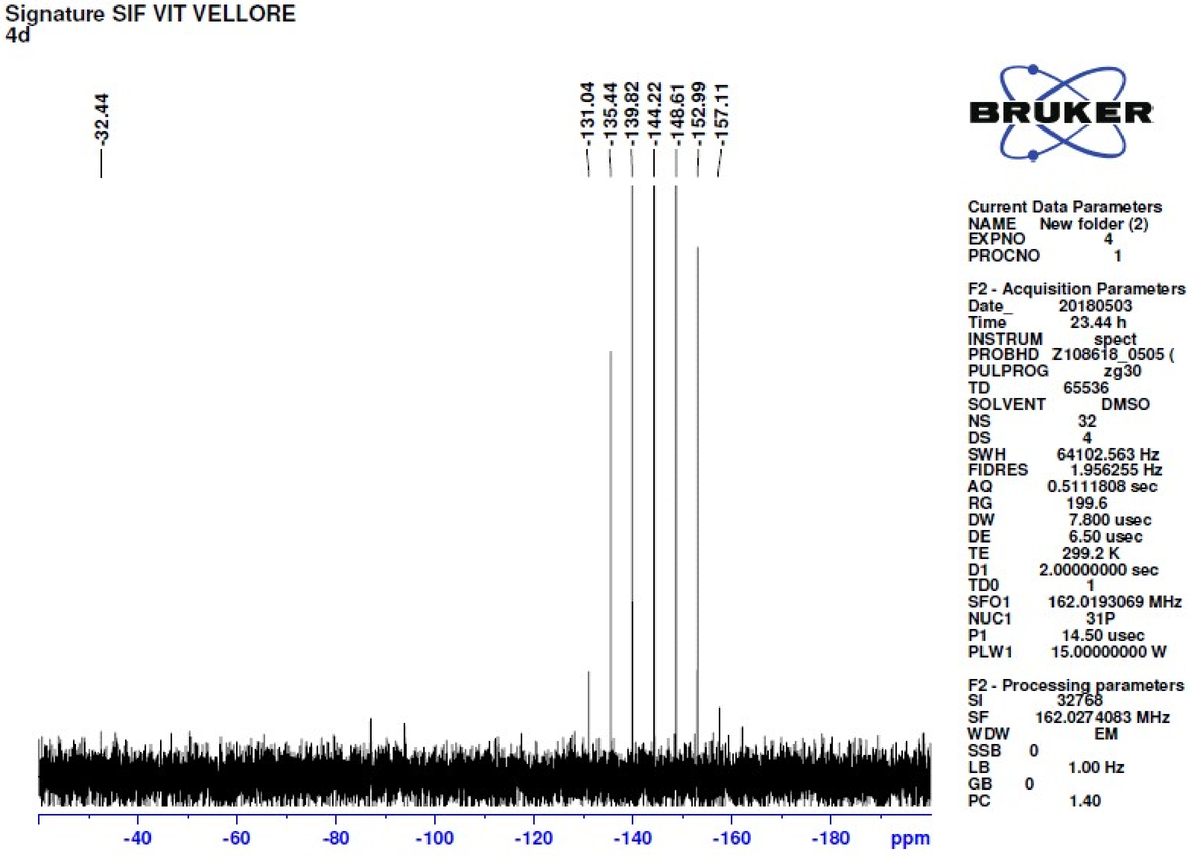

^{1}H NMR of 4e

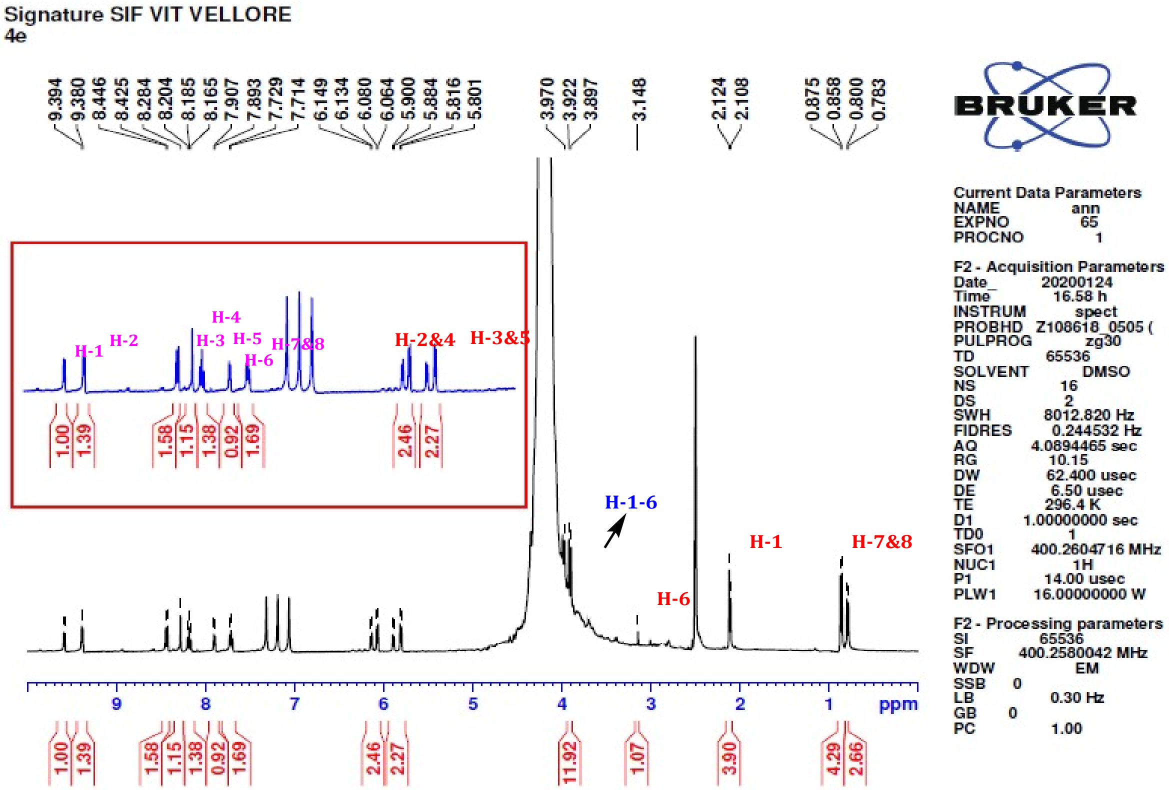

^{13}C NMR of 4e

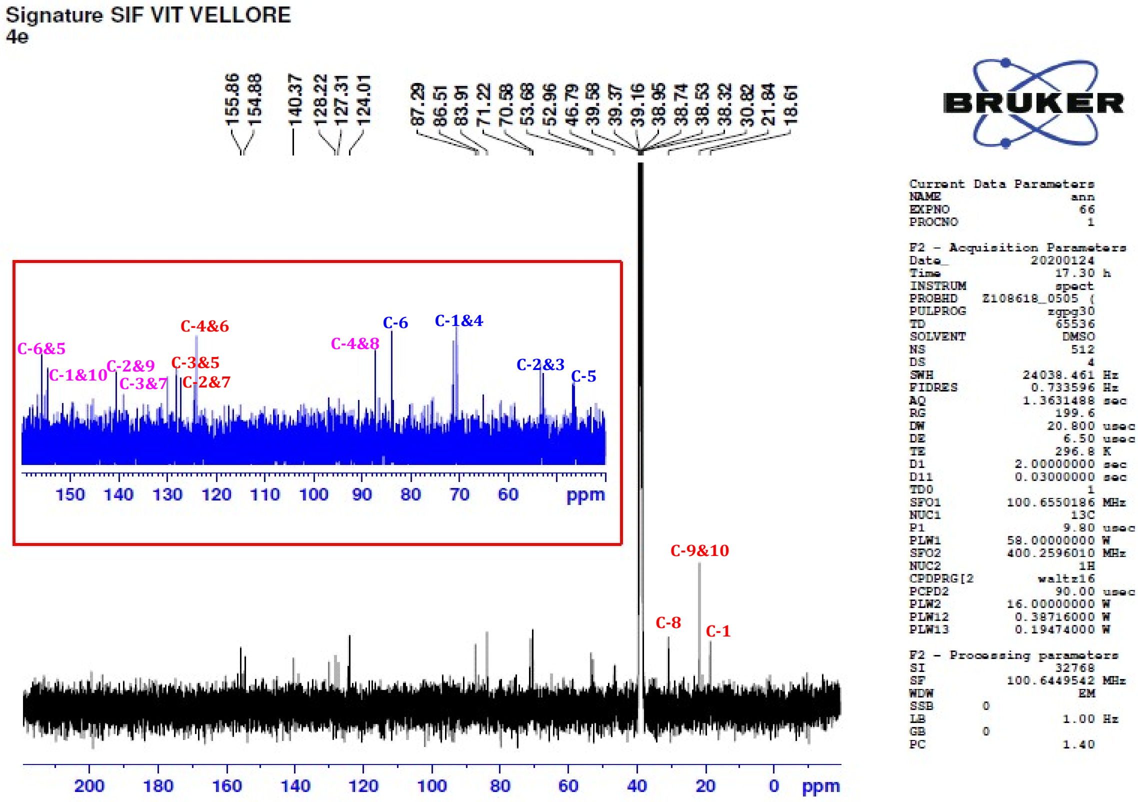

^{19}F NMR of 4e

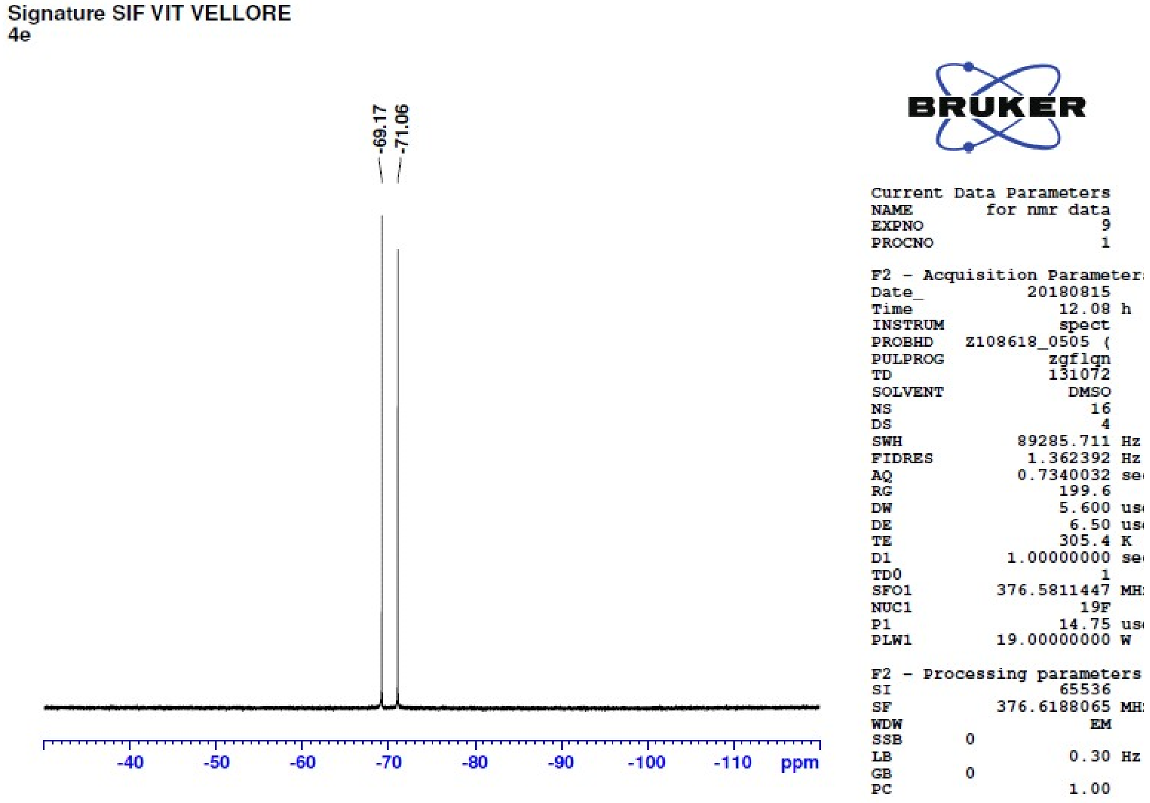

^{31}P NMR of 4e

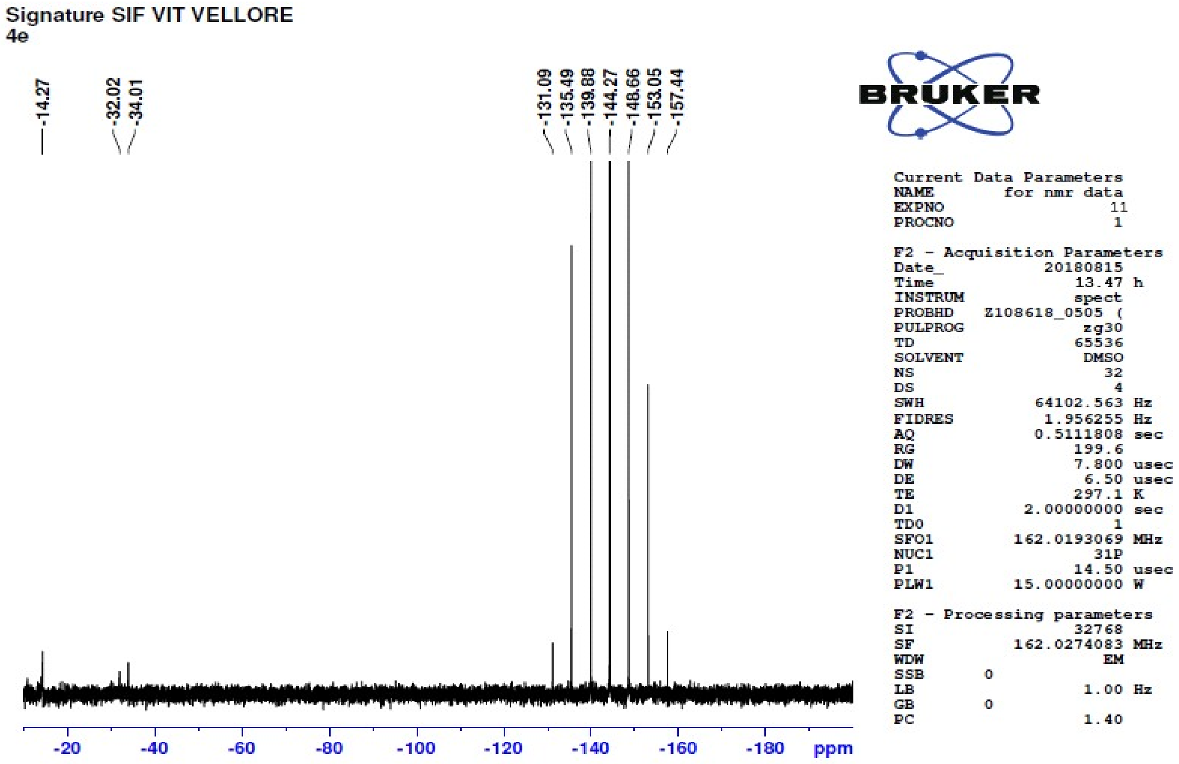

^{1}H NMR of 4f

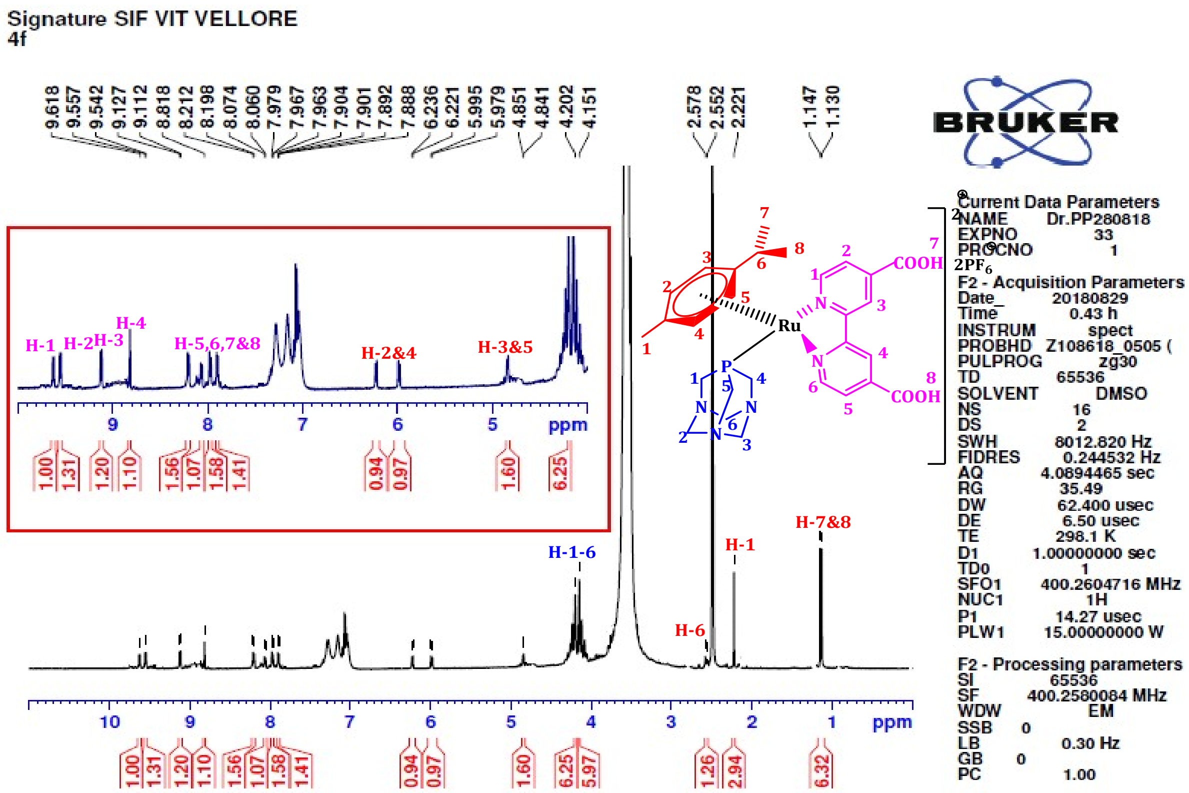

^{13}C NMR of 4f

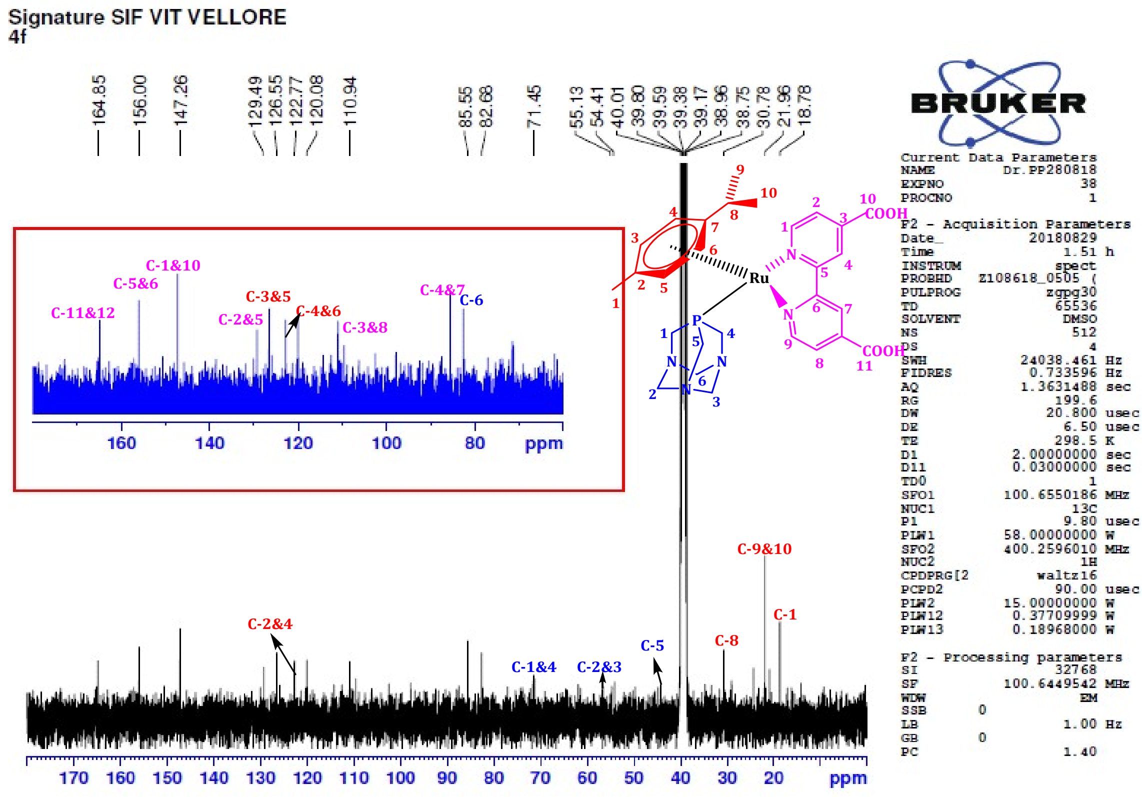

^{19}F NMR of 4f

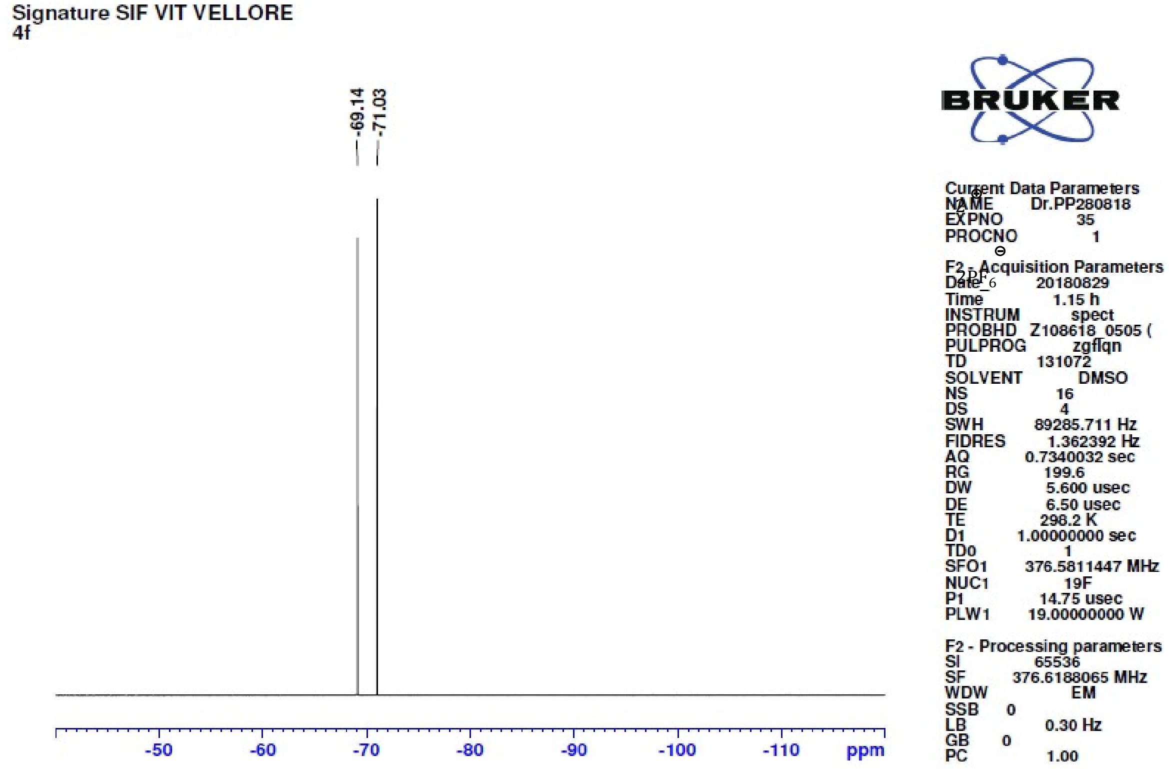

^{31}P NMR of 4f

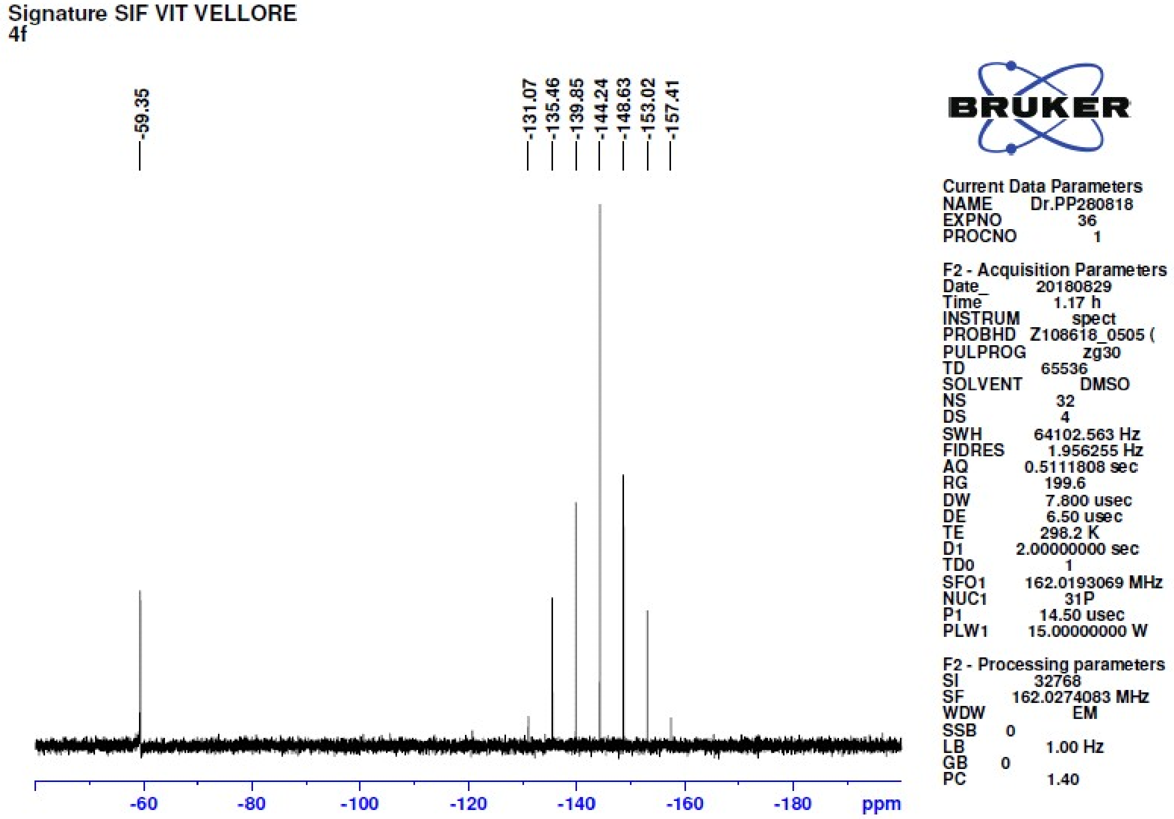

^{1}H NMR of 4g

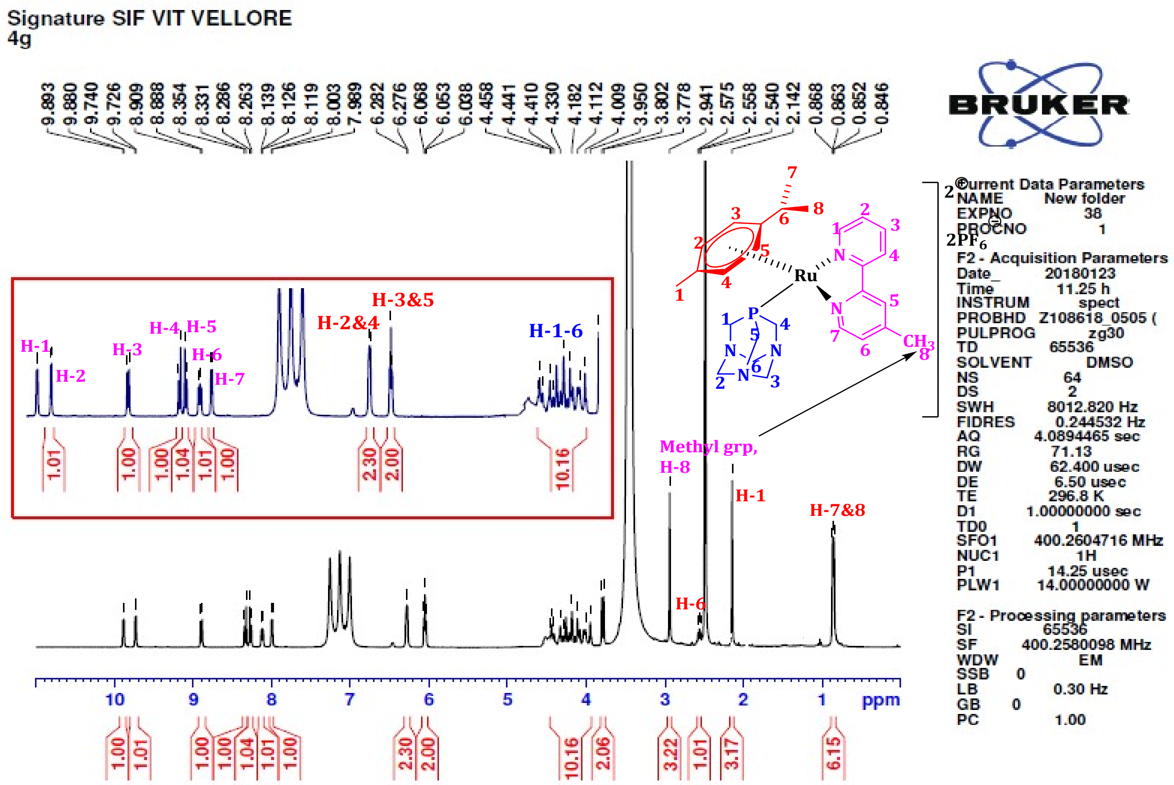

^{13}C NMR of 4g

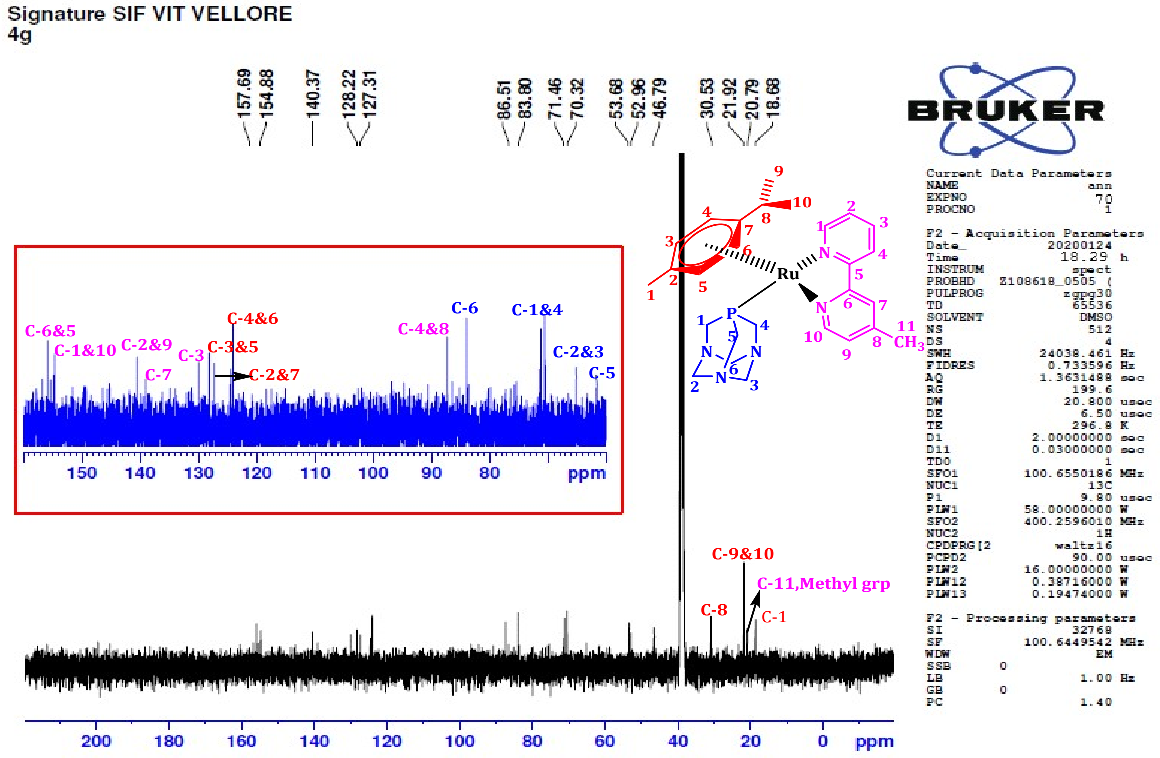

^{19}F NMR of 4g

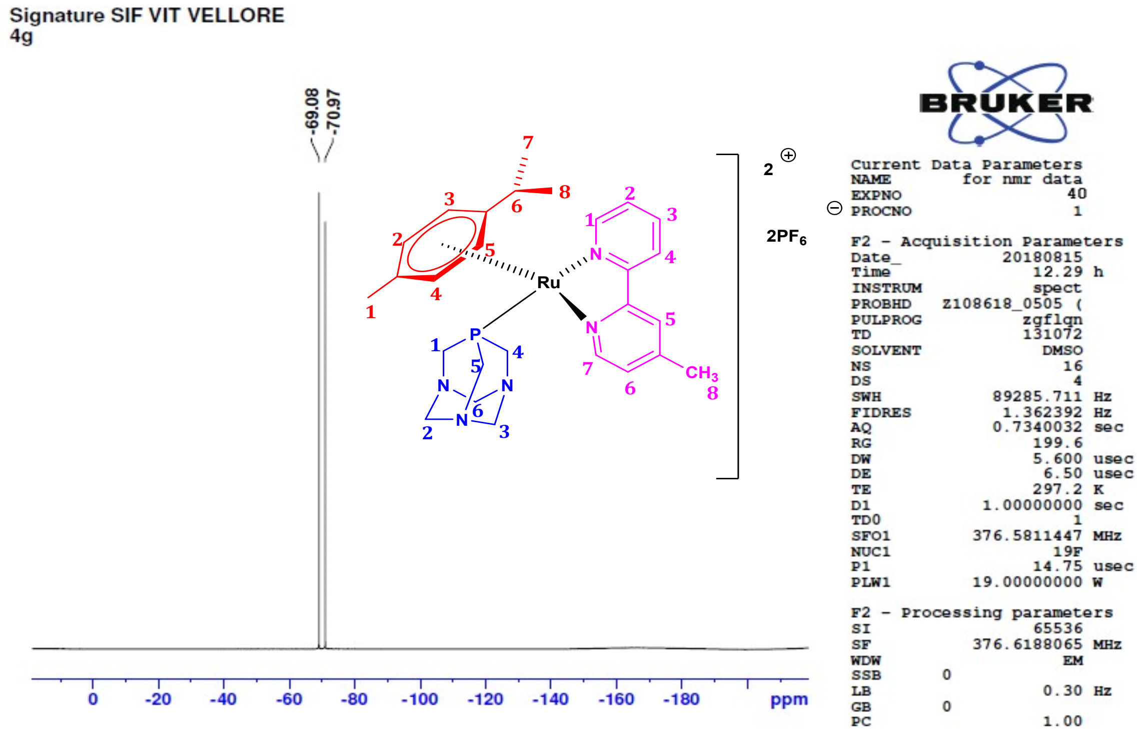

^{31}P NMR of 4g

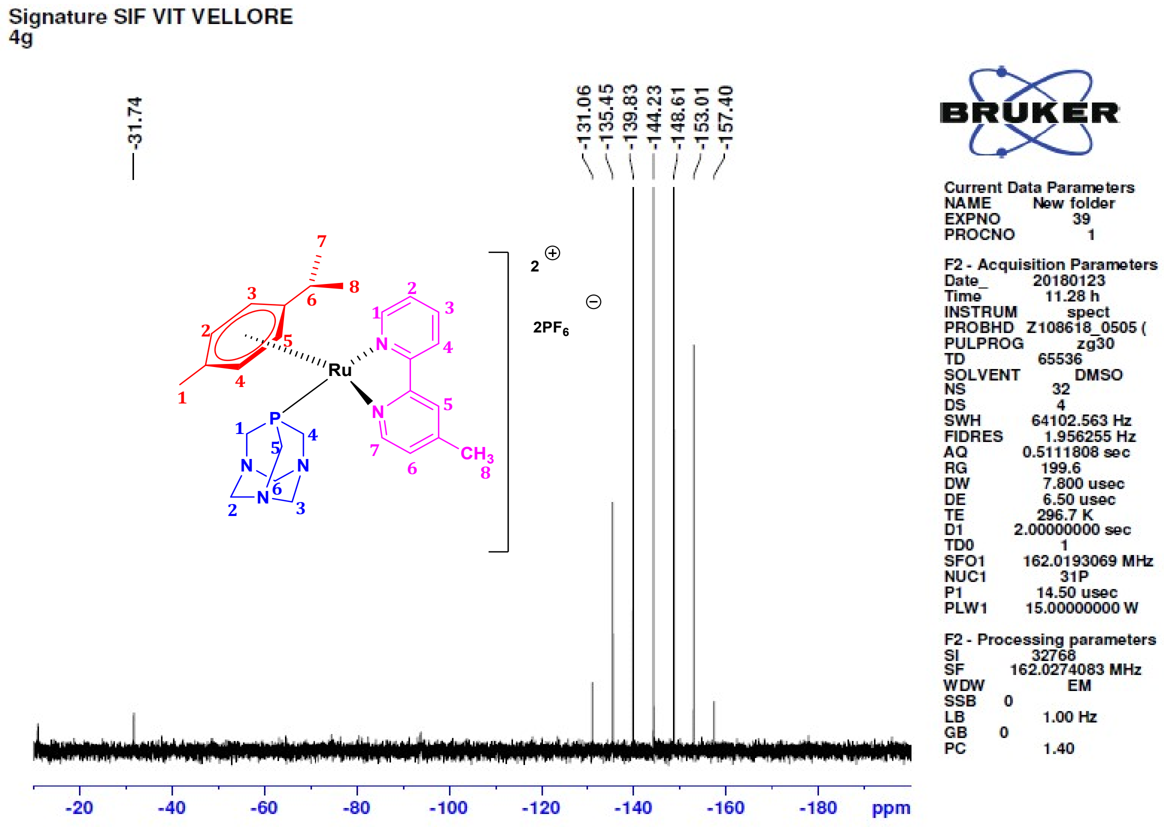

FT-IR Spectra

Complex 4a

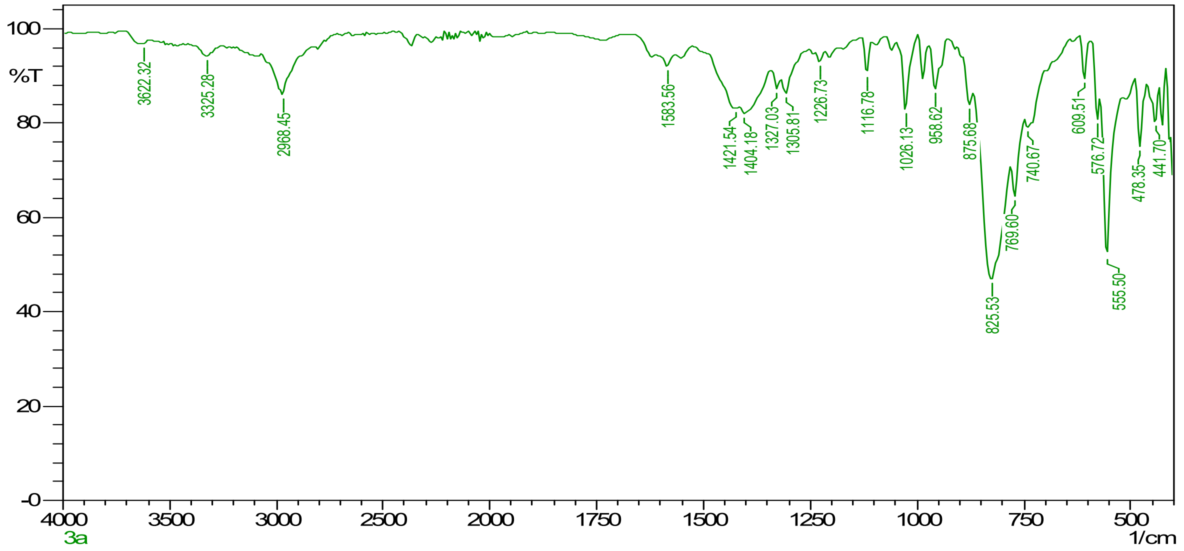

Complex 4b

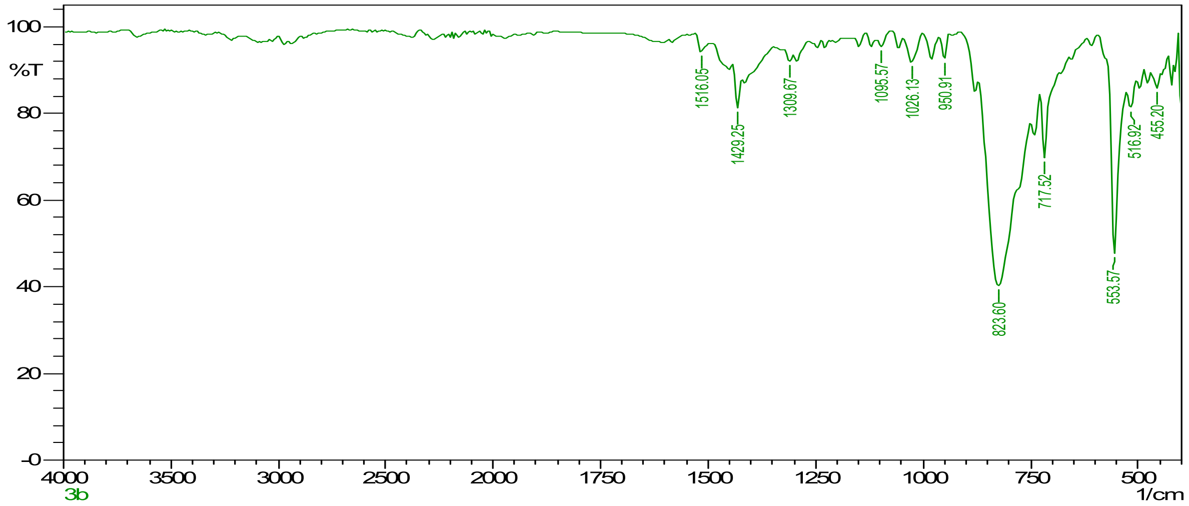

Complex 4c

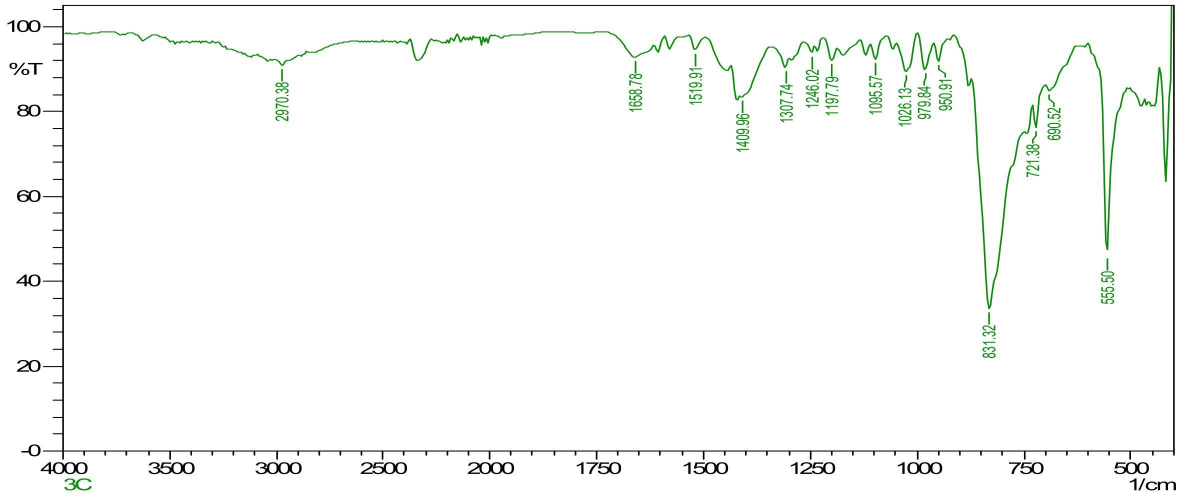

Complex 4d

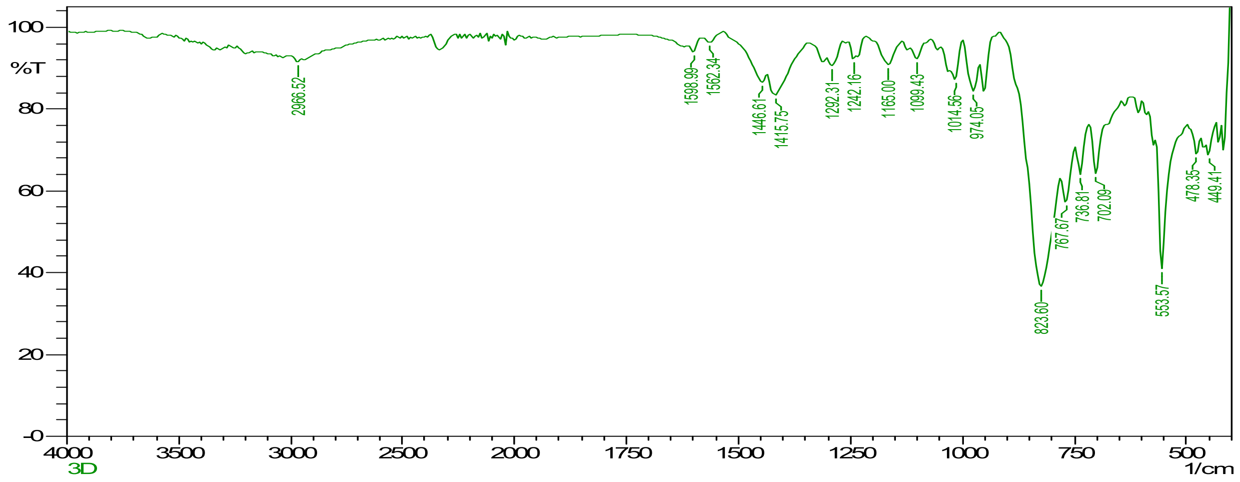

Complex 4e

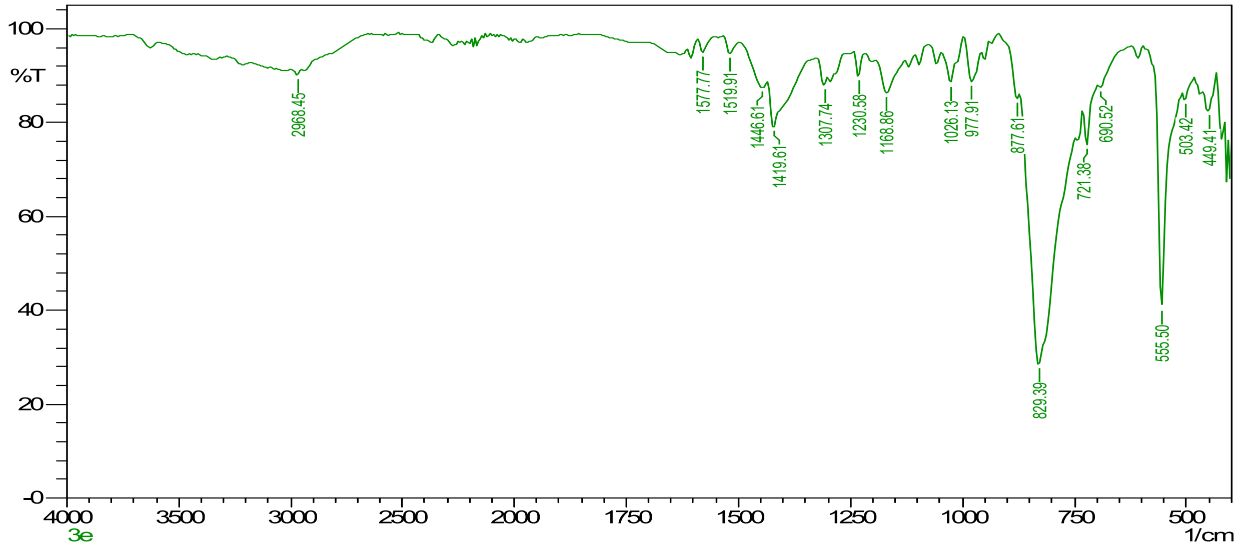

Complex 4f

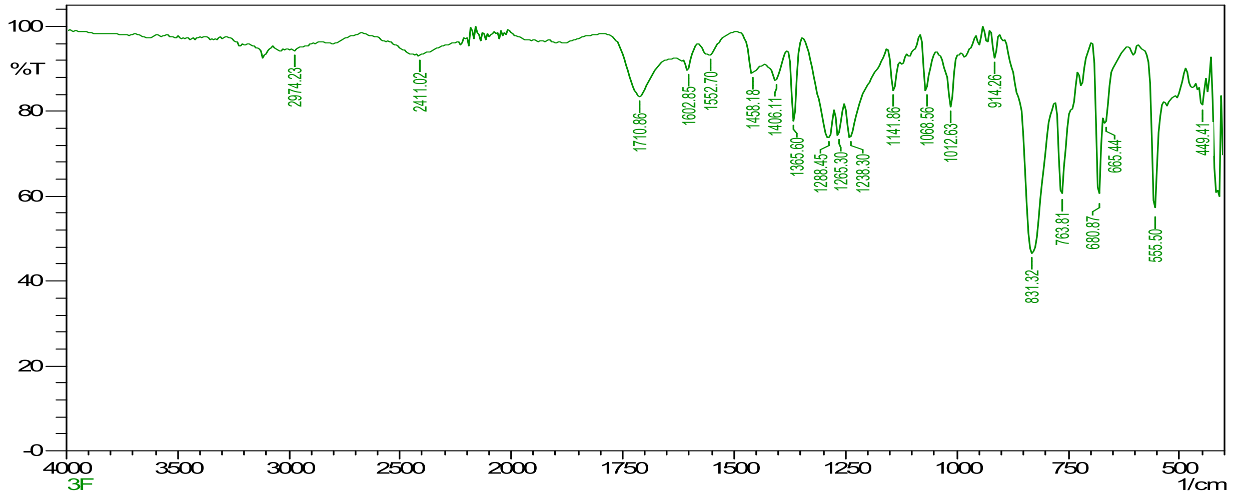

Complex 4g

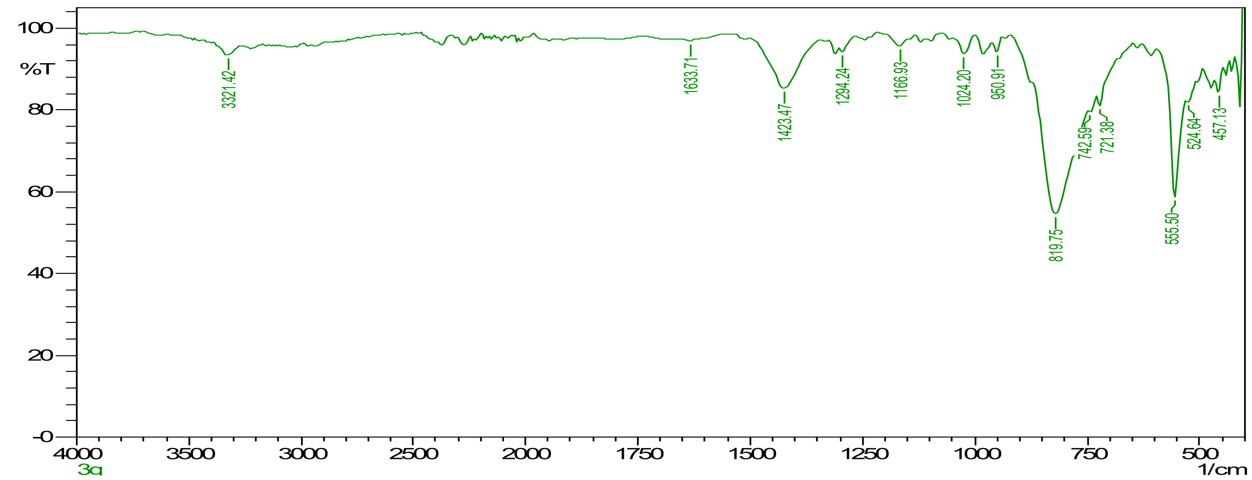
100
%T
80
60
40
20
-0
4000
3500
3000
2500
2000
1750
1500
1250
1000
750
500
3q
1/cm
3321.42
1633.71
1423.47
1294.24
1166.93
1024.20
950.91
819.75
742.59
721.38
555.50
524.64
457.13

Complex 4h

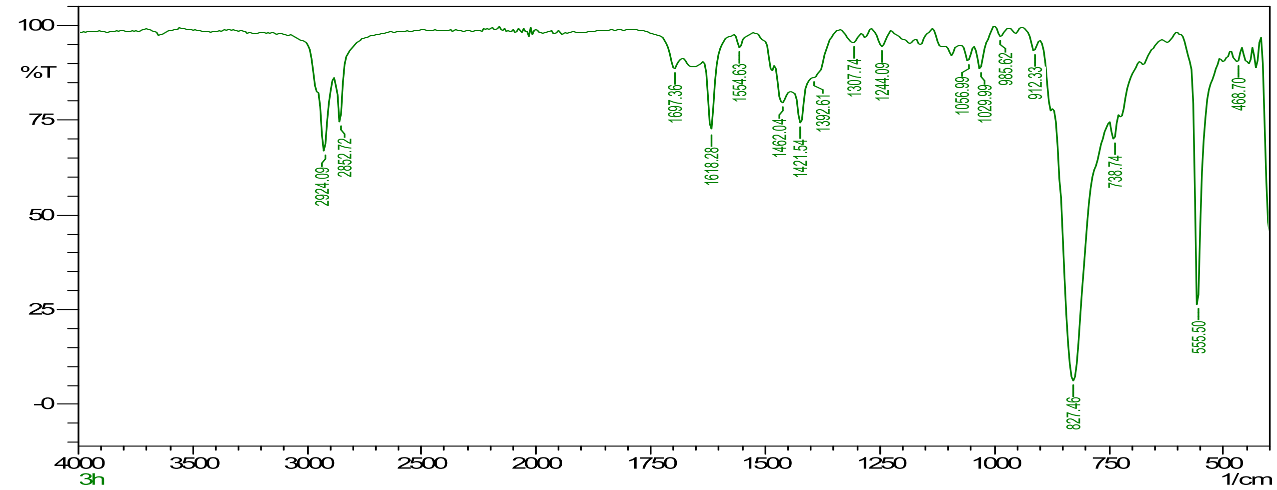
100
%T
75
50
25
-0
4000
3500
3000
2500
2000
1750
1500
1250
1000
750
500
3h
1/cm
2924.09
2852.72
1697.36
1618.28
1554.63
1462.04
1421.54
1392.61
1307.74
1244.09
1056.99
1029.99
985.62
912.33
827.46
738.74
555.50
468.70

Purity (UPLC)

Complex 4a

SAMPLE INFORMATION

Sample Name:	3a	Acquired By:	System
Sample Type:	Standard	Sample Set Name:	anuja
Vial:	1:F,1	Acq. Method Set:	VIT
Injection #:	1	Processing Method:	aishwarya
Injection Volume:	5.00 ul	Channel Name:	PDA Ch2 400nm@4.8nm
Run Time:	5.0 Minutes	Proc. Chnl. Descr.:	PDA Ch2 400nm@4.8nm
Date Acquired:	24-01-2019 11:08:48 IST		
Date Processed:	31-01-2019 11:53:36 IST		

Auto-Scaled Chromatogram

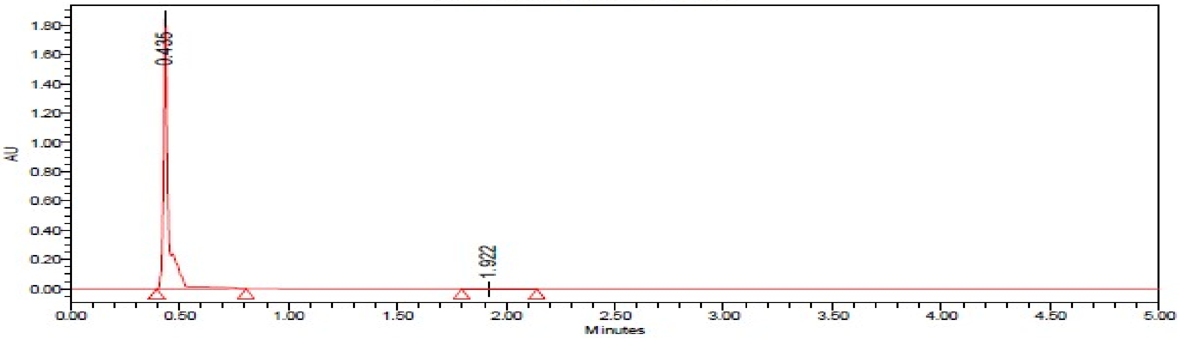

Peak Results

	Name	RT	Area	% Area
1		0.435	2883861	99.93
2		1.922	2069	0.07

Complex 4b

SAMPLE INFORMATION

Sample Name:	3b	Acquired By:	System
Sample Type:	Standard	Sample Set Name:	anuja
Vial:	1:F,2	Acq. Method Set:	VIT
Injection #:	1	Processing Method:	aishwarya
Injection Volume:	5.00 ul	Channel Name:	PDA Ch2 400nm@4.8nm
Run Time:	5.0 Minutes	Proc. Chnl. Descr.:	PDA Ch2 400nm@4.8nm
Date Acquired:	24-01-2019 11:15:27 IST		
Date Processed:	31-01-2019 11:54:06 IST		

Auto-Scaled Chromatogram

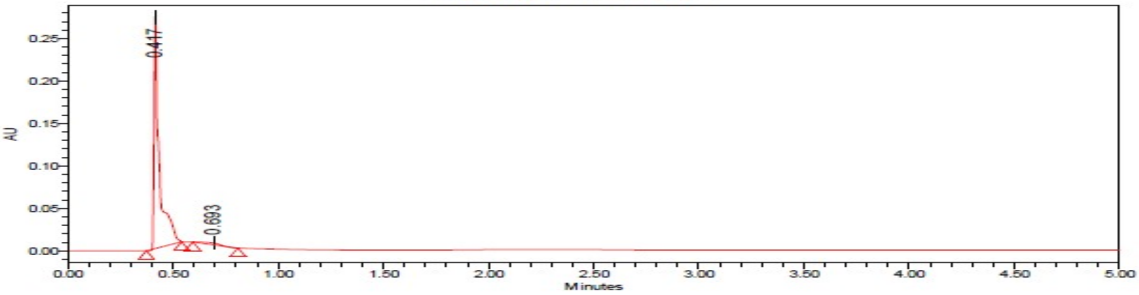

Peak Results

	Name	RT	Area	% Area
1		0.417	462395	97.22
2		0.693	13224	2.78

Complex 4c

SAMPLE INFORMATION

Sample Name:	3c	Acquired By:	System
Sample Type:	Standard	Sample Set Name:	anuja
Vial:	1:F,3	Acq. Method Set:	VIT
Injection #:	1	Processing Method:	aishwarya
Injection Volume:	5.00 ul	Channel Name:	PDA Ch2 400nm@4.8nm
Run Time:	5.0 Minutes	Proc. Chnl. Descr.:	PDA Ch2 400nm@4.8nm
Date Acquired:	24-01-2019 11:22:04 IST		
Date Processed:	31-01-2019 11:54:44 IST		

Auto-Scaled Chromatogram

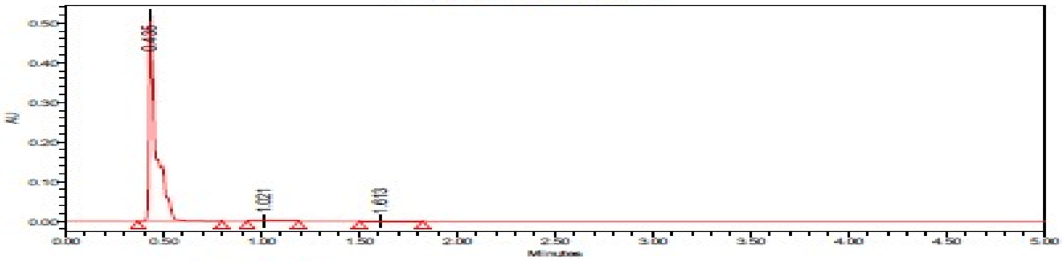

Peak Results

	Name	RT	Area	% Area
1		0.435	1318484	99.75
2		1.021	1239	0.09
3		1.613	2058	0.16

Reported by User: System
Report Method: siva
Report Method ID [illegible]
Page: 1 of 1

Project Name: VIT TBI
Date Printed:
15-03-2019
13:54:39 Asia/Kolkata

Complex 4d

SAMPLE INFORMATION

Sample Name:	3d	Acquired By:	System
Sample Type:	Standard	Sample Set Name:	anuja
Vial:	1:F,4	Acq. Method Set:	VIT
Injection #:	1	Processing Method:	aishwarya
Injection Volume:	5.00 ul	Channel Name:	PDA Ch2 400nm@4.8nm
Run Time:	5.0 Minutes	Proc. Chnl. Descr.:	PDA Ch2 400nm@4.8nm
Date Acquired:	24-01-2019 11:28:41 IST		
Date Processed:	31-01-2019 11:55:28 IST		

Auto-Scaled Chromatogram

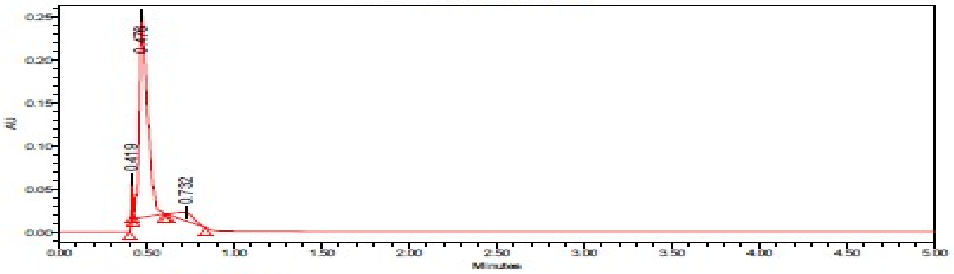

Peak Results

	Name	RT	Area	% Area
1		0.419	17060	2.05
2		0.476	749648	89.96
3		0.732	66603	7.99

Reported by User: System
Report Method: siva
Report Method ID [illegible]
Page: 1 of 1

Project Name: VIT TBI
Date Printed:
15-03-2019
13:54:25 Asia/Kolkata

Complex 4e

SAMPLE INFORMATION

Sample Name:	3e	Acquired By:	System
Sample Type:	Standard	Sample Set Name:	anuja
Vial:	1:F,5	Acq. Method Set:	VIT
Injection #:	1	Processing Method:	aishwarya
Injection Volume:	5.00 ul	Channel Name:	PDA Ch2 400nm@4.8nm
Run Time:	5.0 Minutes	Proc. Chnl. Descr.:	PDA Ch2 400nm@4.8nm
Date Acquired:	24-01-2019 11:35:15 IST		
Date Processed:	31-01-2019 11:56:18 IST		

Auto-Scaled Chromatogram

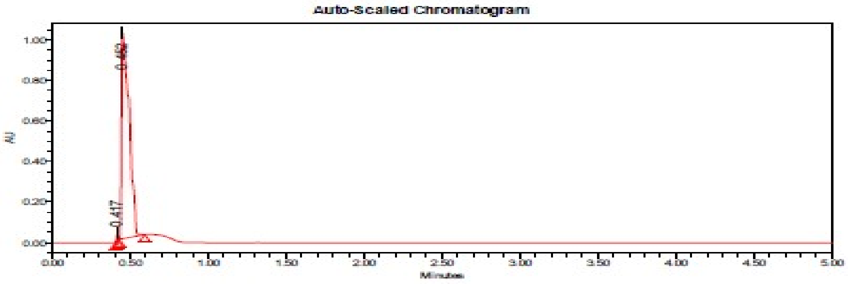

Peak Results

	Name	RT	Area	% Area
1		0.417	15803	0.46
2		0.452	3420948	99.54

Reported by User: System
Report Method: siva
Report Method ID 5650
Page: 1 of 1

Project Name: VIT TBI
Date Printed:
15-03-2019
13:54:12 Asia/Kolkata

Complex 4f

SAMPLE INFORMATION

Sample Name:	3f	Acquired By:	System
Sample Type:	Standard	Sample Set Name:	anuja
Vial:	1:F,6	Acq. Method Set:	VIT
Injection #:	1	Processing Method:	aishwarya
Injection Volume:	5.00 ul	Channel Name:	PDA Ch2 400nm@4.8nm
Run Time:	5.0 Minutes	Proc. Chnl. Descr.:	PDA Ch2 400nm@4.8nm
Date Acquired:	24-01-2019 11:41:51 IST		
Date Processed:	31-01-2019 11:57:02 IST		

Auto-Scaled Chromatogram

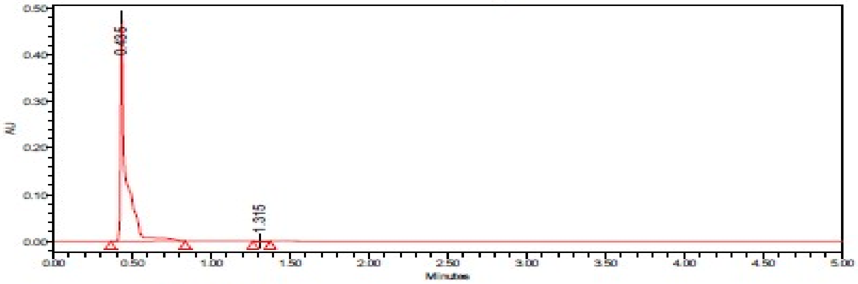

Peak Results

	Name	RT	Area	% Area
1		0.435	1142377	99.99
2		1.315	78	0.01

Reported by User: System
Report Method: siva
Report Method ID 5650
Page: 1 of 1

Project Name: VIT TBI
Date Printed:
15-03-2019
13:53:53 Asia/Kolkata

Complex 4g

SAMPLE INFORMATION

Sample Name:	3g	Acquired By:	System
Sample Type:	Standard	Sample Set Name:	anuja
Vial:	1:F,7	Acq. Method Set:	VIT
Injection #:	1	Processing Method:	aishwarya
Injection Volume:	5.00 ul	Channel Name:	PDA Ch2 400nm@4.8nm
Run Time:	5.0 Minutes	Proc. Chnl. Descr.:	PDA Ch2 400nm@4.8nm
Date Acquired:	24-01-2019 11:48:26 IST		
Date Processed:	31-01-2019 11:58:31 IST		

Auto-Scaled Chromatogram

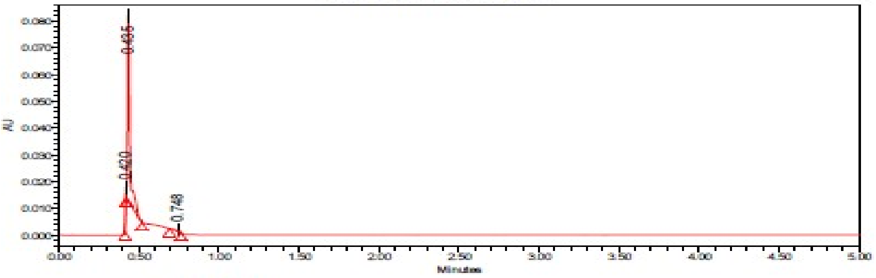

Peak Results

	Name	RT	Area	% Area
1		0.420	2589	3.25
2		0.435	74683	94.50
3		0.748	1772	2.24

Reported by User: System
Report Method: siva
Report Method ID: 5560
Page: 1 of 1

Project Name: VIT TBI
Date Printed:
15-03-2019
13:53:35 Asia/Kolkata

Complex 4h

SAMPLE INFORMATION

Sample Name:	3h	Acquired By:	System
Sample Type:	Standard	Sample Set Name:	anuja
Vial:	1:F,8	Acq. Method Set:	VIT
Injection #:	1	Processing Method:	aishwarya
Injection Volume:	5.00 ul	Channel Name:	PDA Ch2 400nm@4.8nm
Run Time:	5.0 Minutes	Proc. Chnl. Descr.:	PDA Ch2 400nm@4.8nm
Date Acquired:	24-01-2019 11:55:03 IST		
Date Processed:	31-01-2019 11:59:07 IST		

Auto-Scaled Chromatogram

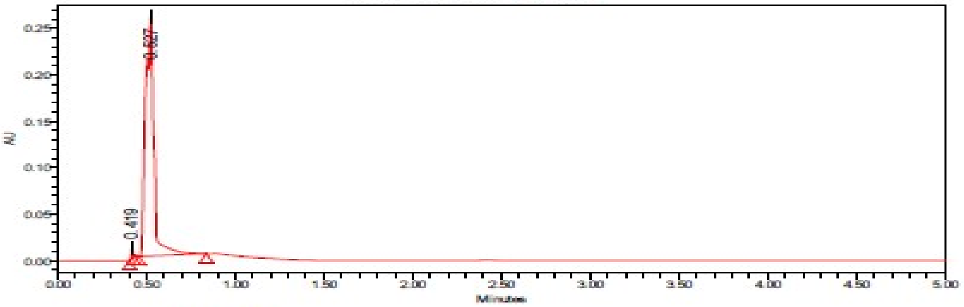

Peak Results

	Name	RT	Area	% Area
1		0.419	5088	0.60
2		0.527	847545	99.40

Reported by User: System
Report Method: siva
Report Method ID: 5560
Page: 1 of 1

Project Name: VIT TBI
Date Printed:
15-03-2019
13:53:21 Asia/Kolkata

ESI-MS spectra

Complex 4a

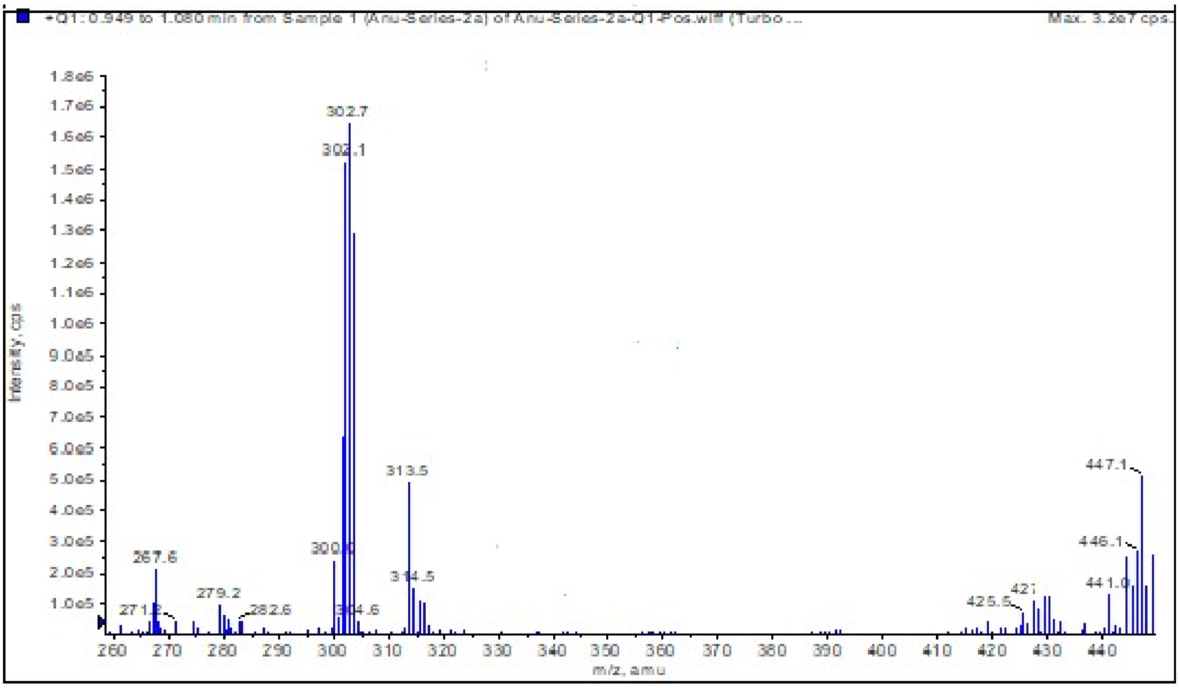

Complex 4b

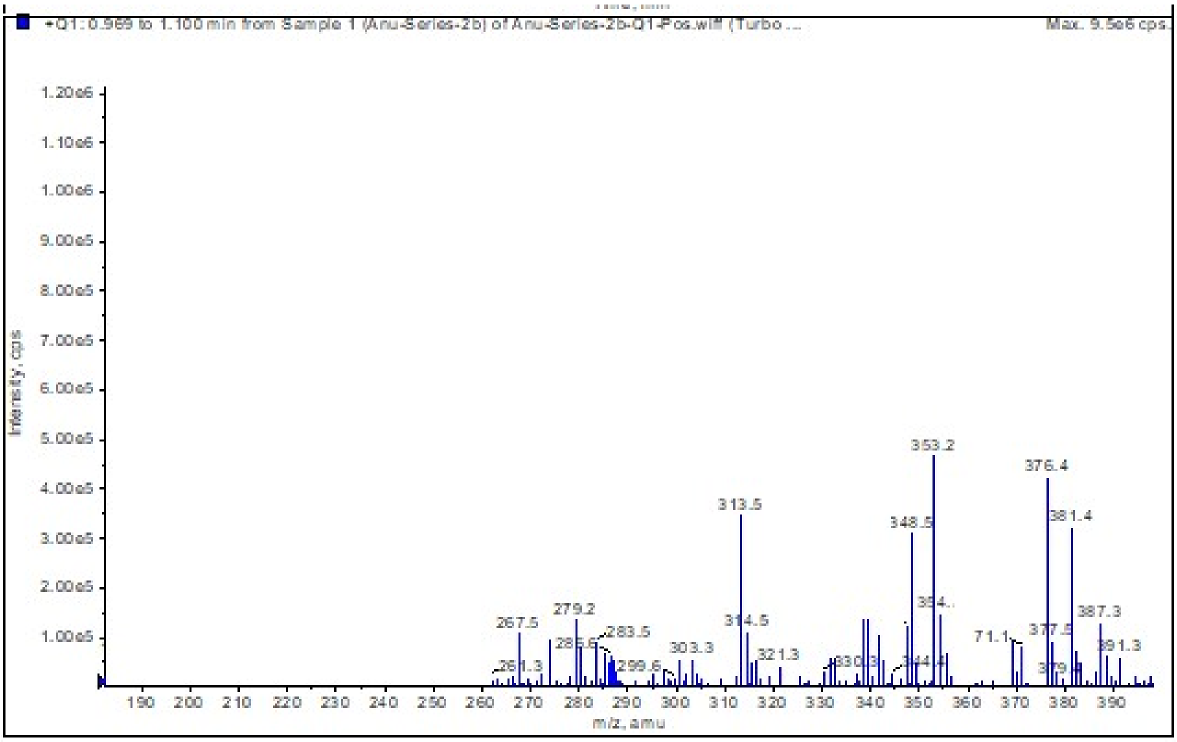

Complex 4c

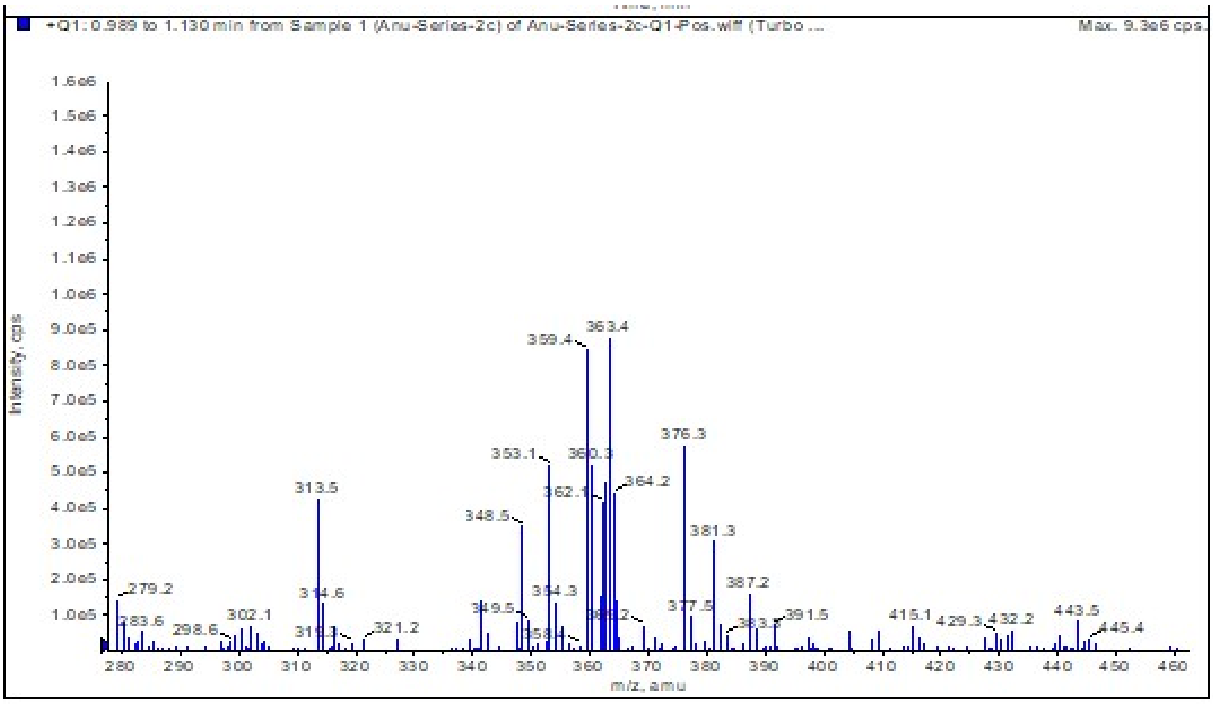

Complex 4d

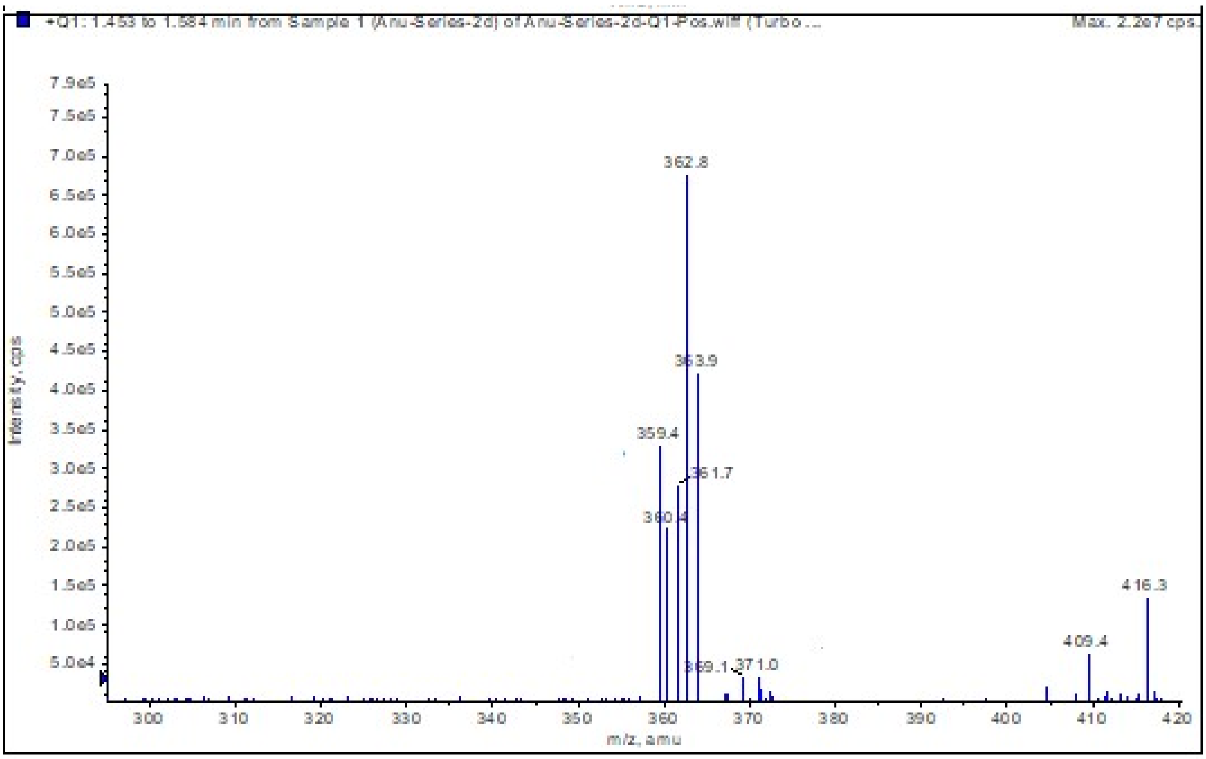

Complex 4e

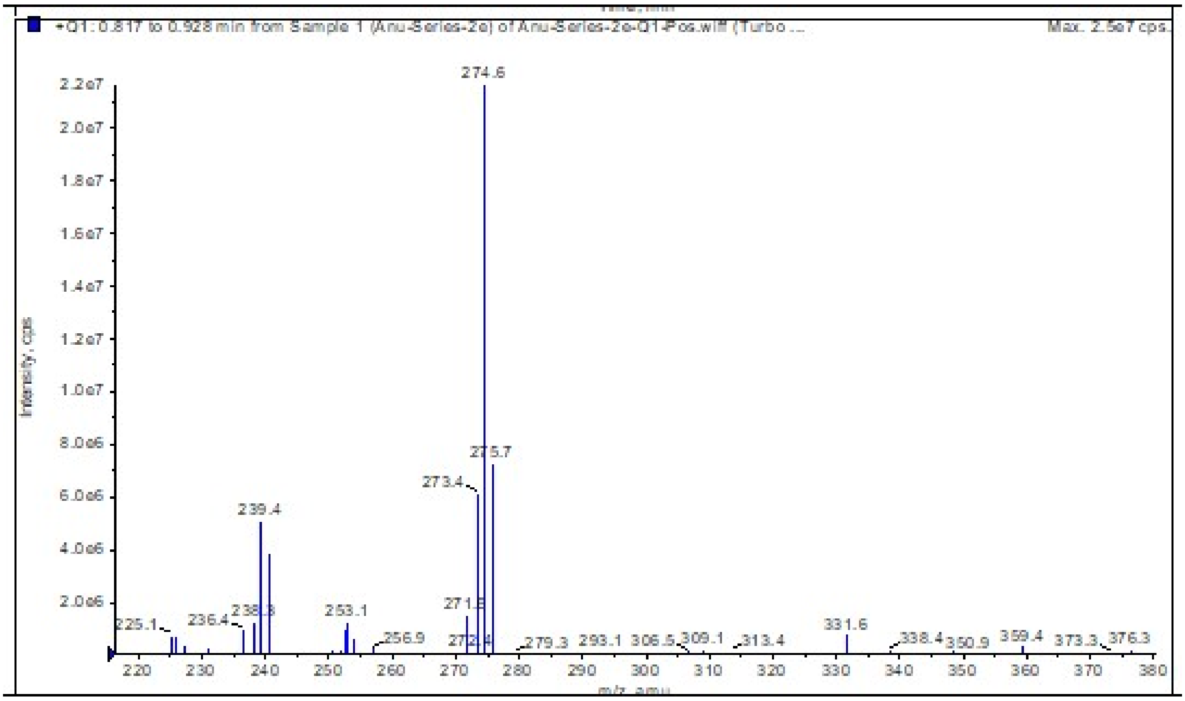

Complex 4g

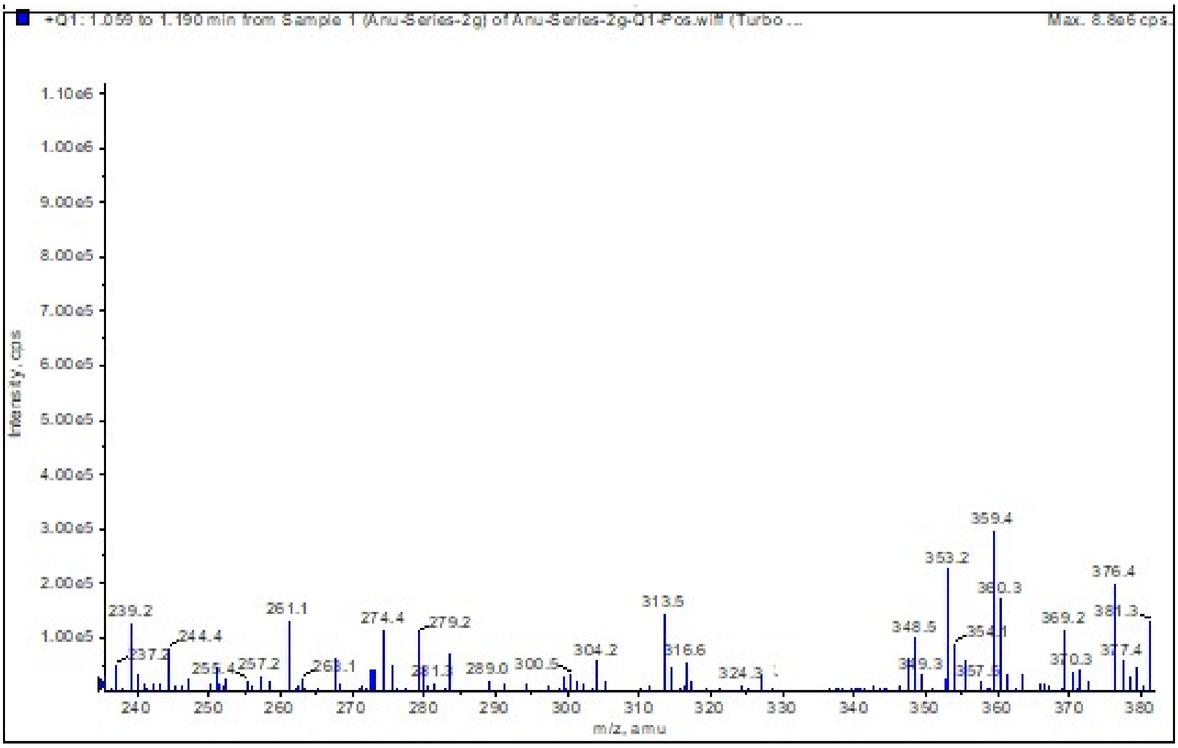

Complex 4h

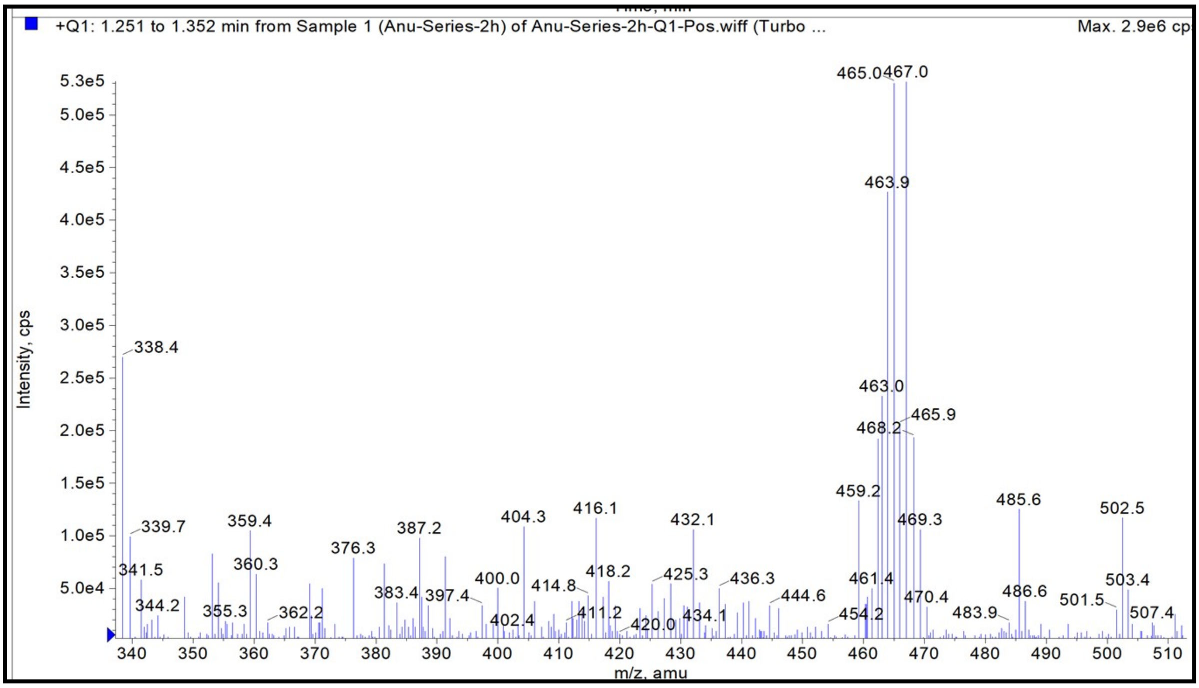

+Q1: 1.251 to 1.352 min from Sample 1 (Anu-Series-2h) of Anu-Series-2h-Q1-Pos.wiff (Turbo ...
Max. 2.9e6 cps
Intensity, cps
m/z, amu
338.4
339.7
341.5
344.2
355.3
359.4
360.3
362.2
376.3
383.4
387.2
397.4
400.0
402.4
404.3
411.2
414.8
416.1
418.2
420.0
425.3
432.1
434.1
436.3
444.6
454.2
459.2
461.4
463.0
463.9
465.0
465.9
467.0
468.2
469.3
470.4
483.9
485.6
486.6
501.5
502.5
503.4
507.4

Experimental Section

DNA binding study

The binding capability of the complexes with calf-thymus DNA (Ct-DNA) was evaluated using electronic absorption spectroscopy, and the competitive binding assay was studied using ethidium bromide (EtBr) as a quencher by fluorescence spectroscopy.

UV–visible studies[1]

The DNA binding experiment was performed in aqueous medium using RAPTA complex **4c** in Tris-HCl buffer (5 mM Tris-HCl in water, pH 7.4). The concentration of Ct-DNA was determined using its absorbance at 260 nm and a known molar absorption coefficient of 6600 $M^{-1}.cm^{-1}$. In cuvettes, an equal amount of DNA was introduced to both the sample and the reference. The concentration of CT-DNA was increased as the titration progressed. The sample was equilibrated with CT-DNA for around 5 minutes before each measurement, and then the complex's absorbance was measured. The intrinsic DNA binding constant (K_b) was calculated using the equation (i):

$$\frac{[DNA]}{(\varepsilon_a - \varepsilon_f)} = \frac{[DNA]}{(\varepsilon_b - \varepsilon_f)} + \frac{1}{K_b(\varepsilon_a - \varepsilon_f)} \ldots\ldots (i)$$

Where [DNA] is the concentration of DNA in the base pairs, ε_a is the apparent extinction coefficient observed for the complex, ε_f corresponds to the extinction coefficient of the complex in its free form, and ε_b refers to the extinction coefficient of the complex when fully bound to DNA. The resultant data were plotted using Origin Lab, version 8.5 to obtain the $[DNA]/(\varepsilon_a-\varepsilon_f)$ vs. [DNA] linear plot. The ratio of the slope to intercept from the linear fit gave the values of the intrinsic binding constants (K_b).

UV and Fluorescence study

All of these RAPTA complexes were studied in a 10% DMSO solution using UV and fluorescence. The fluorescence quantum yields (Φ) were then estimated using the comparative William's approach, which entails utilising a well-characterized standard with a known quantum yield value and a 10% DMSO solution.[2] Quinine sulphate was employed as a standard. Quantum yield was calculated according to the equation (ii):

$$\varphi = \varphi_R \times \frac{I_S}{I_R} \times \frac{OD_R}{OD_S} \times \frac{\eta_S}{\eta_R} \cdots\cdots\cdots (ii)$$

Where, φ = quantum yield, I = peak area, OD = absorbance at λmax, η = refractive index of solvent (s) and reference (R). Here, we have used quinine sulphate as a standard for calculating the quantum yield.

Ethidium bromide displacement assay

To demonstrate the manner of binding between the complexes and DNA, the ethidium bromide (EtBr) displacement experiment was used.[3] Using ethidium bromide (EtBr) as a spectral probe in 5 mM Tris-HCl buffer, the apparent binding constant (K_{app}) of the RAPTA complex **4c** to Ct-DNA was determined at pH = 7.4. Because the fluorescence of EtBr was quenched by the solvent molecules, it was unable to show any fluorescence in its free state. However, in the presence of Ct-DNA, its fluorescence intensity began to increase, indicating that EtBr binds to DNA grooves in an intercalative manner. With increasing concentrations of the complexes, the intensity of the fluorescence was shown to diminish. As a result, the complexes displaced EtBr from CT-DNA grooves and were linked to the DNA base pairs itself. The values of the apparent binding constant (K_{app}) were obtained by using the equation (iii):

$$K_{app} \times [Complex]_{50} = k_{EtBr} \times [EtBr] \cdots\cdots\cdots (iii)$$

Where K_{EtBr} is the EtBr binding constant (K_{EtBr} = 1.0 x 10^{7} M^{-1}), and [EtBr] = 8 x 10^{-6} M. Stern-Volmer equation was followed for quantitative determination of the Stern-Volmer quenching constant (K_{SV}).[4] Origin (8.5) software was used to plot the fluorescence data to obtain linear plot of I_0/I vs. [complex]. The value of K_{SV} was calculated from the following equation.

$$I_0/I = 1 + K_{SV}[Q] \ldots\ldots (iv)$$

Where I_0 = fluorescence intensity in absence of complex and I = fluorescence intensities in presence of complex of concentration [Q].

Protein binding studies[5]

The main component is serum albumin proteins in drug transport and metabolism, as we all know. The interaction of the complex with human serum albumin (BSA) was examined using a tryptophan emission quenching experiment. The association of the RAPTA complexes **4c** and **4d** with the protein BSA was detected using a tryptophan emission quenching assay.

In a Tris-HCl/NaCl buffer, a BSA solution (2 x 10^{-6} M) was first made. Following that, the complex's aqueous solutions were gradually added to the BSA solution, with increasing their concentrations gradually. The solutions were carefully agitated for 5 minutes after each addition before the fluorescence was measured at 295 nm (λex = 295 nm). When the concentration of complex was increased, a gradual drop in BSA fluorescence intensity at 340 nm was detected, confirming that the complex and BSA had interacted. To calculate the quenching constant, the Stern-Volmer equation was used (K_{BSA}). Origin Lab, version 8.5 was used to plot the emission spectral data to obtain linear plot of I_0/I vs. [complex] using the equation (v) given below:

$$I_0/I = 1 + K_{BSA}[Q] = 1 + k_q\tau_0[Q] \ldots\ldots (v)$$

Where I_0 is the fluorescence intensity of HSA in absence of complex and I indicates the fluorescence intensity of HSA in presence of complex of concentration [Q], τ_0 = lifetime of the tryptophan in HSA found as 1 x 10^{-8} and kq is the quenching constant. Scatchard equation (vi) gives the binding properties of the complexes.[6] Where K = binding constant and n = number of binding sites.

$$\log(I_0 - I/I) = \log K + n\log[Q] \ldots\ldots (vi)$$

Conductivity measurement[7]

The conductivity of the RAPTA complexes was measured using a conductivity-TDS meter-307 (Systronics, India) and a cell constant of 1.0 cm^{-1} to verify the interaction of the complexes with DMSO and aqueous DMSO.

n-Octanol–water partition coefficient (log $P_{o/w}$)[8]

Using the previously reported shake flask approach, the log $P_{o/w}$ of the RAPTA complexes followed the previously described procedure. An orbital shaker was used to shake a known amount of each complexes in water (pre-saturated with n-octanol). The solution was centrifuged at 3000 rpm for 10 minutes to allow phase separation. Different ratios (0.5:1, 1:1, and 2:1) of saturated solutions were shaken for 20 minutes on an orbital shaker with pre-saturated n-octanol to get the partition coefficient. The absorbance of aliquots of the aqueous and octanol layers were measured with a UV-Vis spectrophotometer after adequate dilution. The concentrations of the complexes in each layer were estimated using the corresponding

molar extinction coefficients, and the partition coefficient (log $P_{o/w}$) values were computed from the ratio.

Viscosity measurement[9]

A hydrodynamic method such as a viscosity was performed using an Ostwald Viscometer to determine the binding manner of complexes using compound **4c**, **4d**, and EtBr treated DNA with respect to cisplatin.

Notes and References

1. M. Sirajuddin, S. Ali, A. Badshah, Drug–DNA interactions and their study by UV–Visible, fluorescence spectroscopies and cyclic voltammetry, *J. Photochem. Photobio. B.*, 2013, **124**, 1–19.
2. M. Shamsi-Sani, F. Hirini, SM. Abedini, M. Seddighi, Synthesis of benzimidazole and quinoxaline derivatives using reusable sulfonated rice husk ash (RHA-SO_3H) as a green and efficient solid acid catalyst, *Res. Chem. Intermed.*, 2016, **42**, 1091–1099.
3. S. Dasari, A. K. Patra, Luminescent europium and terbium complexes of dipyridoquinoxaline and dipyridophenazine ligands as photosensitizing antennae: structures and biological perspectives, *Dalton Trans.*, 2015, **44**, 19844-19855.
4. (a) J. Keizer, Nonlinear fluorescence quenching and the origin of positive curvature in Stern-Volmer plots, *J. Am. Chem. Soc.*, 1983, **105**, 1494–1498. (b) B. Kar, S. Shanavas, A. H. Nagendra, U. Das, N. Roy, S. Pete, A. Sharma S, S. De, A. Kumar SK, S. Vardhan, S. K. Sahoo, D. Panda, S. Shenoy, B. Bose, P. Paira, Iridium(III)–Cp*-(imidazo[4,5-f][1,10]phenanthrolin-2-yl)phenol analogues as hypoxia active, GSH-resistant cancer cytoselective and mitochondria-targeting cancer stem cell therapeutic agents. *Dalton Trans.*, 2022, **51**, 5494-5514.
5. V. D. Suryawanshi, L. S. Walekar, A. H. Gore, P. V. Anbhule, G. B. Kolekar, Spectroscopic analysis on the binding interaction of biologically active pyrimidine derivative with bovine serum albumin, *J. Pharm. Anal.*, 2016, **6**, 56–63.
6. K. Jeyalakshmi, J. Haribabu, C. Balachandran, S. Swaminathan, N. S. P. Bhuvanesh, R. Karvembu, Coordination Behavior of N,N′,N″-Trisubstituted

Guanidine Ligands in Their Ru–Arene Complexes: Synthetic, DNA/Protein Binding, and Cytotoxic Studies, *Organometallics,* 2019, **38**, 753−770.

7. S. Nikolić, L. Rangasamy, N. Gligorijević, S. Aranđelović, S. Radulović, G. Gasser, S. Grgurić-Šipka, Synthesis, characterization and biological evaluation of novel Ru(II)–arene complexes containing intercalating ligands, *J. Inorg. Biochem.* 2016, **160**, 156–165.
8. M. Kubanik, H. Holtkamp, T. Söhnel, S. M. F. Jamieson, C. G. Hartinger, Impact of the Halogen Substitution Pattern on the Biological Activity of Organoruthenium 8-Hydroxyquinoline Anticancer Agents, *Organometallics*, 2015, **34**, 5658–5668.
9. B. Kar, U. Das, S. De, S. Pete, A. Sharma, N. Roy, A. Kumar. SK, D. Panda, P. Paira, GSH resistant and highly cytoselective Ruthenium(II)-*p*-cymene-imidazo[4,5-f][1,10]phenanthrolin-2-yl)phenol complexes as potential anticancer agent, *Dalton Trans*., 2021, **50**, 10369-10373.
